# ﻿Progress toward a list of saproxylic beetles (Coleoptera) in the southeastern USA

**DOI:** 10.3897/zookeys.1232.143989

**Published:** 2025-03-14

**Authors:** Clayton R. Traylor, Michael D. Ulyshen, J. Winston Cornish, Gabriel Tigreros, Joseph V. McHugh

**Affiliations:** 1 Department of Entomology, University of Georgia, Athens, GA 30602, USA University of Georgia Athens United States of America; 2 Department of Biology, Temple University, Philadelphia, PA 19130, USA Temple University Philadelphia United States of America; 3 USDA Forest Service, Southern Research Station, Athens, GA 30602, USA USDA Forest Service, Southern Research Station Athens United States of America; 4 The Jones Center at Ichauway, Newton, GA 39870, USA The Jones Center at Ichauway Newton United States of America

**Keywords:** Coastal Plain, forest entomology, forest management, oak-pine forest, Piedmont, southern Appalachian Mountains, southern pine forest

## Abstract

Deadwood-dependent (saproxylic) insects represent a large proportion of forest biodiversity, are major contributors to ecosystem processes, and are conservation priorities due to their sensitivity to changing forest conditions. Despite relevance across much of the world, research on saproxylic biodiversity has been concentrated in Europe where interest was first generated. A major impediment for this field elsewhere is a lack of resources to determine which species are saproxylic. Here, we attempt to facilitate research on saproxylic beetles (Coleoptera) in the southeastern USA by compiling information from 18 published studies and theses in the region. A list of 1,393 taxa (species or genera) from 74 families is provided with deadwood associations. This includes 891 taxa from 71 families that were reared or emerged from deadwood, and 831 taxa from 61 families that were collected from bulk trapping methods and considered to be saproxylic, or were previously included in a list of regional deadwood taxa. Additionally, for 293 taxa from non-economically important families that were considered to be saproxylic in a recently published study, known saproxylic habits, microhabitat associations, and conservation notes are listed. Sixty-eight of these species represent new state records in Georgia, USA. Although a checklist of saproxylic species is needed for the southeastern USA, it is precluded by a dearth of knowledge about the natural history and distribution of species in the region. Increasing our understanding of these species’ habitat requirements is essential for understanding biodiversity responses to changing forest conditions and assessing conservation needs.

## ﻿Introduction

Saproxylic insects, those dependent on deadwood and associated resources for all or part of their life-cycle, are important contributors to ecosystem processes and account for a large portion of biodiversity within forests (Stokland et al. 2012). Many of these species aid in wood decomposition and nutrient cycling within forests (Ulyshen 2016), although assemblages are composed of species occupying a variety of functional roles, including phloeophages, xylophages, mycophages, and predators (Ulyshen and Šobotník 2018). Outside of deadwood, many saproxylic insects additionally perform, or are involved in, other ecosystem processes, such as pollination (Ulyshen et al. 2023). Estimates from northern and central Europe suggest that saproxylic species comprise approximately 20–25% of all invertebrates within forests (Siitonen 2001; Graf et al. 2022), although estimates are higher within some taxonomic groups (e.g., 42–65% for Coleoptera; Köhler 2000; Martikainen et al. 2000; Stenbacka et al. 2010; Gibb et al. 2013). Furthermore, many species facultatively use deadwood, increasing the proportion of forest invertebrate species utilizing this resource to 50–70% (Graf et al. 2022). Outside of Europe, the proportion of saproxylic species within forests has only been estimated for Coleoptera in the Atlantic Canada region: 63–79% (Majka 2009). Although saproxylic species occur in most insect orders (Ulyshen 2018), beetles (Coleoptera) are the most studied due to their taxonomic and ecological diversity (Gimmel and Ferro 2018).

Since the seminal work of Speight (1989) documenting the decline of saproxylic insects from intensive forest management and land-use change across Europe, great attention has been devoted to assessing the conservation status of saproxylic insects in the region (Seibold et al. 2015a; Cálix et al. 2018; Hagge et al. 2021) and documenting their basic habitat requirements and ecology (Siitonen and Saaristo 2000; Harvey et al. 2011; Horák et al. 2011; Lindman et al. 2023). Saproxylic communities are sensitive to the amounts and diversity of deadwood resources, and the quality and age of forest habitat more generally (Stenbacka et al. 2010; Gibb et al. 2013; Janssen et al. 2016, 2017; Seibold et al. 2016, 2017). Thus, much effort has been spent developing forest management practices that benefit these species, including deadwood retention or imitation of natural tree mortality dynamics (Thorn et al. 2014; Gossner et al. 2016; Heikkala et al. 2016; Roth et al. 2019; Ekström et al. 2021). Moreover, minimum thresholds of deadwood volume, under which certain species decline, have been determined for certain forest types (Müller and Bütler 2010; Lachat et al. 2012; Gossner et al. 2013). Overall, these efforts have provided evidence-based recommendations for saproxylic insect conservation in Europe.

Although saproxylic insects throughout the world are facing threats of deforestation and intensive forest management, research has been concentrated in Europe where awareness was first generated. But interest is rising in other regions where forests are the dominant vegetation (Davies et al. 2008; Seibold et al. 2015b). For example, researchers in Canada have devoted effort to this field (reviewed in Langor et al. 2008) and have produced conservation-priority lists of saproxylic species (Majka 2007). Similarly, there has been an increase in attention in the southeastern USA to assess host usage and habitat quality (e.g., Campbell et al. 2008a, b; Ulyshen and Hanula 2009a, 2010; Klepzig et al. 2012; Ferro et al. 2012a, b; Traylor et al. 2022a, 2023a, 2024). A global representation of saproxylic research is needed, as deadwood dynamics—to which saproxylic fauna are adapted—vary regionally from climatic and biotic differences (e.g., temperature and termites; Zanne et al. 2022), and the ecological relationships that guide conservation recommendations may change from these biogeographical differences. For example, the relationship between saproxylic insect biodiversity and deadwood volume may change in different climates (Lassauce et al. 2011; Lachat et al. 2012; Seibold and Thorn 2018), as ectothermic larvae can develop within smaller amounts of deadwood in warmer climates (Müller et al. 2015). Thus, it is important for management recommendations to be developed separately for each region based on the sensitivities and ecological roles of the endemic fauna within the context of local climatic conditions.

A major impediment to studying saproxylic insect biodiversity is simply recognizing which species are saproxylic. Decisions about inclusion or exclusion of species from analyses of saproxylic communities may influence the results, as well as the management practices drawn from them. This problem is especially relevant when biodiversity is assessed from trap samples, which collect a wider range of species and functional guilds than sampling directly from substrates (Micó et al. 2020). Currently, regional checklists of saproxylic species have been produced for Europe (Köhler 2000; Schmidl and Bußler 2004) and portions of Canada (e.g., Majka and Pollock 2006; Majka 2007; references cited in Langor et al. 2008). Additionally, resources are available for a few better-known and economically important insect groups in the USA, such as bark beetles (Curculionidae: Scolytinae; Wood 1982), woodboring beetles (Cerambycidae; Lingafelter 2007), and jewel beetles (Buprestidae; Nelson et al. 2008). Aside from these examples, few resources are available to aid in this task in North America as regional checklists with annotated saproxylic associations simply do not exist. Thus, studies of saproxylic biodiversity using trap samples are burdened with finding scattered primary literature documenting the natural history of each species (if any exist). Otherwise, decisions about saproxylic status must be presumed based on current classification and patterns of known natural history for congeneric or confamilial species.

Here, we provide two resources to aid research on saproxylic beetles in the southeastern USA. First, from published studies or theses in the southeastern region, we provide a list of taxa (species or genera) that either 1) emerged or were hand-collected directly from deadwood, 2) were considered to be saproxylic based on a classification process, or 3) were previously included in a regional list of common deadwood taxa. Secondly, we provide a summary of the known deadwood associations of 293 taxa from non-economically important families collected in a survey of saproxylic beetles in Georgia, USA (Traylor et al. 2023a). These resources should facilitate studies of saproxylic biodiversity in the southeastern USA and elsewhere in North America and form the foundation for a regional saproxylic checklist.

## ﻿Methods

### ﻿Saproxylic definition

The definition of “saproxylic” has evolved over time to fit a growing understanding of deadwood resources and the species that depend on them (Alexander 2008; Graf et al. 2022). Today, a saproxylic species is considered “any species that depends, during some part of its life cycle, upon wounded or decaying woody material from living, weakened or dead trees” (Stokland et al. 2012: 6). In addition to species dependent on wood, this definition broadly includes species dependent on non-wood resources found in or on living, weakened, or dead trees, such as the inner bark (phloem), sap runs, or slime fluxes in tree wounds. While it may seem bizarre for saproxylic species to use resources in living trees, it is widely recognized that important microhabitats can occur and accumulate over a tree’s life, such as dead branches in the canopy or interior rot forming tree hollows (Micó 2018; Seibold et al. 2018). Additionally, this definition includes species that depend indirectly on saproxylic resources. For example, species dependent on wood-decaying fungi are considered saproxylic, as well as species representing higher trophic levels (e.g., predators, parasitoids, and parasites) that are dependent on other saproxylic species as hosts or prey (Stokland et al. 2012). Moreover, species that do not consume wood or other saproxylic organisms may still be saproxylic, such as bees that nest in deadwood or tree cavities (Ulyshen 2018).

An important distinction exists between species that are obligately dependent on deadwood and associated resources, versus those that use these habitats facultatively. In general, saproxylic species are those that are obligately dependent, and thus would disappear if those resources were removed from forests (Stokland et al. 2012). However, distinguishing facultative or obligative use of deadwood (i.e., non-saproxylic vs saproxylic) can be difficult and largely depends on incomplete information known about a species’ biology.

### ﻿List of saproxylic Coleoptera compiled from previous studies

From 18 published studies and theses in the southeastern USA (Table 1), we compiled a list of coleopteran taxa (species and genera) that either 1) emerged or were hand-collected directly from deadwood, 2) were considered saproxylic following some explicit determination process, or 3) were included in a previous regional list of common deadwood insects. Fifteen of these studies provided species lists of Coleoptera that either emerged or were hand-collected directly from deadwood in experimental methods (Table 1). Two studies provided lists of species that were considered to be saproxylic after a determination process. Klepzig et al. (2012) used pitfall trapping to collect insects and considered beetle species as saproxylic, non-saproxylic ground beetles, or other based on familiarity with the taxa and published natural history. Traylor et al. (2023a) collected beetles with flight intercept traps and litter-sifting and classified the collected species as saproxylic or not (see below). Finally, one study created a list of wood-feeding beetles that are commonly collected in the southern USA (Hanula 1996). Many other, often older, publications provide accounts of deadwood associations based on field observations (e.g., Savely 1939; Howden and Vogt 1951), but those are not included here. Other studies assumed saproxylic status based on the known natural history of closely related taxa, but did not go through any explicit determination process (e.g., Campbell et al. 2008a, b); therefore, they were not included.

**Table 1.** Experimental studies providing species lists of Coleoptera with deadwood associations. Coleoptera were emergent from deadwood (method = E), hand-collected from deadwood (H), or classified (C) as saproxylic in the southeastern United States. The state(s) and ecoregion(s) sampled from each study are provided. Level III Ecoregions follow https://www.epa.gov/eco-research/ecoregions-north-america. The list of Coleoptera can be found in Suppl. material 1.

Study	State	Type III Ecoregion(s)	Method
Ferro and Gimmel 2014	LA	8.3.6 (Mississippi Valley Loess Plains)	E
Ferro and Nguyen 2016	LA	8.3.5 (Southeastern Plains); 8.3.6 (Mississippi Valley Loess Plains); 8.3.7 (South Central Plains); 8.5.2 (Mississippi Alluvial Plain); 8.5.3 (Southern Coastal Plain); 9.5.1 (Western Gulf Coastal Plain)	E
Ferro et al. 2009	LA	8.3.6 (Mississippi Valley Loess Plains)	E
Ferro et al. 2012a	TN	8.4.4 (Blue Ridge)	E
Ferro et al. 2012b	TN	8.4.4 (Blue Ridge)	E
Garrick et al. 2019	AL, NC, TN	8.4.4 (Blue Ridge); 8.4.9 (Southwestern Appalachians)	H
Gil 2008	LA	8.3.6 (Mississippi Valley Loess Plains)	H
Hanula 1996	southern region		C
Klepzig et al. 2012	LA, MS, NC, TX	8.3.5 (Southeastern Plains); 8.3.7 (South Central Plains); 8.5.1 (Middle Atlantic Coastal Plain)	C
Tigreros 2024	GA	8.3.5 (Southeastern Plains)	E
Traylor et al. 2023a	GA	8.3.4 (Piedmont)	C
Ulyshen 2014	MS	8.3.5 (Southeastern Plains)	E
Ulyshen and Hanula 2009a	SC	8.3.5 (Southeastern Plains)	E
Ulyshen and Hanula 2009b	SC	8.3.5 (Southeastern Plains)	E
Ulyshen and Hanula 2010	SC	8.3.5 (Southeastern Plains)	E
Ulyshen et al. 2010	GA	8.3.4 (Piedmont)	E
Ulyshen et al. 2020	MS	8.3.5 (Southeastern Plains)	E
Ulyshen et al. 2024	SC	8.3.5 (Southeastern Plains)	E

Only species or generic determinations (including subgeneric distinctions, when possible) were included in our lists; morphospecies at the tribe, subfamily, or family rank were excluded. We checked the validity of all taxonomic names using the Integrated Taxonomic Information System (www.ITIS.gov), or with the most recent taxonomic revision of the group or other published literature.

### ﻿Annotated list of saproxylic Coleoptera

We summarized saproxylic habits of 293 coleopteran taxa considered to be saproxylic from Traylor et al. (2023a). Briefly, from March–September 2020 in Clarke County, Georgia, USA, beetles were collected from forests using two methods: 1) a flight intercept trap (two clear, intersecting panels attached over a white bucket) suspended 5 m aboveground and baited with ethanol, and 2) leaf litter sifting and extraction via a Berlese funnel (see Traylor et al. 2022b, 2023a for details). For each species, we provide summary statistics of collection data. Full data of species’ abundance per sampling location, species’ abundance per sampling period for each collection method, and forest details are publicly available at a repository (Traylor et al. 2023b).

The saproxylic determination process was as follows. Species were identified and considered as saproxylic or not based on known natural history information indicating that they either develop in, or are primarily found within, deadwood or other saproxylic habitats. We used Gimmel and Ferro (2018) to exclude some families and subfamilies from saproxylic consideration. Species and morphospecies with no known natural history information were also considered saproxylic if all their congeners with known natural history could be considered saproxylic. See Traylor et al. (2023a) for more information.

Here, we assembled known natural history information for each saproxylic species (or genus for morphospecies) based on published accounts, rearing/emergence records, or verifiable images with documented natural history information from Bugguide (www.bugguide.net). Additionally, we provide specific information on microhabitat usage (if known) and conservation notes (if applicable). Unless stated otherwise, we also provide general distributions for each species from occurrences recorded in the Global Biodiversity Information Facility (GBIF; www.gbif.org) and BugGuide (www.bugguide.net). Non-mutually exclusive distribution categories are defined as follows: the southeastern United States (Virginia south to Florida, west to Arkansas and eastern Texas), the eastern United States (Minnesota south to Louisiana, and eastward), eastern North America (the eastern United States and Canada, Ontario and eastward), central United States (the “plain states”, i.e., Wisconsin south to Texas, and west to Montana and New Mexico), central North America (central United States and Canada, Manitoba west to Alberta). The assembled information is not intended to be a full review of each species’ natural history. Instead, the information is meant to provide a summary of deadwood associations and saproxylic habits exhibited by each species that can be used to aid researchers in classifying species as saproxylic or not and to give some background on required habitats or wood types. Natural history information unrelated to deadwood was not included (e.g., that adult Mordellidae visit flowers). Because natural history and host record reviews already exist for economically important groups in this region, such as Buprestidae (Nelson et al. 2008), Cerambycidae (Lingafelter 2007), and Curculionidae: Scolytinae (Wood 1982), we do not include these taxa below unless they are new state records in Georgia, USA.

## ﻿Results

In total, 1,393 beetle taxa (species or genera) from 74 families had deadwood associations recorded in the 18 studies or theses (Suppl. material 1). This includes 891 taxa from 71 families that were reared or emerged from deadwood, and 831 taxa from 61 families that were collected from bulk trapping methods and considered to be saproxylic or were listed as common associates of deadwood. We urge readers to be cautious in the use of this list, as some species that were reared or emerged from deadwood are likely to be facultatively saproxylic. Additionally, some taxa considered to be saproxylic could be reclassified as facultative users of deadwood if more detailed knowledge of their natural history became available and supported the change.

Below, we provide the deadwood habitat associations of 293 saproxylic beetle taxa (281 species and 12 genera for which only morphospecies were assigned) collected from 40 sites during a survey conducted in the Piedmont region of Georgia, USA (Traylor et al. 2023a). Natural history data relevant to saproxylic habits are summarized for each species, and bionomic information is included when possible. All taxa were considered obligately saproxylic, but as before, additional information learned about the listed taxa in the future may require that some be considered facultative. Sixty-eight species are new state records in Georgia, USA and have been marked as such (* indicates that museum specimens exist from Georgia, but they are not formally published: An Annotated List of the Coleoptera of Georgia, v. 1.0; https://site.caes.uga.edu/ugca/turnbow-and-smith/). Abundance for each species per site with geographic coordinates, per sampling method, and per collection date can be found in a data repository (Traylor et al. 2023b).

### ﻿Aderidae

#### ﻿ Cnopus impressus

Taxon classificationAnimaliaColeopteraAderidae

(LeConte, 1875)

88F04F77-1134-5BC7-BE82-41CD290D0058

##### Collection information.

USA: Georgia (new state record*): Clarke Co.: 22 individuals from 15 sites. Caught in flight trap from 6 May–27 July 2020.

##### Distribution.

Eastern North America, possibly further west.

##### Saproxylic habits.

Emerged from and associated with loblolly pine (*Pinustaeda* L. (Pinaceae)), also emerged from sweetgum (*Liquidambarstyraciflua* L. (Altingiaceae)) (Ulyshen and Hanula 2009a); adults occur in dead tree trunks (Ciegler 2014).

##### Conservation.

In the Piedmont, significantly associated with forests within highly forested landscapes (> 50% forest; Traylor et al. 2023a) and occurrence probability increases with the amount of landscape forest cover (Traylor et al. 2024).

#### ﻿ Ganascus ventricosus

Taxon classificationAnimaliaColeopteraAderidae

(LeConte, 1875)

FD5DE6F9-AE88-59E8-97F5-7F80389E6BB5

##### Collection information.

USA: Georgia: Clarke Co.: five individuals from five sites. Caught in flight trap from 6 May–14 July 2020.

##### Distribution.

Eastern North America.

##### Saproxylic habits.

Emerged from loblolly pine and sweetgum logs (Ulyshen and Hanula 2009a); adults occur in rotten wood, such as black oak (*Quercusvelutina* Lam. (Fagaceae)) (Palmer 2017).

#### ﻿ Pseudariotus notatus

Taxon classificationAnimaliaColeopteraAderidae

(LeConte, 1855)

9B33A9F4-E50D-53F6-B21D-9EDBF3C9919E

##### Collection information.

USA: Georgia: Clarke Co.: two individuals from two sites. Caught in flight trap from 14–27 July 2020.

##### Distribution.

Southeastern United States.

##### Saproxylic habits.

Emerged from loblolly pine, including logs dead for five years, as well as burned and unburned logs (Ulyshen and Hanula 2010; Ulyshen et al. 2010).

### ﻿Anamorphidae

#### ﻿Genus Anamorphus

Taxon classificationAnimaliaColeopteraAnamorphidae

LeConte, 1878

E17B5BFC-8AA2-51D9-A088-202F79D0C7FE

##### Collection information.

USA: Georgia: Clarke Co.: two individuals from two sites. Caught in flight trap from 19 May–14 July 2020. Specimens unable to be identified to species.

##### Saproxylic habits.

Species of *Anamorphus* have emerged from hardwood twigs (*Anamorphuswaltoni* Blatchley, 1918) (Ferro and Nguyen 2016).

#### ﻿ Clemmus minor

Taxon classificationAnimaliaColeopteraAnamorphidae

(Crotch, 1873)

B3906100-21E5-5650-868D-67756E2C2B90

##### Collection information.

USA: Georgia: Clarke Co.: a single individual from one site. Caught in flight trap from 21 April–6 May 2020.

##### Distribution.

Southeastern and central United States.

##### Saproxylic habits.

Emerged from hardwood twigs (Ferro and Nguyen 2016); adults occur under and inside fallen, rotting logs (Gruber 2008).

#### ﻿ Micropsephodes lundgreni

Taxon classificationAnimaliaColeopteraAnamorphidae

Leschen and Carlton, 2000

80FA72C7-6361-5B1C-A411-19AE57C60AE6

##### Collection information.

USA: Georgia: Clarke Co.: seven individuals from five sites. Caught in flight trap from 21 April–26 August 2020.

##### Distribution.

Southeastern United States.

##### Saproxylic habits.

Emerged from sweetgum logs and snags (standing dead trees) (Ulyshen and Hanula 2009a), recently dead coarse woody debris (Ferro et al. 2012a), and twig bundles of southern red oak (*Quercusfalcata* Michx.) (Ferro and Gimmel 2014); for more information see Shockley et al. (2008).

### ﻿Anthribidae

#### ﻿ Choragus zimmermanni

Taxon classificationAnimaliaColeopteraAnthribidae

LeConte, 1876

47C4A4F8-E014-5A27-BD6A-CC13095CBF46

##### Collection information.

USA: **Georgia (new state record)**: Clarke Co.: two individuals from two sites. Caught in flight trap from 16 June–14 July 2020.

##### Distribution.

Eastern North America (Valentine 1998).

##### Saproxylic habits.

Breed in deadwood, such as sweetgum (Brues 1927); species of *Choragus* Kirby, 1819 feed on ascomycete fungi (Xylariaceae and Diatrypaceae) (Valentine 1998).

#### ﻿ Euparius marmoreus

Taxon classificationAnimaliaColeopteraAnthribidae

(Olivier, 1795)

BD3CBBFA-132F-534A-AA97-2F2DE25AE4EB

##### Collection information.

USA: Georgia: Clarke Co.: a single individual from one site. Caught in flight trap from 27 July–11 August 2020.

##### Distribution.

Eastern and central North America.

##### Saproxylic habits.

Feeds on a variety of polypore fungi, including *Trametes* Fr. (Polyporaceae), *Megasporoporiasetulosa* (Henn.) (Polyporaceae), *Trichaptum* Murrill (Hymenochaetales), *Scopuloideshydnoides* (Cooke and Massee) Hjortstam and Ryvarden (Meruliaceae), *Panusrudis* Fr. (Polyporaceae), and *Perenniporiamedulla-panis* (Jacq.) Donk (Polyporaceae) (Valentine 1998).

#### ﻿ Eusphyrus walshi

Taxon classificationAnimaliaColeopteraAnthribidae

LeConte, 1876

FDD184BB-7AC3-54F0-9B81-DDFDF2F82B17

##### Collection information.

USA: Georgia: Clarke Co.: eight individuals from six sites. Caught in flight trap from 19 May–9 September 2020.

##### Distribution.

Eastern North America.

##### Saproxylic habits.

Emerged from poison ivy vines (*Toxicodendron* Mill. (Anacardiaceae), and adults occur on dead hardwood branches and under bark, such as oak (*Quercus* L.) (Valentine 1998).

#### ﻿ Euxenus jordani

Taxon classificationAnimaliaColeopteraAnthribidae

Valentine, 1991

6A388DE6-0EA3-5864-8CC7-86E21ECB85D2

##### Collection information.

USA: **Georgia (new state record)**: Clarke Co.: two individuals from a single site. Caught in flight trap from 2 June–26 August 2020.

##### Distribution.

Eastern North America (Valentine 1998).

##### Saproxylic habits.

Emerged from the fungus *Biscogniauxiaatropunctata* (Schwein.) Pouzar (Graphostromataceae) (Valentine 1998).

#### ﻿ Euxenus punctatus

Taxon classificationAnimaliaColeopteraAnthribidae

LeConte, 1876

569A8B55-1C7E-5F53-AE61-0C3CE25E266B

##### Collection information.

USA: **Georgia (new state record)**: Clarke Co.: seventeen individuals from 13 sites. Caught in flight trap from 30 June–26 August 2020.

##### Distribution.

Eastern North America.

##### Saproxylic habits.

Emerged from *Hypoxylonperforatum* (Schwein.) Fr. (Hypoxylaceae) and adults occur on similar fungi growing on dead branches (Valentine 1998).

##### Conservation.

Occurrence probability increases with the amount of landscape forest cover in the Piedmont (Traylor et al. 2024).

#### ﻿ Goniocloeus bimaculatus

Taxon classificationAnimaliaColeopteraAnthribidae

(Olivier, 1795)

9A38EDD0-DEC0-58E1-AD30-512733E4750B

##### Collection information.

USA: Georgia: Clarke Co.: a single individual from one site. Caught in flight trap from 6–19 May 2020.

##### Distribution.

Eastern North America.

##### Saproxylic habits.

Adults occur under bark and on various fungi growing on hardwood trees, including *Biscogniauxia* Kuntze, *Xylaria* Hill ex Schrank (Xylariaceae), and *Diatrype* Fr. (Diatrypaceae) (Valentine 1998).

#### ﻿ Ischnocerus infuscatus

Taxon classificationAnimaliaColeopteraAnthribidae

Fåhraeus, 1839

2FA83BED-E9BF-5B76-97D7-EB5E5E69FF8E

##### Collection information.

USA: Georgia (new state record*): Clarke Co.: three individuals from two sites. Caught in flight trap from 16 June–26 August 2020.

##### Distribution.

Southeastern United States to Central America, and Caribbean islands.

##### Saproxylic habits.

Emerged from deadwood of several hardwood trees, apparently without fungi (Valentine 1998).

#### ﻿ Ormiscus saltator

Taxon classificationAnimaliaColeopteraAnthribidae

LeConte, 1876

A240FF40-4E3E-59A6-B4FF-E206DB7559C8

##### Collection information.

USA: Georgia (new state record*): Clarke Co.: a single individual from one site. Caught in flight trap from 19 May–2 June 2020.

##### Distribution.

Eastern North America.

##### Saproxylic habits.

Breeds in dead wood of deciduous trees (Pierce 1930).

#### ﻿ Piesocorynus moestus

Taxon classificationAnimaliaColeopteraAnthribidae

(LeConte, 1824)

2DB0E10F-6475-5749-84DE-25E824919007

##### Collection information.

USA: Georgia: Clarke Co.: a single individual from one site. Caught in flight trap from 30 June–14 July 2020.

##### Distribution.

Eastern North America.

##### Saproxylic habits.

Adults and larvae of *Piesocorynus* Dejean, 1834 feed on various fungi in the order Xylariales (e.g., *Hypoxylon* Bull.) (Valentine 1998); adults occur on old logs and under bark (Pierce 1930).

#### ﻿ Piesocorynus plagifer

Taxon classificationAnimaliaColeopteraAnthribidae

Jordan, 1904

F3827379-1A80-518D-9868-7773F95A8085

##### Collection information.

USA: Georgia: Clarke Co.: a single individual from one site. Caught in flight trap from 11–26 August 2020.

##### Distribution.

Eastern North America into Central America.

##### Saproxylic habits.

Adults and larvae of *Piesocorynus* feed on various fungi in the order Xylariales (e.g., *Hypoxylon*) (Valentine 1998); adults occur on fungus growing on dead tree trunks and under loose bark (Pierce 1930).

#### ﻿ Toxonotus cornutus

Taxon classificationAnimaliaColeopteraAnthribidae

(Say, 1831)

6A1C0F4A-F0F7-553E-BF79-3E8C6D6FBE4A

##### Collection information.

USA: Georgia: Clarke Co.: 10 individuals from nine sites. Caught in flight trap from 9 March–14 July 2020.

##### Distribution.

Eastern North America.

##### Saproxylic habits.

Bores into dead wood, such as persimmon (*Diospyros* L. (Ebenaceae)), and adults also occur on dead trees, including white oak (*Quercusalba* L.) stems (Valentine 1998).

### ﻿Bostrichidae

#### ﻿ Lichenophanes bicornis

Taxon classificationAnimaliaColeopteraBostrichidae

(Weber, 1801)

26C8D045-22F2-557A-BA73-155819686EBB

##### Collection information.

USA: Georgia: Clarke Co.: a single specimen from one site. Caught in flight trap from 19 May–2 June 2020.

##### Distribution.

Eastern and central North America.

##### Saproxylic habits.

Emerged from hardwood twigs and branches (Hoffmann 1942; Ferro and Nguyen 2016); adults occur under bark of dead hardwood trees (Downie and Arnett 1996).

##### Conservation.

Rare in the Maritime Provinces of Canada, possibly due to the history of intensive forest management in the region altering forest composition and structure (Majka 2007).

#### ﻿ Micrapate cristicauda

Taxon classificationAnimaliaColeopteraBostrichidae

Casey, 1898

9432B95F-B71A-549E-B333-4D44F1211F66

##### Collection information.

USA: **Georgia (new state record)**: Clarke Co.: a single individual from one site. Caught in flight trap from 21 April–6 May 2020.

##### Distribution.

Southeastern United States.

##### Saproxylic habits.

Emerged and adults collected from dead muscadine vine (*Vitisrotundifolia* Michx. (Vitaceae)) (Fischer 1950; Beiriger 2008).

#### ﻿ Xylobiops basilaris

Taxon classificationAnimaliaColeopteraBostrichidae

(Say, 1824)

072F0574-348A-5E3D-8258-BF72C398A00E

##### Collection information.

USA: Georgia: Clarke Co.: 147 individuals from 25 sites. Caught in flight trap from 9 March–9 September 2020.

##### Distribution.

Eastern North America, west to southern California and south to Mexico.

##### Saproxylic habits.

Larvae bore into the sapwood of a wide variety of dead and dying hardwood trees, and occasionally conifers (Solomon 1995); prefers smaller branches (Hoffmann 1942).

### ﻿Bothrideridae

#### ﻿ Bothrideres cryptus

Taxon classificationAnimaliaColeopteraBothrideridae

Stephan, 1989

A6BAB3B6-FE5B-5BA7-AB6E-F9AF5ED826DF

##### Collection information.

USA: Georgia (new state record*): Clarke Co.: two individuals from two sites. Caught in flight trap from 21 April–2 June 2020.

##### Distribution.

Eastern North America (Stephan 1989).

##### Saproxylic habits.

Occur under the dry bark of dead oaks, where it parasitizes cerambycid larvae of the genus *Parelaphidion* Skiles, 1985 (Stephan 1989).

#### ﻿ Bothrideres geminatus

Taxon classificationAnimaliaColeopteraBothrideridae

(Say, 1826)

D8E133C5-3BAC-5FAB-9A21-953A96581702

##### Collection information.

USA: Georgia: Clarke Co.: four individuals from four sites. Caught in flight trap from 9 March–16 June 2020.

##### Distribution.

Eastern and central North America.

##### Saproxylic habits.

Larvae occur under the dry bark of dead hardwood trees (Stephan 1989), parasitizing the larvae of *Chrysobothris* Eschscholtz, 1829 (Buprestidae) (Craighead 1920).

##### Conservation.

Significantly associated with old forests (predating 1938 and oak dominated) in highly forested landscapes (> 50% forest) in the Piedmont (Traylor et al. 2023a).

### ﻿Carabidae

#### ﻿ Mioptachys flavicauda

Taxon classificationAnimaliaColeopteraCarabidae

(Say, 1823)

807C6183-F0C2-5ED5-AF8C-72409B38D7BD

##### Collection information.

USA: Georgia: Clarke Co.: two individuals from two sites. Caught in flight trap from 21 April–11 August 2020.

##### Distribution.

North America.

##### Saproxylic habits.

Predator of small arthropods under bark (Ferro et al. 2012a); emerged from a wide variety of deadwood substrates, including logs, standing dead trees, and coarse and fine woody debris of loblolly pine, sweetgum, oak and various hardwoods (Ulyshen and Hanula 2009a; Ferro et al. 2012a; Ferro and Nguyen 2016); emerged from burned and unburned loblolly pine logs (Ulyshen et al. 2010), and loblolly pine and hardwoods across decomposition stages (Ulyshen and Hanula 2010; Ferro et al. 2012a); associated with logs and portions of standing dead trees near to the ground (Ulyshen and Hanula 2009a).

### ﻿Cerambycidae

#### ﻿ Saperda imitans

Taxon classificationAnimaliaColeopteraCerambycidae

Felt and Joutel, 1904

4E3F3829-302A-5379-8B26-15E80CCE9AE0

##### Collection information.

USA: Georgia (new state record*): Clarke Co.: two individuals from two sites. Caught in flight trap from 19 May–30 June 2020.

##### Distribution.

Eastern North America.

##### Saproxylic habits.

Larvae feed in various hardwood trees such as hickory (*Carya* Nutt. (Juglandaceae)), cherry (*Prunus* L. (Rosaceae)), and willow (*Salix* L. (Salicaceae)) (Lingafelter 2007); in black cherry (*Prunusserotina* Ehrh.), *S. Imitans* prefers larger diameter branches with phloem still present (DiGirolomo et al. 2011).

### ﻿Cerylonidae

#### ﻿ Cerylon castaneum

Taxon classificationAnimaliaColeopteraCerylonidae

(Say, 1826)

5610E64C-9F7C-5E32-B6EF-226F9B34E484

##### Collection information.

USA: Georgia: Clarke Co.: four individuals from three sites. Caught in flight trap from 9 March–19 May 2020.

##### Distribution.

Eastern North America, transcontinental in the north.

##### Saproxylic habits.

Feed on spores and hyphae of fungi growing on and under the bark of dead trees (Lawrence and Stephan 1975); emerged from hardwood logs (Ferro et al. 2012a), and adults occur under bark after four to six years of decomposition (in hickory, Blackman and Stage 1924).

##### Conservation.

Significantly higher abundance in primary (= old-growth) than secondary (= second-growth) forests in the southern Appalachian Mountains (Ferro et al. 2012a).

#### ﻿ Cerylon unicolor

Taxon classificationAnimaliaColeopteraCerylonidae

(Ziegler, 1845)

4DB106EC-D409-5D25-9376-4509576BADA1

##### Collection information.

USA: Georgia: Clarke Co.: 13 individuals from 10 sites. Caught in flight trap from 25 March–9 September 2020.

##### Distribution.

North America.

##### Saproxylic habits.

Adults occur under the bark of various dead hardwood and conifer trees and on fungi, and larvae develop under the bark of hardwoods (Lawrence and Stephan 1975); emerged from loblolly pine, water oak (*Quercusnigra* L.), and especially sweetgum, and associated with portions of dead trees close to the ground (Ulyshen and Hanula 2009a).

### ﻿Ciidae

#### ﻿Genus Cis

Taxon classificationAnimaliaColeopteraCiidae

Latreille, 1797

2FEBE5FE-33AF-59EE-9006-1E8BCB04599F

##### Collection information.

USA: Georgia: Clarke Co.: 36 individuals from 25 sites. Caught in flight trap and sifted from leaf litter from 9 March–26 August 2020. Includes 10 morphospecies that were not determined to species-level.

##### Saproxylic habits.

Larvae and adults live and feed on wood-decomposing bracket fungi (Thayer and Lawrence 2002); although *Cis* can be found in a wide variety of bracket fungi, species typically have a narrow range of related fungal hosts (Orledge and Reynolds 2005), and host use for North American species is reviewed in Lawrence (1973).

#### ﻿Genus Octotemnus

Taxon classificationAnimaliaColeopteraCiidae

Mellié, 1847

2D45CCEA-5DD9-52DF-9A48-EF4000152354

##### Collection information.

USA: Georgia: Clarke Co.: three individuals from three sites. Caught in flight trap from 9 March–27 July 2020. Includes one morphospecies that was not determined to species-level.

##### Saproxylic habits.

Larvae and adults live and feed on wood-decomposing, bracket fungi (Thayer and Lawrence 2002); most *Octotemnus* have a narrow range of related fungal hosts and fall within the *Trametes* ciid host-group (Lawrence 1973; Orledge and Reynolds 2005).

#### ﻿Genus Orthocis

Taxon classificationAnimaliaColeopteraCiidae

Casey, 1898

486CFD7C-5179-50AB-AF81-441C5014AEF5

##### Collection information.

USA: Georgia: Clarke Co.: a single individual from one site. Caught in flight trap from 21 April–6 May 2020. Includes one morphospecies that was not determined to species-level.

##### Saproxylic habits.

Larvae and adults live and feed on wood-decomposing, bracket fungi (Thayer and Lawrence 2002); *Orthocis* have a narrow range of related fungal hosts and fall within the *Auricularia* Bull. (Auriculariaceae) ciid host-group (Lawrence 1973; Orledge and Reynolds 2005).

### ﻿Cleridae

#### ﻿ Chariessa pilosa

Taxon classificationAnimaliaColeopteraCleridae

(Forster, 1781)

47DE13E0-5247-5B4B-BFD4-ECA8B9B332AA

##### Collection information.

USA: Georgia: Clarke Co.: 25 individuals from 13 sites. Caught in flight trap from 21 April–14 July 2020.

##### Distribution.

Eastern and central North America.

##### Saproxylic habits.

Larvae and adults prey upon bark and woodboring beetles (Blackman and Stage 1924; Savely 1939; Hoffmann 1942); emerged from a wide variety of hardwood trees, and frequently occur on downed logs (Opitz 2017).

##### Conservation.

Significantly associated with, and occurrence probability increases in, old forests (predating 1938 and oak dominated) in the Piedmont (Traylor et al. 2023a, 2024).

#### ﻿ Cregya mixta

Taxon classificationAnimaliaColeopteraCleridae

(LeConte, 1866)

750CCCC1-35EE-5D02-B903-19795A2C2EBF

##### Collection information.

USA: Georgia: Clarke Co.: 11 individuals from seven sites. Caught in flight trap from 16 June–9 September 2020.

##### Distribution.

Eastern United States.

##### Saproxylic habits.

Prey upon bostrichid beetles boring through dry, seasoned hardwoods, such as *Xylobiops* Casey, 1898 (Knull 1951), and occurs in association with others such as *Lyctus* Fabricius, 1792 (Opitz 2019).

#### ﻿ Cregya oculata

Taxon classificationAnimaliaColeopteraCleridae

(Say, 1835)

4CA7B235-A96F-5850-8DD0-452744D227D1

##### Collection information.

USA: Georgia: Clarke Co.: 34 individuals from 19 sites. Caught in flight trap from 19 May–26 August 2020.

##### Distribution.

Eastern North America.

##### Saproxylic habits.

Prey upon bark and woodboring beetles in pine (*Pinus* L. (Pinaceae)) and various hardwoods (Knull 1951; Opitz 2019).

##### Conservation.

Occurrence probability increases with the amount of landscape forest cover in the Piedmont (Traylor et al. 2024).

#### ﻿ Cymatodera bicolor

Taxon classificationAnimaliaColeopteraCleridae

(Say, 1825)

289AA6A1-E470-5AB7-AEA7-C873BF873289

##### Collection information.

USA: Georgia: Clarke Co.: a single individual from one site. Caught in flight trap from 9–21 April 2020.

##### Distribution.

Eastern North America.

##### Saproxylic habits.

Prey upon bark and woodboring beetles and occurs under bark of various hardwoods, including hickory, and “cedar” (presumably *Juniperus* L. (Cupressaceae)) (Hopkins 1893; Knull 1951; Dorshorst and Young 2008).

#### ﻿ Cymatodera inornata

Taxon classificationAnimaliaColeopteraCleridae

(Say, 1835)

19B98D1B-07B9-56BF-A78E-0F1CCF04F010

##### Collection information.

USA: Georgia: Clarke Co.: three individuals from three sites. Caught in flight trap from 21 April–2 June 2020.

##### Distribution.

Eastern United States.

##### Saproxylic habits.

Likely a predator of woodboring beetles; it has emerged from hickory logs and oak twigs (Blackman and Stage 1924; Ferro and Gimmel 2014), and under bark of pine (Hopkins 1893).

#### ﻿ Enoclerus ichneumoneus

Taxon classificationAnimaliaColeopteraCleridae

(Fabricius, 1777)

F3C34C97-600D-503D-9FF0-8BDAC2AE09A0

##### Collection information.

USA: Georgia: Clarke Co.: 17 individuals from 13 sites. Caught in flight trap from 9 March–27 July 2020.

##### Distribution.

Eastern North America.

##### Saproxylic habits.

Larvae and adults prey upon bark and woodboring beetles, and occur under bark and within host galleries in a number of hardwood trees and *Juniperus* (Böving and Champlain 1920; Knull 1951).

##### Conservation.

Occurrence probability increases in old forests (predating 1938 and oak dominated) and with the amount of landscape forest cover in the Piedmont (Traylor et al. 2024).

#### ﻿ Enoclerus nigripes

Taxon classificationAnimaliaColeopteraCleridae

(Say, 1823)

49723F3D-4343-56C8-94B3-CF51B95D3685

##### Collection information.

USA: Georgia: Clarke Co.: 10 individuals from nine sites. Caught in flight trap from 9 March–19 May 2020.

##### Distribution.

Eastern and Central North America.

##### Saproxylic habits.

Larvae and adults prey uopn bark and woodboring beetles and occur under bark and on logs infested with prey, including oak, hickory, cedar, spruce (*Picea* A.Dietr. (Pinaceae)), and especially pine (Knull 1951; Majka 2006; Dorshorst and Young 2008).

##### Conservation.

Occurrence probability increases with the amount of landscape forest cover in the Piedmont (Traylor et al. 2024).

#### ﻿ Madoniella dislocata

Taxon classificationAnimaliaColeopteraCleridae

(Say, 1825)

9F537816-ED64-5DCC-8EE1-D830F72CA21A

##### Collection information.

USA: Georgia: Clarke Co.: 86 individuals from 31 sites. Caught in flight trap from 21 April–26 August 2020.

##### Distribution.

Eastern North America.

##### Saproxylic habits.

Prey upon bark and woodboring beetles, and occurs within host galleries and under bark, including a wide variety of hardwood and conifers (Knull 1951; Majka 2006; Dorshorst and Young 2008).

##### Conservation.

Occurrence probability increases in old forests (predating 1938 and oak dominated) in the Piedmont (Traylor et al. 2024).

#### ﻿ Neorthopleura thoracica

Taxon classificationAnimaliaColeopteraCleridae

(Say, 1823)

91F82601-70A0-5E25-8B3F-7660B06FA54C

##### Collection information.

USA: Georgia: Clarke Co.: 11 individuals from seven sites. Caught in flight trap from 6 May–14 July 2020.

##### Distribution.

Eastern North America.

##### Saproxylic habits.

Larvae and adults prey on bark and woodboring beetles in various hardwood trees, especially oak (Böving and Champlain 1920; Ulyshen and Hanula 2009a).

##### Conservation.

Occurrence probability increases with the amount of landscape forest cover in the Piedmont (Traylor et al. 2024).

#### ﻿ Phyllobaenus humeralis

Taxon classificationAnimaliaColeopteraCleridae

(Say, 1823)

52D0BEBC-9BFF-5371-B98A-50E68A811934

##### Collection information.

USA: Georgia: Clarke Co.: six individuals from two sites. Caught in flight trap from 9 April–16 June 2020.

##### Distribution.

Eastern and central North America.

##### Saproxylic habits.

Larvae develop within dead trees and shrubs, including oak, hickory, and sweetfern (*Comptoniaperegrina* (L.) Coult. (Myricaceae)) (Pulaski 1979; Mawdsley 2002), and adults commonly occur on oak (Dorshorst and Young 2008).

#### ﻿ Phyllobaenus pallipennis

Taxon classificationAnimaliaColeopteraCleridae

(Say, 1825)

E1D22B6C-0D80-51D2-977F-703D9EE7D3DE

##### Collection information.

USA: Georgia (new state record*): Clarke Co.: 40 individuals from 13 sites. Caught in flight trap from 16 June–9 September 2020.

##### Distribution.

Eastern North America.

##### Saproxylic habits.

Develop within branches and trunks of oaks and hickories (Mawdsley 2002).

#### ﻿ Phyllobaenus unifasciatus

Taxon classificationAnimaliaColeopteraCleridae

(Say, 1825)

286EDF5E-735A-5A28-B004-E76B526FA426

##### Collection information.

USA: Georgia: Clarke Co.: a single individual from one site. Caught in flight trap from 6–19 May 2020.

##### Distribution.

Eastern United States.

##### Saproxylic habits.

Adults and larvae are predaceous on bark and woodboring beetles and occur under bark and in prey galleries primarily in hardwood trees (Böving and Champain 1920; Knull 1951; Dolphin et al. 1972; Mawdsley 2002); larvae additionally occur within woodier stem galls of cynipid wasps on oak, although food sources in this microhabitat are uncertain (e.g., Eliason and Potter 2000).

#### ﻿ Phyllobaenus verticalis

Taxon classificationAnimaliaColeopteraCleridae

(Say, 1835)

8D85C503-6EFC-53E3-B2B3-D4B5336DF30B

##### Collection information.

USA: Georgia: Clarke Co.: 65 individuals from 24 sites. Caught in flight trap from 21 April–30 June 2020.

##### Distribution.

Eastern North America.

##### Saproxylic habits.

Develops within and prey upon woodboring beetles in dead hardwoods (Knull 1932; Mawdsley 2002); larvae additionally occur within woodier stem galls of cynipid wasps on oak, although food sources in this microhabitat are uncertain (e.g., Eliason and Potter 2000).

#### ﻿ Placopterus thoracicus

Taxon classificationAnimaliaColeopteraCleridae

(Olivier, 1795)

55A344DC-35E3-5D7B-92B0-C8CA287BC35A

##### Collection information.

USA: Georgia: Clarke Co.: 40 individuals from 25 sites. Caught in flight trap from 25 March–19 May 2020.

##### Distribution.

Eastern North America.

##### Saproxylic habits.

Larvae and adults prey upon bark beetles, woodboring beetles, and twig nesting wasps in small dead branches of hardwood trees and shrubs (Böving and Champlain 1920; Knull 1932; Foster and Barr 1972); larvae additionally occur within woodier stem galls of cynipid wasps on oak, although food sources in this microhabitat are uncertain (e.g., Eliason and Potter 2000).

#### ﻿ Pyticeroides laticornis

Taxon classificationAnimaliaColeopteraCleridae

(Say, 1835)

E09A33E7-90A7-5911-9ADC-756058046E15

##### Collection information.

USA: Georgia: Clarke Co.: 31 individuals from 17 sites. Caught in flight trap from 9 April–26 August 2020.

##### Distribution.

Eastern North America.

##### Saproxylic habits.

Larvae and adults prey upon bark beetles within a variety of hardwoods and cedar (Böving and Champlain 1920; Knull 1951).

### ﻿Cucujidae

#### ﻿ Pediacus subglaber

Taxon classificationAnimaliaColeopteraCucujidae

LeConte, 1854

F91907C7-9F4F-52CE-BBD8-A0D48ADBFB34

##### Collection information.

USA: Georgia: Clarke Co.: three individuals from three sites. Caught in flight trap from 9–25 March 2020.

##### Distribution.

Eastern North America (Thomas 2003).

##### Saproxylic habits.

Little known; adults occur under bark of dead conifers (Thomas 2003), including freshly killed loblolly pine and southern red oak (Gil 2008).

### ﻿Cupedidae

#### ﻿ Tenomerga cinerea

Taxon classificationAnimaliaColeopteraCupedidae

(Say, 1831)

A0B80261-DA4E-5805-A8A9-B0C0A443EEE1

##### Collection information.

USA: Georgia: Clarke Co.: five individuals from five sites. Caught in flight trap from 16 June–14 July 2020.

##### Distribution.

Eastern and central North America.

##### Saproxylic habits.

Larvae bore through rotten oak and pine, and adults occur under bark and in dead wood (Downie and Arnett 1996).

### ﻿Curculionidae

#### ﻿ Acalles carinatus

Taxon classificationAnimaliaColeopteraCurculionidae

LeConte, 1876

8B7EBB05-2510-58BF-BD54-81EFC5DA1B3B

##### Collection information.

USA: Georgia: Clarke Co.: a single individual from one site. Sifted from leaf litter from 27–28 July 2020.

##### Distribution.

Eastern North America.

##### Saproxylic habits.

Emerged from small diameter, dead hardwoods (Ferro et al. 2012a); adults occur under bark of dead trees, such as sugar maple (*Acersaccharum* Marshall (Sapindaceae)) (Blatchley 1925).

#### ﻿ Acalles clavatus

Taxon classificationAnimaliaColeopteraCurculionidae

(Say, 1831)

3557F1DC-4EC7-5D8C-B518-8BC9923998CE

##### Collection information.

USA: **Georgia (new state record)**: Clarke Co.: 28 individuals from 14 sites. Sifted from leaf litter from 29 June–31 August 2020.

##### Distribution.

Southeastern United States.

##### Saproxylic habits.

Emerged from twigs of hardwood trees (Ferro and Nguyen 2016), including southern red oak (Ferro et al. 2009).

##### Conservation.

Significantly associated with, and occurrence probability increases in, old forests (predating 1938 and oak dominated) in the Piedmont (Traylor et al. 2023a, 2024).

#### ﻿ Acamptus rigidus

Taxon classificationAnimaliaColeopteraCurculionidae

LeConte, 1876

B8BD951B-C76C-578C-BD1F-96D4DA177AF8

##### Collection information.

USA: Georgia: Clarke Co.: two individuals from two sites. Caught in flight trap from 2–16 June 2020.

##### Distribution.

Eastern and central North America.

##### Saproxylic habits.

Little known, occur in red-rotten wood (Hanula 1996); larvae of *Acamptus* LeConte, 1876 develop under bark, in dead branches, and in tree wounds (Anderson 1952); species of *Acamptus* are associated with dead wood, tree wounds, and dead portions of living trees, such as rotten tree hollows (Kissinger 1964; Anderson 2002).

#### ﻿ Apteromechus ferratus

Taxon classificationAnimaliaColeopteraCurculionidae

(Say, 1831)

F12C91A8-91C6-5ADC-9624-152EB5FE97EB

##### Collection information.

USA: Georgia: Clarke Co.: 26 individuals from 14 sites. Caught in flight trap from 25 March–26 August 2020.

##### Distribution.

Eastern North America.

##### Saproxylic habits.

Larvae mine the inner bark of recently dead trees, including *Sassafras* Presl (Lauraceae) (Kissinger 1963); emerged from freshly dead hardwood twigs and branches (Ferro et al. 2012a).

##### Conservation.

Significantly higher abundance in secondary (= second-growth) than primary (= old-growth) forests in the southern Appalachian Mountains (Ferro et al. 2012a).

#### ﻿ Apteromechus pumilus

Taxon classificationAnimaliaColeopteraCurculionidae

(Boheman, 1837)

F1DF2501-EC13-50DC-BA3B-9C5EB43E56A5

##### Collection information.

USA: Georgia: Clarke Co.: five individuals from four sites. Caught in flight trap from 25 March–16 June 2020.

##### Distribution.

Southeastern United States (Ciegler 2010).

##### Saproxylic habits.

unknown; larvae of *Apteromechus* Faust, 1896 mine the bark of recently dead trees (Kissinger 1963).

#### ﻿ Cophes fallax

Taxon classificationAnimaliaColeopteraCurculionidae

(LeConte, 1876)

8B388ACA-7FAA-5438-A994-A4896250325A

##### Collection information.

USA: Georgia (new state record*): Clarke Co.: five individuals from four sites. Caught in flight trap and sifted from leaf litter from 2 June–9 September 2020.

##### Distribution.

Eastern North America.

##### Saproxylic habits.

Emerged from a variety of hardwood twigs and branches (Blatchley and Leng 1916; Ferro and Gimmel 2014; Ferro and Nguyen 2016) and associated with fresh, small-diameter wood (Ferro et al. 2012a).

##### Conservation.

Significantly higher abundance in primary (= old-growth) than secondary (= second-growth) forests in the southern Appalachian Mountains (Ferro et al. 2012a).

#### ﻿ Cophes oblongus

Taxon classificationAnimaliaColeopteraCurculionidae

(LeConte, 1876)

8CA3E58A-6C94-5236-85E0-92C6414A6AEB

##### Collection information.

USA: Georgia: Clarke Co.: three individuals from two sites. Caught in flight trap from 27 July–26 August 2020.

##### Distribution.

Southeastern United States and Caribbean islands.

##### Saproxylic habits.

Emerged from hardwood twigs (Ferro and Gimmel 2014; Ferro and Nguyen 2016) and hackberry logs (presumably *Celtis* L. (Cannabaceae)) (Fox 2009).

#### ﻿ Cophes obtentus

Taxon classificationAnimaliaColeopteraCurculionidae

(Herbst, 1797)

DBE20301-E7DC-553A-B974-CF2BBE9244FD

##### Collection information.

USA: Georgia (new state record*): Clarke Co.: two individuals from two sites. Caught in flight trap from 21 April–11 August 2020.

##### Distribution.

Eastern North America into Central America, west to Arizona.

##### Saproxylic habits.

Emerged from hardwood twigs (Ferro et al. 2012a; Ferro and Nguyen 2016); adults occur under bark and in hollow trees (Ciegler 2010).

#### ﻿ Cossonus corticola

Taxon classificationAnimaliaColeopteraCurculionidae

Say, 1831

57A16375-1D27-5C00-BC1B-01B4F7FED14C

##### Collection information.

USA: Georgia: Clarke Co.: two individuals from two sites. Caught in flight trap from 2 June–11 August 2020.

##### Distribution.

Eastern North America to Central America.

##### Saproxylic habits.

Larvae develop, and adults occur in dead pine logs (Anderson 1952; Moser et al. 1971; Gil 2008).

#### ﻿ Cossonus impressifrons

Taxon classificationAnimaliaColeopteraCurculionidae

Boheman, 1838

AF644162-8B8F-5943-AAB4-0CC5BED6B2AA

##### Collection information.

USA: Georgia: Clarke Co.: 25 individuals from 13 sites. Caught in flight trap from 19 May–11 August 2020.

##### Distribution.

Eastern North America.

##### Saproxylic habits.

Occur under bark and in logs of various hardwoods and pine (Blackman and Stage 1924; O’Brien 1997), although it may prefer hardwoods (e.g., oak) to pine (Gil 2008).

#### ﻿ Cryptorhynchus fuscatus

Taxon classificationAnimaliaColeopteraCurculionidae

LeConte, 1876

040E78E7-E4A5-55B6-B917-A724FD49681E

##### Collection information.

USA: Georgia: Clarke Co.: two individuals from two sites. Caught in flight trap from 6 May–26 August 2020.

##### Distribution.

Eastern North America.

##### Saproxylic habits.

Emerged from poison ivy vines and adults occur under bark of dead trees, such as pecan (*Caryaillinoinensis* (Wangenh.) K.Koch) (Anderson 2008a), and in hollow trees (Ciegler 2010).

#### ﻿ Dryophthorus americanus

Taxon classificationAnimaliaColeopteraCurculionidae

Bedel, 1885

980D9817-0CB7-53A7-A3D4-FDC884C1AD97

##### Collection information.

USA: Georgia: Clarke Co.: 11 individuals from nine sites. Caught in flight trap and sifted from leaf litter from 21 April–30 June 2020.

##### Distribution.

Eastern North America.

##### Saproxylic habits.

Consumes decayed woody material and occurs under bark and in rotting wood (Blackman and Stage 1918; Anderson 2002); conifers are listed as primary hosts (including pine and larch (*Larix* Mill. (Pinaceae)) (Blackman and Stage 1918; Blatchley 1928; Ciegler 2010), although it has also emerged in numbers from dead hickory and other hardwoods (Blackman and Stage 1924; Ferro et al. 2012a); highest abundance emerged from logs in intermediate- and late-decay stages (Blackman and Stage 1924; Ulyshen and Hanula 2010; Ferro et al. 2012a, b).

##### Conservation.

Significantly higher abundance in primary (= old-growth) than secondary (= second-growth) forests in the southern Appalachian Mountains (Ferro et al. 2012a).

#### ﻿ Eubulus bisignatus

Taxon classificationAnimaliaColeopteraCurculionidae

(Say, 1831)

AC995D8C-72C1-5074-A4F0-5CDC1431FD31

##### Collection information.

USA: Georgia: Clarke Co.: four individuals from three sites. Caught in flight trap from 25 March–14 July 2020.

##### Distribution.

Eastern North America.

##### Saproxylic habits.

Emerged from dead wood of peach (*Prunuspersica* (L.) Batsch) (Dolphin et al. 1972); adults occur on dead branches and trunks of hardwood trees (Anderson 2008b; Ciegler 2010); larvae likely mine below bark and in the sapwood, as does *Eubulusparochus* (Herbst, 1797) (Halik and Bergdahl 2006).

#### ﻿ Eubulus obliquefasciatus

Taxon classificationAnimaliaColeopteraCurculionidae

(Boheman, 1844)

BD06C39D-EF07-5E43-9957-8EB94118D50F

##### Collection information.

USA: Georgia: Clarke Co.: four individuals from four sites. Caught in flight trap from 25 March–30 June 2020.

##### Distribution.

Eastern United States.

##### Saproxylic habits.

Adults occur on dead trees, such as oak and sweetgum (Anderson 2008b); larvae likely mine below bark and in the sapwood, as does *E. Parochus* (Halik and Bergdahl 2006).

#### ﻿ Eulechriops minuta

Taxon classificationAnimaliaColeopteraCurculionidae

(LeConte, 1824)

B41BDA30-0BE4-569E-981C-22DFFF3922D5

##### Collection information.

USA: Georgia: Clarke Co.: three individuals from two sites. Caught in flight trap from 6 May–14 July 2020.

##### Distribution.

Eastern United States.

##### Saproxylic habits.

unknown, although associated with oaks (Sleeper 1963; Hespenheide 2003a); neotropical *Eulechriops* Faust, 1896 bore into woody materials (Jordal and Kirkendall 1998).

#### ﻿ Himatium errans

Taxon classificationAnimaliaColeopteraCurculionidae

LeConte, 1876

2CC17A4C-E9F9-5670-830B-9D629FF9F78D

##### Collection information.

USA: Georgia: Clarke Co.: four individuals from four sites. Caught in flight trap from 21 April–27 July 2020.

##### Distribution.

Eastern North America.

##### Saproxylic habits.

Occur under bark and within bark beetle galleries in pine (O’Brien 1997; Ciegler 2010); emerged from dead loblolly pine and black walnut (*Juglansnigra* L. (Juglandaceae)) (Ulyshen and Hanula 2009a; Reed et al. 2015); may vector the pathogen of thousand cankers disease (*Geosmithiamorbida* Kolařík, Freeland, Utley and Tisserat (Bionectriaceae)) (Moore et al. 2019).

#### ﻿ Hylobius aliradicis

Taxon classificationAnimaliaColeopteraCurculionidae

Warner, 1966

3D505294-6CFA-5650-82BA-5CFF53AB3B01

##### Collection information.

USA: Georgia: Clarke Co.: a single individual from one site. Caught in flight trap from 6–19 May 2020.

##### Distribution.

Southeastern United States.

##### Saproxylic habits.

Larvae develop in the roots of young, dying slash pines (*Pinuselliottii* Engelm.), and have been collected from pine bolts (Warner 1966); although Warner (1966) suggests that the larvae were the primary cause of tree death, more recent work suggests that *H. Aliradicis* and related pine weevils principally colonize already stressed trees (Helbig et al. 2016).

#### ﻿ Hylocurus harnedi

Taxon classificationAnimaliaColeopteraCurculionidae

(Blackman, 1920)

70D30BA3-DBF5-57FC-A771-80E42C4B0912

##### Collection information.

USA: **Georgia (new state record)**: Clarke Co.: one individual from one site. Caught in flight trap from 25 March–9 April 2020.

##### Distribution.

Southeastern United States (Wood 1982).

##### Saproxylic habits.

Larvae are xylophagous within dead branches of hickory (Wood 1982).

#### ﻿ Laemosaccus obrieni

Taxon classificationAnimaliaColeopteraCurculionidae

Hespenheide, 2019

61C415EA-29D3-5B3F-9138-A571282DF33B

##### Collection information.

USA: Georgia: Clarke Co.: two individuals from one site. Caught in flight trap from 21 April–6 May 2020.

##### Distribution.

Eastern North America (Hespenheide 2019).

##### Saproxylic habits.

Bores into twigs and stems, and has emerged from various oaks, hickories, and American chestnut (*Castaneadentata* (Marshall) Borkh. (Fagaceae)) (Hespenheide 2019).

#### ﻿ Lechriops oculata

Taxon classificationAnimaliaColeopteraCurculionidae

(Say, 1824)

4C8EA53F-3E7C-56B2-AAE4-728C28BC8E20

##### Collection information.

USA: Georgia: Clarke Co.: 838 individuals from 36 sites. Caught in flight trap from 9 March–9 September 2020.

##### Distribution.

Eastern North America.

##### Saproxylic habits.

Adults occur under bark (Ott 2011a); western species of *Lechriops* develop under the bark of conifers (Hespenheide 2003b), although *L. оculata* is associated with hardwood trees (Sleeper 1963).

#### ﻿ Magdalis barbita

Taxon classificationAnimaliaColeopteraCurculionidae

(Say, 1831)

F7680C5C-AF6B-57A5-B155-AB5871773004

##### Collection information.

USA: Georgia: Clarke Co.: four individuals from four sites. Caught in flight trap from 9 April–6 May 2020.

##### Distribution.

Eastern North America.

##### Saproxylic habits.

Larvae feed on the inner bark and sapwood of unhealthy and dying elms (*Ulmus* L. (Ulmaceae)) (Baker 1972); emerged from slippery elm (*Ulmusrubra* Muhl.) and American elm (*Ulmusamericana* L.) (Webster et al. 2012a; Haack 2020); adults also occur under bark of recently dead hardwoods, such as oak and hickory (Blatchley and Leng 1916).

#### ﻿ Magdalis perforata

Taxon classificationAnimaliaColeopteraCurculionidae

Horn, 1873

605ED236-49EC-5C1D-B816-DB26B0C5BF53

##### Collection information.

USA: Georgia: Clarke Co.: two individuals from two sites. Caught in flight trap from 21 April–26 August 2020.

##### Distribution.

Eastern North America.

##### Saproxylic habits.

Emerged from loblolly pine (Helbig et al. 2016); larvae likely feed on the inner bark and sapwood as do other species of *Magdalis* Germar, 1817 (Baker 1972).

#### ﻿ Micromimus corticalis

Taxon classificationAnimaliaColeopteraCurculionidae

(Boheman, 1845)

72D8816D-4D0B-57A9-9AD2-A7CEA062A037

##### Collection information.

USA: **Georgia (new state record)**: Clarke Co.: 19 individuals from six sites. Caught in flight trap from 21 April–14 July 2020.

##### Distribution.

Southeastern United States (Ciegler 2010).

##### Saproxylic habits.

Emerged from hardwood woody debris (Ferro et al. 2012a); adults occur under bark (Ciegler 2010).

#### ﻿ Phaenomerus foveipennis

Taxon classificationAnimaliaColeopteraCurculionidae

(Morimoto, 1961)

930ADFE9-5A3B-5427-8890-6EB146342234

##### Collection information.

USA: Georgia: Clarke Co.: a single individual from one site. Caught in flight trap from 2–16 June 2020.

##### Distribution.

Eastern Palearctic, adventive in the southeastern United States (Schnepp and Anderson 2021).

##### Saproxylic habits.

Occurs under bark in its native range (Morimoto 1961); other species of *Phaenomerus* Schönherr, 1836 are found in association with ambrosia beetles or have emerged from wood packaging (Thompson 1996; Schnepp and Anderson 2021).

#### ﻿ Pissodes nemorensis

Taxon classificationAnimaliaColeopteraCurculionidae

Germar, 1824

9FA30A25-2050-57DA-9991-00CB20370082

##### Collection information.

USA: Georgia: Clarke Co.: four individuals from three sites. Caught in flight trap from 9 March–2 June 2020.

##### Distribution.

Eastern North America.

##### Saproxylic habits.

Larvae feed in the phloem and sapwood of weakened or freshly killed pine (Atkinson et al. 1988); while generally considered a pest of pines (e.g., Ollieu 1971), more recent work suggests *P. Nemorensis* and related pine weevils principally colonize already stressed trees (Helbig et al. 2016).

#### ﻿ Plocamus echidna

Taxon classificationAnimaliaColeopteraCurculionidae

(LeConte, 1876)

39219FFF-E965-515D-986C-A80AEBFBF703

##### Collection information.

USA: **Georgia (new state record)**: Clarke Co.: two individuals from two sites. Caught in flight trap from 19 May–2 June 2020.

##### Distribution.

Eastern North America into Central America.

##### Saproxylic habits.

Adults occur on dead beech (*Fagusgrandifolia* Ehrh. (Fagaceae)) (Beutenmuller 1893; de Tonnancour et al. 2017) and may consume hickory as well (Blatchley and Leng 1916).

#### ﻿ Plocamus hispidulus

Taxon classificationAnimaliaColeopteraCurculionidae

LeConte, 1876

AB198365-FF27-569E-AB34-A26C212FF56B

##### Collection information.

USA: Georgia: Clarke Co.: two individuals from two sites. Caught in flight trap from 25 March–11 August 2020.

##### Distribution.

Eastern United States.

##### Saproxylic habits.

Breeds in dead branches of “locust” trees (presumably *Robinia* L. оr *Gleditsia* L. (both in Fabaceae)) (Beutenmuller 1893), and also emerged from black walnut (Reed et al. 2015).

#### ﻿ Pseudopentarthrum simplex

Taxon classificationAnimaliaColeopteraCurculionidae

Casey, 1892

37A1F6C3-31C9-5A67-8F7B-79CCBE972405

##### Collection information.

USA: Georgia (new state record*): Clarke Co.: a single individual from one site. Caught in flight trap from 16–30 June 2020.

##### Distribution.

Eastern North America.

##### Saproxylic habits.

Reared from a wound in a maple tree (*Acer* L.) and adults occur on dead trees and shrubs, such as wax myrtle (*Morellacerifera* (L.) Small (Myricaceae)) (Ciegler 2010); species of *Pseudopentarthrum* Wollaston, 1873 are associated with dead wood and various microhabitats (e.g., tree hollows; Anderson 2002).

#### ﻿ Rhyncolus discors

Taxon classificationAnimaliaColeopteraCurculionidae

Casey, 1892

42D7CE5E-DC03-5C20-815C-58AAC17A04A9

##### Collection information.

USA: Georgia: Clarke Co.: 11 individuals from eight sites. Caught in flight trap from 25 March–2 June 2020.

##### Distribution.

Eastern United States.

##### Saproxylic habits.

Adults occur under bark and in wood of pine snags (Howden and Vogt 1951); larvae of *Rhyncolus* Germar, 1817 develop in dead wood (Anderson 1952).

#### ﻿ Stenomimus pallidus

Taxon classificationAnimaliaColeopteraCurculionidae

(Boheman, 1845)

4DF0BCC3-F452-5479-AAFC-B82EF2C86A4D

##### Collection information.

USA: **Georgia (new state record)**: Clarke Co.: two individuals from one site. Caught in a flight trap from 21 April–6 May 2020.

##### Distribution.

Eastern North America.

##### Saproxylic habits.

Larvae develop under the bark of wounded and dead oak, hickory, and black walnut (Anderson 1952; Ciegler 2010); known to vector the pathogen of thousand cankers disease (*G.morbida*, Juzwik et al. 2015).

#### ﻿ Stenoscelis andersoni

Taxon classificationAnimaliaColeopteraCurculionidae

Buchanan, 1948

77AEE66C-B497-5A18-8AA1-B31408AFD188

##### Collection information.

USA: Georgia: Clarke Co.: 19 individuals from 16 sites. Caught in flight trap from 21 April–16 June 2020.

##### Distribution.

Eastern United States.

##### Saproxylic habits.

Larvae develop in rotting birch (*Betula* L. (Betulaceae)) (Anderson 1952) and adults occur in soft dead wood (Kissinger 1955); emerged from burned and unburned loblolly pine logs (Ulyshen et al. 2010); associated with snags in bottomland forests (Ulyshen and Hanula 2009a).

#### ﻿ Stenoscelis brevis

Taxon classificationAnimaliaColeopteraCurculionidae

(Boheman, 1845)

2815CFE9-0877-597F-81F4-3F944296B678

##### Collection information.

USA: Georgia: Clarke Co.: 19 individuals from 10 sites. Caught in flight trap from 6 May–16 June 2020.

##### Distribution.

Eastern North America.

##### Saproxylic habits.

Larvae develop, and adults occur gregariously in decaying wood of many hardwoods and larch, including dead portions and exposed wood in living trees (Blackman and Stage 1918, 1924; Anderson 1952); adults also occur in solid, dry wood and old stumps (Beutenmuller 1893; Hoffmann 1942); associated with moderately decayed wood (Ferro et al. 2012a).

##### Conservation.

Significantly higher abundance in secondary (= second-growth) than primary (= old-growth) forests in the southern Appalachian Mountains (Ferro et al. 2012a); rare in the Maritime Provinces of Canada, possibly due to the history of intensive forest management in the region altering forest composition and structure (Majka 2007).

#### ﻿ Tomolips quercicola

Taxon classificationAnimaliaColeopteraCurculionidae

(Boheman, 1845)

9693B865-BBA3-5479-B115-A38F9B2BC70A

##### Collection information.

USA: Georgia: Clarke Co.: eight individuals from eight sites. Caught in flight trap from 21 April–30 June 2020.

##### Distribution.

Eastern North America south to Central America.

##### Saproxylic habits.

Emerged from loblolly pine, water oak, and sweetgum in bottomland forests (Ulyshen and Hanula 2009a); adults occur in rotten deadwood, tree hollows, and under bark of various trees, including beech and magnolia (*Magnolia* L. (Magnoliaceae)) (Blatchley 1928; Kissinger 1955; Ciegler 2010).

#### ﻿ Xyleborus pfeilii

Taxon classificationAnimaliaColeopteraCurculionidae

(Ratzeburg, 1837)

E6702CA5-0178-5B57-AF47-80B598922F8A

##### Collection information.

USA: **Georgia (new state record)**: Clarke Co.: two individuals from two sites. Caught in flight trap from 30 June–14 July 2020.

##### Distribution.

Introduced across the world, adventive in North America (Gomez et al. 2018).

##### Saproxylic habits.

Larvae are ambrosial feeders and polyphagous in tree-hosts (Wood 1982; Wood and Bright 1992).

### ﻿Dermestidae

#### ﻿ Orphilus ater

Taxon classificationAnimaliaColeopteraDermestidae

Erichson, 1846

57B68CAF-B3B5-5DD6-9174-581A3FCF6E06

##### Collection information.

USA: Georgia: Clarke Co.: 27 individuals from 15 sites. Caught in flight trap from 9 March–16 June 2020.

##### Distribution.

Eastern North America.

##### Saproxylic habits.

unknown; larvae of *Orphilus* Erichson, 1846 develop within dry, fungus-infested branches (Beal 1985; Pushkin and Mykhailovych 2019).

##### Conservation.

Occurrence probability increases in old forests (predating 1938 and oak dominated) in the Piedmont (Traylor et al. 2024).

### ﻿Disteniidae

#### ﻿ Elytrimitatrix undata

Taxon classificationAnimaliaColeopteraDisteniidae

(Fabricius, 1775)

203A7584-67CE-59A5-8B46-3CFE425BD608

##### Collection information.

USA: Georgia: Clarke Co.: four individuals from three sites. Caught in flight trap from 14–27 July 2020.

##### Distribution.

Eastern and central United States.

##### Saproxylic habits.

Larvae develop within a variety of hardwoods and pine (Lingafelter 2007).

### ﻿Elateridae

#### ﻿ Alaus myops

Taxon classificationAnimaliaColeopteraElateridae

(Fabricius, 1801)

D9BD67FB-280F-597C-ADEF-0475FA44B264

##### Collection information.

USA: Georgia: Clarke Co.: a single individual from one site. Caught in flight trap from 2–16 June 2020.

##### Distribution.

Eastern North America.

##### Saproxylic habits.

Larvae are predators of woodboring insects in dead pine logs and stumps (Frost 1916; Savely 1939); occupies logs throughout decomposition (Savely 1939; Ulyshen and Hanula 2010).

#### ﻿Genus Ampedus

Taxon classificationAnimaliaColeopteraElateridae

Dejean, 1833

42622590-357F-56D6-8B29-D5F4574A8D16

##### Collection information.

See inidivudal species below.

##### Saproxylic habits.

Little is known about specific habits of each species, but larvae of *Ampedus* develop in decomposing wood where they are thought to prey on other invertebrates (Ramberg 1979).

#### ﻿ Ampedus areolatus

Taxon classificationAnimaliaColeopteraElateridae

(Say, 1823)

4FCBF36E-59E8-5C38-A79D-046ADE83ECC0

##### Collection information.

USA: Georgia: Clarke Co.: nine individuals from eight sites. Caught in flight trap from 9 April–14 July 2020.

##### Distribution.

Eastern and central North America.

##### Saproxylic habits.

Emerged from loblolly pine logs throughout decomposition (Ulyshen and Hanula 2010); emerged from dead hardwoods and associated with larger diameter, moderately decayed pieces (Ferro et al. 2012a); adults occur under bark (Mathison 2021).

##### Conservation.

Significantly higher abundance in primary (= old-growth) than secondary (= second-growth) forests in the southern Appalachian Mountains (Ferro et al. 2012a).

#### ﻿ Ampedus collaris

Taxon classificationAnimaliaColeopteraElateridae

(Say, 1825)

AD722D3E-C24F-5AEF-9AD6-5744CA07EE9D

##### Collection information.

USA: Georgia: Clarke Co.: 84 individuals from 21 sites. Caught in flight trap from 9 March–30 June 2020.

##### Distribution.

Eastern North America.

##### Saproxylic habits.

Emerged from white spruce (*Piceaglauca* (Moench) Voss (Pinaceae)) (Majka and Johnson 2008); adults occur in rotting wood, including hickory, white spruce, and eastern white pine (*Pinusstrobus* L.) (Ramberg 1979).

##### Conservation.

Significantly associated with young forests (regrown since 1938 and pine dominated) in the Piedmont (Traylor et al. 2023a).

#### ﻿ Ampedus fuscatus

Taxon classificationAnimaliaColeopteraElateridae

(Melsheimer, 1845)

8EC459B3-4048-50DA-B6C5-5DA791BB2C39

##### Collection information.

USA: Georgia: Clarke Co.: six individuals from six sites. Caught in flight trap from 25 March–27 July 2020.

##### Distribution.

Eastern North America.

##### Saproxylic habits.

Adults occur in rotting wood and under bark, including hickory, cypress (*Cupressus* L. (Cupressaceae)), American sycamore (*Platanusoccidentalis* L. (Platanaceae)), pine, and blackjack oak (*Quercusmarilandica* (Münchh.)) (Ramberg 1979; Mathison 2021).

##### Conservation.

Significantly associated with young forests (regrown since 1938 and pine dominated) in highly forested landscapes (> 50% forest) in the Piedmont (Traylor et al. 2023a).

#### ﻿ Ampedus melanotoides

Taxon classificationAnimaliaColeopteraElateridae

Brown, 1933

378FA2BE-31BC-571A-A19A-813B2105DF9A

##### Collection information.

USA: Georgia: Clarke Co.: 74 individuals from 26 sites. Caught in flight trap from 9 March–9 September 2020.

##### Distribution.

Eastern North America.

##### Saproxylic habits.

Adults occur in rotting wood, including hickory and willow (Ramberg 1979).

#### ﻿ Ampedus melsheimeri

Taxon classificationAnimaliaColeopteraElateridae

(Leng, 1918)

F0351CBD-57FB-5D1E-968C-8F5B6AD937DF

##### Collection information.

USA: Georgia: Clarke Co.: six individuals from four sites. Caught in flight trap from 21 April–11 August 2020.

##### Distribution.

Eastern North America.

##### Saproxylic habits.

Unknown.

#### ﻿ Ampedus militaris

Taxon classificationAnimaliaColeopteraElateridae

(Harris, 1836)

7CE38103-9646-53DE-931D-2409C28D9003

##### Collection information.

USA: Georgia: Clarke Co.: a single individual from one site. Caught in flight trap from 21 April–6 May 2020.

##### Distribution.

Eastern North America.

##### Saproxylic habits.

Little known, but adults occur under bark (Kelly 2011) and under decomposing logs (Webb 2019).

#### ﻿ Ampedus nigricollis

Taxon classificationAnimaliaColeopteraElateridae

(Herbst, 1801)

7EFC4593-E97C-5E0C-97C6-CF2E5D6B638A

##### Collection information.

USA: Georgia: Clarke Co.: four individuals from four sites. Caught in flight trap from 9 March–2 June 2020.

##### Distribution.

Eastern North America.

##### Saproxylic habits.

Adults occur under bark and in rotting wood, including pine, hemlock (*Tsuga* (Endl.) Carrière (Pinaceae)), and a variety of hardwoods (Ramberg 1979; Mathison 2021).

#### ﻿ Ampedus pedalis

Taxon classificationAnimaliaColeopteraElateridae

(Germar, 1843)

D18FEE48-50DD-562C-B42A-B604594461FC

##### Collection information.

USA: Georgia: Clarke Co.: 149 individuals from 27 sites. Caught in flight trap from 25 March–30 June 2020.

##### Distribution.

Eastern North America.

##### Saproxylic habits.

Adults occur in rotting wood, such as elm (Ramberg 1979).

#### ﻿ Ampedus pusio

Taxon classificationAnimaliaColeopteraElateridae

Germar, 1844

66A1458A-FE39-532D-B605-D9558B677795

##### Collection information.

USA: Georgia: Clarke Co.: 102 individuals from 30 sites. Caught in flight trap from 16 June–9 September 2020.

##### Distribution.

Eastern United States.

##### Saproxylic habits.

Adults occur in snags and rotten stumps of pine (Ramberg 1979; Mathison 2021).

#### ﻿ Ampedus rubricollis

Taxon classificationAnimaliaColeopteraElateridae

(Herbst, 1806)

213A4F59-C82E-5BA4-A04F-E37E1A531FC4

##### Collection information.

USA: Georgia: Clarke Co.: four individuals from four sites. Caught in flight trap from 9 March–19 May 2020.

##### Distribution.

Eastern North America.

##### Saproxylic habits.

Adults occur under bark of dead trees, such as pine (Ramberg 1979; Mathison 2021).

#### ﻿ Ampedus sanguinipennis

Taxon classificationAnimaliaColeopteraElateridae

(Say, 1823)

557F1EB3-ADDD-57D4-9CBE-F7A2328AE2F7

##### Collection information.

USA: Georgia: Clarke Co.: seven individuals from one site. Caught in flight trap from 19 May–30 June 2020.

##### Distribution.

Eastern and central North America.

##### Saproxylic habits.

Adults occur in rotting wood and under bark, including cypress, pine, oak, willow, and pear (*Pyrus* L. (Rosaceae)) (Ramberg 1979; Mathison 2021).

#### ﻿ Athous cucullatus

Taxon classificationAnimaliaColeopteraElateridae

(Say, 1825)

08BE145C-E36B-5D96-B976-1E68257A89A3

##### Collection information.

USA: Georgia: Clarke Co.: 24 individuals from 19 sites. Caught in flight trap from 16 June–9 September 2020.

##### Distribution.

Eastern North America.

##### Saproxylic habits.

Larvae occur in dead wood where they prey upon woodboring larvae (Kirk 1922; Glen 1950); emerged from decayed loblolly pine, hickory, elm, and various hardwoods (Blackman and Stage 1924; Hoffmann 1942; Ulyshen and Hanula 2010; Ferro et al. 2012a).

##### Conservation.

Occurrence probability increases in old forests (predating 1938 and oak dominated) in the Piedmont (Traylor et al. 2024).

#### ﻿ Dipropus soleatus

Taxon classificationAnimaliaColeopteraElateridae

(Say, 1839)

28E00CDF-5480-555A-9C91-FA44D2FD2B99

##### Collection information.

USA: Georgia: Clarke Co.: 11 individuals from nine sites. Caught in flight trap from 30 June–26 August 2020.

##### Distribution.

Eastern United States (Mathison 2021).

##### Saproxylic habits.

Larvae develop in soft decaying wood, including maple and cherry (Jewett 1946).

##### Conservation.

Occurrence probability increases in old forests (predating 1938 and oak dominated) in the Piedmont (Traylor et al. 2024).

#### ﻿ Drapetes exstriatus

Taxon classificationAnimaliaColeopteraElateridae

(Say, 1834)

5A39316C-2373-5C0C-B8CF-DB73BCDE6674

##### Collection information.

USA: Georgia: Clarke Co.: two individuals from one site. Caught in flight trap from 30 June–11 August 2020.

##### Distribution.

Eastern United States.

##### Saproxylic habits.

Adults occur under bark, including oak and a long-dead fallen tree (Wilson 2012; Mathison 2021); larvae of *Drapetes* (Dejean, 1821) develop under loose bark or in decaying wood (Johnson 2015).

#### ﻿ Drapetes quadripustulatus

Taxon classificationAnimaliaColeopteraElateridae

Bonvouloir, 1859

2C4FF38B-8CDD-5D8D-91DB-5E853E6C1AF7

##### Collection information.

USA: Georgia: Clarke Co.: a single individual from one site. Caught in flight trap from 26 August–9 September 2020.

##### Distribution.

Eastern United States (Mathison 2021).

##### Saproxylic habits.

Emerged from loblolly pine throughout decomposition and in both burned and unburned logs (Ulyshen and Hanula 2010; Ulyshen et al. 2010), and also occur on southern red oak logs (Gil 2008); larvae of *Drapetes* develop under loose bark or in decaying wood (Johnson 2015).

#### ﻿ Lacon avitus

Taxon classificationAnimaliaColeopteraElateridae

(Say, 1839)

98D8BA39-C808-5ECE-980B-213B3A0A6E2A

##### Collection information.

USA: Georgia: Clarke Co.: a single individual from one site. Caught in flight trap from 14–27 July 2020.

##### Distribution.

Eastern United States.

##### Saproxylic habits.

unknown; species of *Lacon* Laporte, 1838 commonly occur under bark (Mathison 2021).

#### ﻿ Lacon discoideus

Taxon classificationAnimaliaColeopteraElateridae

(Weber, 1801)

442DC97E-A33F-5A36-B421-321265662BD6

##### Collection information.

USA: Georgia: Clarke Co.: 18 individuals from nine sites. Caught in flight trap from 25 March–26 August 2020.

##### Distribution.

Eastern North America.

##### Saproxylic habits.

Adults occur in rotting logs; under bark (pine, magnolia, and oak), and within tree-hollow litter (Mathison 2021).

#### ﻿ Lacon marmoratus

Taxon classificationAnimaliaColeopteraElateridae

(Fabricius, 1801)

B3FEEB16-F92B-5B13-A923-9CDF0ED0A038

##### Collection information.

USA: Georgia: Clarke Co.: three individuals from two sites. Caught in flight trap from 21 April–9 September 2020.

##### Distribution.

Eastern North America.

##### Saproxylic habits.

Adults occur under bark of decayed oak and pine trunks (Dozier 1918).

#### ﻿ Orthostethus infuscatus

Taxon classificationAnimaliaColeopteraElateridae

(Germar, 1844)

F433FE45-0DEE-5DBC-AF4B-DC32E570B504

##### Collection information.

USA: Georgia: Clarke Co.: 173 individuals from 18 sites. Caught in flight trap from 2 June–9 September 2020.

##### Distribution.

Eastern North America, west to Arizona, south to South America.

##### Saproxylic habits.

Larvae inhabit decaying logs (Dozier 1918), apparently feeding on rotting wood (Savely 1939); emerged from decayed loblolly pine and oak (Savely 1939; Ulyshen and Hanula 2010); adults have been found within decayed interiors of living trees (Hepting 1935).

##### Conservation.

Occurrence probability increases in old forests (predating 1938 and oak dominated) in the Piedmont (Traylor et al. 2024).

#### ﻿ Parallelostethus attenuatus

Taxon classificationAnimaliaColeopteraElateridae

(Say, 1825)

2DE33777-2CD0-5DA8-860D-5DDE9FC0C09B

##### Collection information.

USA: Georgia: Clarke Co.: two individuals from two sites. Caught in flight trap from 30 June–27 July 2020.

##### Distribution.

Eastern North America.

##### Saproxylic habits.

Occurs in decayed wood, stumps, and tree-hollows of maple, oak, hackberry, and sycamore (*Platanus* L.) (Jewett 1946); feeds on rotten wood material (Kirk 1922).

### ﻿Endecatomidae

#### ﻿ Endecatomus rugosus

Taxon classificationAnimaliaColeopteraEndecatomidae

(Randall, 1838)

D80070DA-AA6D-5E0A-A824-9D2017854350

##### Collection information.

USA: Georgia: Clarke Co.: six individuals from two sites. Caught in flight trap from 9 March–9 April 2020.

##### Distribution.

Eastern North America.

##### Saproxylic habits.

Larvae develop within the shelf fungus *Fuscoporiagilva* (Schwein.) T. Wagner and M. Fisch. (Hymenochaetaceae) (Fisher 1950; Crowson 1961); also emerged from living polypores of *Fomesfomentarius* (L.) Fr. (Polyporaceae) (Matthewman and Pielou 1971).

### ﻿Endomychidae

#### ﻿ Stenotarsus hispidus

Taxon classificationAnimaliaColeopteraEndomychidae

(Herbst, 1799)

B544C22D-59AD-5985-92EB-0D8D18A41ECE

##### Collection information.

USA: Georgia: Clarke Co.: a single individual from one site. Caught in flight trap from 21 April–6 May 2020.

##### Distribution.

Eastern United States.

##### Saproxylic habits.

Little known, adults occur under bark (Ott 2011b); adults and larvae are likely mycophagous on larger basidiomycete fungi (Skelley and Leschen 2002); adults of some *Stenotarsus* Perty, 1832 aggregate on their host fungi (Shockley et al. 2009).

### ﻿Erotylidae

#### ﻿ Dacne quadrimaculata

Taxon classificationAnimaliaColeopteraErotylidae

(Say, 1835)

E16B701A-63DF-57E6-B4BB-67B73757A1D7

##### Collection information.

USA: Georgia: Clarke Co.: 21 individuals from 13 sites. Caught in flight trap from 9 March–6 May 2020.

##### Distribution.

Eastern North America.

##### Saproxylic habits.

Larvae develop, and adults occur in a variety of polypore fungi, but primarily dryad’s saddle (*Cerioporussquamosus* (Huds.) Quél. (Polyporaceae)) and oyster mushroom (*Pleurotus* (Fr.) P. Kumm. (Pleurotaceae)) (Skelley et al. 1991).

##### Conservation.

Occurrence probability increases with the amount of landscape forest cover in the Piedmont (Traylor et al. 2024).

#### ﻿ Ischyrus quadripunctatus

Taxon classificationAnimaliaColeopteraErotylidae

(Olivier, 1791)

3503C99A-8984-5C2B-A31D-365904C9B976

##### Collection information.

USA: Georgia: Clarke Co.: two individuals from two sites. Caught in flight trap from 25 March–26 August 2020.

##### Distribution.

Eastern North America, west to Arizona, south to South America.

##### Saproxylic habits.

Emerged from *Irpexlatemarginatus* (Durieu and Mont.) C.C. Chen and Sheng H. Wu, 2021 (Irpicaceae), and adults feed on other soft polypores (Skelley et al. 1991; Goodrich and Springer 1999).

#### ﻿ Megalodacne fasciata

Taxon classificationAnimaliaColeopteraErotylidae

(Fabricius, 1777)

08B750CB-32E0-5E0E-88B7-636771230633

##### Collection information.

USA: Georgia: Clarke Co.: a single individual from one site. Caught in flight trap from 26 August–9 September 2020.

##### Distribution.

Eastern North America, likely introduced in the western United States (Goodrich and Springer 1999).

##### Saproxylic habits.

Larvae develop within polypores of *Ganodermalucidum* (Curtis) P. Karst. (Ganodermataceae), and adults feed on a wider range of polypores (Skelley et al. 1991).

#### ﻿ Triplax festiva

Taxon classificationAnimaliaColeopteraErotylidae

Lacordaire, 1842

99993178-894C-5D5F-AB75-7198510FA865

##### Collection information.

USA: Georgia: Clarke Co.: three individuals from two sites. Caught in flight trap from 9–25 March 2020.

##### Distribution.

Eastern North America.

##### Saproxylic habits.

Larvae develop within polypores of *Inonotus* P. Karst. (Hymenochaetaceae) (Skelley et al. 1991).

#### ﻿ Triplax frontalis

Taxon classificationAnimaliaColeopteraErotylidae

Horn, 1862

F97E088C-2EA8-5EE2-8168-2429B713A448

##### Collection information.

USA: Georgia: Clarke Co.: three individuals from two sites. Caught in flight trap from 9 March–9 April 2020.

##### Distribution.

Eastern and central United States.

##### Saproxylic habits.

Larvae develop within *Xanthoporiaandersonii* (Ellis and Everh.) Murrill (Hymenochaetaceae), and adults occur on species of *Inonotus* as well (Skelley et al. 1991).

#### ﻿ Tritoma atriventris

Taxon classificationAnimaliaColeopteraErotylidae

LeConte, 1847

A7D83435-E97D-5D95-8038-21ECA7374A4F

##### Collection information.

USA: Georgia: Clarke Co.: two individuals from two sites. Caught in flight trap and sifted from leaf litter from 2 June–31 August 2020.

##### Distribution.

Southeastern and south-central United States (Goodrich and Springer 1999).

##### Saproxylic habits.

Larvae develop in a variety of fungi growing on dead and dying wood, including *Desarmillariatabescens* (Scop.) R.A. Koch and Aime (Physalacriaceae), *Lentinusarcularius* (Batsch) Zmitr. (Polyporaceae), *Omphalotusilludens* (Schwein.) Bresinsky and Besl (Omphalotaceae), and *Pluteus* Fr. (Plutaceae), and adults occur on numerous other fungi (Skelley et al. 1991).

#### ﻿ Tritoma sanguinipennis

Taxon classificationAnimaliaColeopteraErotylidae

(Say, 1825)

1E800C5B-20FB-519E-8184-B99A81B43891

##### Collection information.

USA: Georgia: Clarke Co.: four individuals from three sites. Caught in flight trap from 21 April–30 June 2020.

##### Distribution.

Eastern North America.

##### Saproxylic habits.

Larvae develop in *Lentinusarcularius*, and adults occur on several other fungi (Skelley et al. 1991).

### ﻿Eucinetidae

#### ﻿ Eucinetus strigosus

Taxon classificationAnimaliaColeopteraEucinetidae

LeConte, 1875

3DF7B50E-4AAB-53EE-8FA9-C67848F47D58

##### Collection information.

USA: Georgia: Clarke Co.: a single individual from one site. Sifted from leaf litter from 27–28 July 2020.

##### Distribution.

Eastern United States.

##### Saproxylic habits.

unknown; species of *Eucinetus* Germar, 1818 breed in slime molds and basidiomycete fungi growing on dead wood, and often occur under bark or in decaying wood (Wheeler and Hoebeke 1984).

### ﻿Eucnemidae

#### ﻿ Adelothyreus dejeani

Taxon classificationAnimaliaColeopteraEucnemidae

Bonvouloir, 1872

88CFA0BD-ECD4-555D-893C-9B87CD0238D8

##### Collection information.

USA: Georgia: Clarke Co.: a single individual from one site. Caught in flight trap from 19 May–2 June 2020.

##### Distribution.

Southeastern United States (Otto and Karns 2017).

##### Saproxylic habits.

Emerged from hardwood twigs (Ferro and Nguyen 2016).

#### ﻿ Deltometopus amoenicornis

Taxon classificationAnimaliaColeopteraEucnemidae

(Say, 1836)

65329094-AD0A-5860-92AC-A944673048A5

##### Collection information.

USA: Georgia: Clarke Co.: a single individual from one site. Caught in flight trap from 30 June–14 July 2020.

##### Distribution.

Eastern North America.

##### Saproxylic habits.

Larvae develop in white-rotted or otherwise unspecified decayed wood, including maple, hickory, American beech, and possibly conifers (Knull 1947; Muona 2000; Otto 2012a).

#### ﻿ Dirrhagofarsus lewisi

Taxon classificationAnimaliaColeopteraEucnemidae

(Fleutiaux, 1900)

5E509D8E-B582-50A4-A493-66A9D3FAEFA5

##### Collection information.

USA: Georgia: Clarke Co.: 104 individuals from 32 sites. Caught in flight trap from 25 March–9 September 2020.

##### Distribution.

Native to Japan, adventive in the eastern United States (Ford and Spilman 1979; Otto and Karns 2017).

##### Saproxylic habits.

Larvae develop in the wet sapwood of American beech (Ford and Spilman 1979); emerged from moderately decayed hardwood logs (Ferro et al. 2012a) and occur on southern red oak (Gil 2008).

#### ﻿ Dromaeolus badius

Taxon classificationAnimaliaColeopteraEucnemidae

(Melsheimer, 1844)

4C5D23AE-7938-58E8-8C6F-99F9E204609A

##### Collection information.

USA: Georgia: Clarke Co.: five individuals from four sites. Caught in flight trap from 16 June–27 July 2020.

##### Distribution.

Eastern North America, west to Arizona, Utah, and Idaho (Muona 2000).

##### Saproxylic habits.

Larvae develop in decayed oak stumps (Osten Sacken 1862) and have also emerged from decayed tulip poplar (*Liriodendrontulipifera* L. (Magnoliaceae)), hickory, and aspen (*Populus* L. (Saliceae)аrn 1909; Kirk 1922; Muona 2000).

#### ﻿ Dromaeolus turnbowi

Taxon classificationAnimaliaColeopteraEucnemidae

Muona, 2000

06E2708E-9D57-57B0-9F74-7D3D8CD5370C

##### Collection information.

USA: Georgia: Clarke Co.: a single individual from one site. Caught in flight trap from 2–16 June 2020.

##### Distribution.

Eastern United States (Otto 2022).

##### Saproxylic habits.

unknown; species of *Dromaeolus* Kiesenwetter, 1858 develop within decaying wood (Muona 2000).

#### ﻿ Entomophthalmus rufiolus

Taxon classificationAnimaliaColeopteraEucnemidae

(LeConte, 1866)

9C80636F-605D-51FA-822C-52B5850C4F8C

##### Collection information.

USA: Georgia: Clarke Co.: 12 individuals from eight sites. Caught in flight trap from 19 May–9 September 2020.

##### Distribution.

Eastern North America.

##### Saproxylic habits.

Larvae develop within decayed, white-rotted hardwoods, including oak, maple, and basswood (*Tilia* L. (Malvaceae)), usually in sections of logs with little moisture (Ferro et al. 2012a; Otto 2014).

#### ﻿ Hylis terminalis

Taxon classificationAnimaliaColeopteraEucnemidae

(LeConte, 1866)

4D11995E-4438-51F7-AE9B-A25F2BD6DC49

##### Collection information.

USA: Georgia: Clarke Co.: 11 individuals from eight sites. Caught in flight trap from 21 April–16 June 2020.

##### Distribution.

Eastern North America.

##### Saproxylic habits.

Emerged from hickory limbs and a moist, decayed American beech log (Horn 1886; Knull 1946; Muona 2000)

#### ﻿ Isorhipis obliqua

Taxon classificationAnimaliaColeopteraEucnemidae

(Say, 1836)

F7F8A741-C6D3-54B1-9A7E-FFE445AE0ECD

##### Collection information.

USA: Georgia: Clarke Co.: 866 individuals from 38 sites. Caught in flight trap from 21 April–11 August 2020.

##### Distribution.

Eastern North America.

##### Saproxylic habits.

Larvae develop in the heartwood of decayed hardwoods (e.g., maple; Peterson 1960), and have emerged from dry, dead wood of various hardwoods (Knull 1946; Muona 2000); associated with moderately decayed logs (Ferro et al. 2012a).

##### Conservation.

Significantly higher abundance in secondary (= second-growth) than primary (= old-growth) forests in the southern Appalachian Mountains (Ferro et al. 2012a); significantly associated with old forests (predating 1938 and oak dominated) in the Piedmont (Traylor et al. 2023a).

#### ﻿ Melasis pectinicornis

Taxon classificationAnimaliaColeopteraEucnemidae

Melsheimer, 1844

25B5A4BE-BE79-5DA0-82F0-A4DD1F4C396C

##### Collection information.

USA: Georgia: Clarke Co.: 44 individuals from 24 sites. Caught in flight trap from 9 March–21 April 2020.

##### Distribution.

Eastern United States (Muona 2000).

##### Saproxylic habits.

Larvae develop under bark (Peterson 1960) and bore through wood (McClarin 2007); develops within a variety of hardwood trees of various decomposition stages, but may prefer maple as a host and moderately decayed logs (Muona 2000; Ferro et al. 2012a).

##### Conservation.

Significantly higher abundance in secondary (= second-growth) than primary (= old-growth) forests in the southern Appalachian Mountains (Ferro et al. 2012a).

#### ﻿ Microrhagus audax

Taxon classificationAnimaliaColeopteraEucnemidae

Horn, 1886

1CC7FA83-322D-550D-B555-7F6274C10B0F

##### Collection information.

USA: Georgia: Clarke Co.: a single individual from one site. Caught in flight trap from 19 May–2 June 2020.

##### Distribution.

Eastern North America.

##### Saproxylic habits.

Larvae develop within rotten elm (Otto 2015) and adults have emerged from hardwood twigs (Ferro and Nguyen 2016).

#### ﻿ Microrhagus brunneus

Taxon classificationAnimaliaColeopteraEucnemidae

Otto, 2013

71853382-A4D2-5EF2-A919-471315372223

##### Collection information.

USA: Georgia: Clarke Co.: a single individual from one site. Caught in flight trap from 21 April–6 May 2020.

##### Distribution.

Eastern United States (Otto 2022).

##### Saproxylic habits.

Larvae develop within the wood of rotten oak logs (Otto 2015).

#### ﻿ Microrhagus carinicollis

Taxon classificationAnimaliaColeopteraEucnemidae

Otto, 2015

0D7110A1-98EB-5F56-9795-F917BAC3C2B3

##### Collection information.

USA: Georgia: Clarke Co.: 18 individuals from 14 sites. Caught in flight trap from 19 May–9 September 2020.

##### Distribution.

Eastern United States (Otto 2022).

##### Saproxylic habits.

Larvae develop within white-rotted, moist maple logs, and adults occur in oak stumps and branch debris (Otto 2015).

#### ﻿ Microrhagus triangularis

Taxon classificationAnimaliaColeopteraEucnemidae

(Say, 1823)

74BCB45E-C08A-5080-9434-55E499E81095

##### Collection information.

USA: Georgia: Clarke Co.: seven individuals from five sites. Caught in flight trap from 16 June–9 September 2020.

##### Distribution.

Eastern North America.

##### Saproxylic habits.

Larvae develop in moist, white-rotted logs, including maple and aspen (Otto 2015); adults also occur on dogwood logs (*Cornus* L. (Cornaceae)) (Muona 2000).

##### Conservation.

Rare in the Maritime Provinces of Canada, possibly due to the history of intensive forest management in the region altering forest composition and structure (Majka 2007).

#### ﻿ Nematodes atropos

Taxon classificationAnimaliaColeopteraEucnemidae

(Say, 1836)

5267DF45-F5AC-573B-A30C-5405617CB33B

##### Collection information.

USA: Georgia: Clarke Co.: 36 individuals from 19 sites. Caught in flight trap from 19 May–27 July 2020.

##### Distribution.

Eastern North America and Mexico.

##### Saproxylic habits.

larvae, pupae, and adults have been found together within the base of a dead black oak (Van Horn 1909); emerged from water oak logs (Ulyshen and Hanula 2009a), hardwood twigs (Ferro and Nguyen 2016), and American beech, elm, and maple (Dury 1904).

#### ﻿ Nematodes penetrans

Taxon classificationAnimaliaColeopteraEucnemidae

(LeConte, 1852)

2D3D0130-A5BC-5074-BD55-8CF0A429B1E9

##### Collection information.

USA: Georgia: Clarke Co.: 130 individuals from 12 sites. Caught in flight trap from 19 May–30 June 2020.

##### Distribution.

Eastern North America.

##### Saproxylic habits.

Hypermetamorphic larvae develop within firm sections of rotten branches and trunks, including sugar maple (Otto 2017); also emerged from American beech, elm, and maple (Dury 1904; Knull 1947).

##### Conservation.

Rare in the Maritime Provinces of Canada, possibly due to the history of intensive forest management in the region altering forest composition and structure (Majka 2007).

#### ﻿ Rhagomicrus bonvouloiri

Taxon classificationAnimaliaColeopteraEucnemidae

(Horn, 1886)

D922BD91-5F5E-5CFD-84A2-823289FFFD23

##### Collection information.

USA: Georgia: Clarke Co.: 12 individuals from 10 sites. Caught in flight trap from 19 May–26 August 2020.

##### Distribution.

Eastern North America (Otto 2022).

##### Saproxylic habits.

Larvae develop within white-rotted maple and wet, rotted aspen (Otto 2012b); adults also occur on pine stumps (Muona 2000) and have been found burrowing in a wet, rotting oak log (Otto 2012b).

#### ﻿ Schizophilus subrufus

Taxon classificationAnimaliaColeopteraEucnemidae

(Randall, 1838)

A181FCB5-76D4-57E4-BAF1-7DEE40D47D15

##### Collection information.

USA: **Georgia (new state record)**: Clarke Co.: two individuals from one site. Caught in flight trap from 14 July–11 August 2020.

##### Distribution.

Eastern North America.

##### Saproxylic habits.

Larvae develop within red-rotted oak logs (Otto and Young 1998).

#### ﻿ Thambus horni

Taxon classificationAnimaliaColeopteraEucnemidae

Muona, 2000

6618C113-78C8-55AE-A709-364A45FFED82

##### Collection information.

USA: **Georgia (new state record)**: Clarke Co.: three individuals from three sites. Caught in flight trap from 19 May–14 July 2020.

##### Distribution.

Eastern North America.

##### Saproxylic habits.

unknown; larvae are likely in deadwood, as typical for the family (Muona 2000).

### ﻿Histeridae

#### ﻿ Acritus exiguous

Taxon classificationAnimaliaColeopteraHisteridae

(Erichson, 1834)

230E7FB3-8B85-54F1-AF63-C880BB7012F0

##### Collection information.

USA: Georgia: Clarke Co.: 45 individuals from 25 sites. Caught in flight trap from 25 March–9 September 2020.

##### Distribution.

Eastern North America.

##### Saproxylic habits.

Feed on fungi under dead bark, including oak and elm (Savely 1939; Hoffmann 1942); emerged from oak, pine, and especially sweetgum logs and snags (Gil 2008; Ulyshen and Hanula 2009a).

#### ﻿ Aeletes floridae

Taxon classificationAnimaliaColeopteraHisteridae

(Marseul, 1862)

A9FE9DBE-9C05-5144-ACEC-B6E96B43E3A6

##### Collection information.

USA: Georgia (new state record*): Clarke Co.: a single individual from one site. Caught in flight trap from 14–27 July 2020.

##### Distribution.

Eastern North America.

##### Saproxylic habits.

Emerged from sweetgum (Ulyshen and Hanula 2009a) and loblolly pine dead for five years (Ulyshen and Hanula 2010); species of *Aeletes* Horn, 1873 are often found in association with rotting tree trunks and occasionally leaf litter (Kovarik and Caterino 2005).

#### ﻿ Bacanius punctiformis

Taxon classificationAnimaliaColeopteraHisteridae

(LeConte, 1853)

CE526A9D-0346-5A3D-B1D4-1ABC07496C6F

##### Collection information.

USA: Georgia (new state record*): Clarke Co.: nine individuals from six sites. Caught in flight trap from 19 May–26 August 2020.

##### Distribution.

Eastern North America.

##### Saproxylic habits.

Adults occur under bark and are suggested to feed on fungi (Savely 1939); emerged in great numbers from loblolly pine (throughout decomposition), sweetgum, and water oak, and was particularly associated with deadwood items near to the ground (Ulyshen and Hanula 2009a, 2010); additionally emerged from hardwood twigs (Ferro and Nguyen 2016).

##### Conservation.

Significantly associated with forests in highly forested landscapes (> 50% forest) in the Piedmont (Traylor et al. 2023a).

#### ﻿ Epierus pulicarius

Taxon classificationAnimaliaColeopteraHisteridae

Erichson, 1834

284376A6-26FF-5C87-A391-D40065E1AF81

##### Collection information.

USA: Georgia: Clarke Co.: a single individual from one site. Caught in flight trap from 19 May–2 June 2020.

##### Distribution.

Eastern North America.

##### Saproxylic habits.

Emerged from moist wood of a decayed sweetgum stump (Snow 1958), loblolly pine logs throughout decomposition (Ulyshen and Hanula 2010), hardwood twigs (Ferro and Nguyen 2016).

#### ﻿ Platysoma aequum

Taxon classificationAnimaliaColeopteraHisteridae

(LeConte, 1863)

4C006079-B46E-5294-98F1-20758911AA39

##### Collection information.

USA: Georgia: Clarke Co.: a single individual from one site. Caught in flight trap from 9–25 March 2020.

##### Distribution.

Eastern United States.

##### Saproxylic habits.

Unknown; likely prey upon dipteran larvae under bark of dead trees as do similarly shaped species of *Platysoma* Leach, 1817 (Kovarik and Caterino 2005).

#### ﻿ Platysoma aurelianum

Taxon classificationAnimaliaColeopteraHisteridae

(Horn, 1873)

C0B15FD7-3E4F-5659-9AEA-4DCC03329FAF

##### Collection information.

USA: Georgia (new state record*): Clarke Co.: nine individuals from seven sites. Caught in flight trap from 9 March–11 August 2020.

##### Distribution.

Eastern North America.

##### Saproxylic habits.

unknown; likely prey upon dipteran larvae under bark of dead trees as do similarly shaped species of *Platysoma* (Kovarik and Caterino 2005).

#### ﻿ Platysoma leconti

Taxon classificationAnimaliaColeopteraHisteridae

Marseul, 1853

C0C48BFB-1283-5E09-98E4-29C7264F130E

##### Collection information.

USA: Georgia: Clarke Co.: two individuals from two sites. Caught in flight trap from 21 April–14 July 2020.

##### Distribution.

North America.

##### Saproxylic habits.

Adults occur under bark of hardwood trees (Downie and Arnett 1996; Webster et al. 2012b); emerged from loblolly pine, water oak, and especially sweetgum (Ulyshen and Hanula 2009a), as well as hardwood twigs (Ferro and Nguyen 2016) and the non-native mimosa (*Albiziajulibrissin* (Durazz.) (Fabaceae)) (Ulyshen et al. 2018).

#### ﻿ Platysoma parallelum

Taxon classificationAnimaliaColeopteraHisteridae

(Say, 1825)

6EEBC519-AA9D-521D-8A80-E0259D35DB87

##### Collection information.

USA: Georgia: Clarke Co.: a single individual from one site. Caught in flight trap from 25 March–9 April 2020.

##### Distribution.

Eastern North America.

##### Saproxylic habits.

Predator of bark beetles in pine (Shepherd and Goyer 2005), and adults also occur under decaying elm bark (Hoffmann 1942); emerged from and associated with fresh loblolly pine logs (Ulyshen and Hanula 2010).

### ﻿Hybosoridae

#### ﻿ Ceratocanthus aeneus

Taxon classificationAnimaliaColeopteraHybosoridae

(MacLeay, 1819)

53B1B02A-1ACF-5BE4-9F71-466732206A9A

##### Collection information.

USA: Georgia: Clarke Co.: two individuals from two sites. Caught in flight trap from 27 July–26 August 2020.

##### Distribution.

Southeastern United States.

##### Saproxylic habits.

Larvae develop within the moist, rotting contents of hollow trees (Choate 1987); adults also occur under bark (Hoffman 2006).

#### ﻿ Germarostes aphodioides

Taxon classificationAnimaliaColeopteraHybosoridae

(Illiger, 1800)

B9DD03C1-07E9-50A9-A64E-0C7C9610F393

##### Collection information.

USA: Georgia: Clarke Co.: three individuals from three sites. Caught in flight trap from 21 April–14 July 2020.

##### Distribution.

Eastern United States, south to Central America.

##### Saproxylic habits.

Adults occur under bark of dead oaks (Robinson 1918), and in dying, fungus covered trees, such as tulip tree (Evans 2009); emerged from water oak and sweetgum typically at higher strata in the canopy but also associated with water oak and snags (Ulyshen and Hanula 2009a).

### ﻿Ischaliidae

#### ﻿ Ischalia costata

Taxon classificationAnimaliaColeopteraIschaliidae

(LeConte, 1861)

72EE07D4-036D-5208-8E52-CC35D1853BCE

##### Collection information.

USA: Georgia (new state record*): Clarke Co.: a single individual from one site. Sifted from leaf litter from 27–28 July 2020.

##### Distribution.

Northeastern North America, south to Georgia and Alabama.

##### Saproxylic habits.

Larvae and adults of *Ischalia* Pascoe, 1860 feed on fungal mycelia growing on rotting stumps and downed logs (Young 1985); adults are flightless (Majka 2011).

### ﻿Laemophloeidae

#### ﻿ Charaphloeus adustus

Taxon classificationAnimaliaColeopteraLaemophloeidae

(LeConte, 1854)

B7CEF629-06F1-5DA2-B94E-AFA900556C67

##### Collection information.

USA: Georgia (new state record*): Clarke Co.: 13 individuals from 10 sites. Caught in flight trap from 9 March–16 June 2020.

##### Distribution.

Eastern North America.

##### Saproxylic habits.

Emerged from ash limbs (*Fraxinus* L. (Oleaceae)) (Ulyshen et al. 2012); adults occur under bark and on dead branches (Thomas 1993; Kim 2009).

#### ﻿ Charaphloeus convexulus

Taxon classificationAnimaliaColeopteraLaemophloeidae

(LeConte, 1879)

BE48F39A-CCCD-52CD-95D7-E8FF69F9CBA2

##### Collection information.

USA: Georgia (new state record*): Clarke Co.: seven individuals from seven sites. Caught in flight trap from 9 March–9 April 2020.

##### Distribution.

Eastern North America, west to Texas and North Dakota.

##### Saproxylic habits.

Very little known; adults occur under bark (Thomas 1993).

#### ﻿ Dysmerus basalis

Taxon classificationAnimaliaColeopteraLaemophloeidae

Casey, 1884

829D5B4E-6385-5FF8-A521-497244561933

##### Collection information.

USA: **Georgia (new state record)**: Clarke Co.: 11 individuals from eight sites. Caught in flight trap from 9 March–27 July 2020.

##### Distribution.

Eastern and central United States, and Caribbean islands.

##### Saproxylic habits.

Occur under bark and in bark beetle galleries, and emerged from bark beetle infested twigs, including sweetgum, poison ivy, and poisonwood (*Metopiumtoxiferum* (L.) Krug and Urb. (Anacardiaceae)) (Thomas 2009).

#### ﻿ Laemophloeus biguttatus

Taxon classificationAnimaliaColeopteraLaemophloeidae

(Say, 1827)

AFF75D45-CFBF-52EB-BF4F-7F613BE69C16

##### Collection information.

USA: Georgia: Clarke Co.: 24 individuals from 13 sites. Caught in flight trap from 9 March–26 August 2020.

##### Distribution.

North America.

##### Saproxylic habits.

Larvae develop under the bark of standing dead oaks (e.g., *Quercushemisphaerica* W.Bartram ex Willd.) infested with the fungi *Biscogniauxiaatropunctata*, and apparently jump when disturbed (Bertone et al. 2022); adults also occur under bark (Thomas 1993).

##### Conservation.

Occurrence probability increases in old forests (predating 1938 and oak dominated) in the Piedmont (Traylor et al. 2024).

#### ﻿ Laemophloeus fasciatus

Taxon classificationAnimaliaColeopteraLaemophloeidae

Melsheimer, 1846

4358D736-11F6-5016-9779-42604845F9B0

##### Collection information.

USA: Georgia: Clarke Co.: three individuals from three sites. Caught in flight trap from 9 March–26 August 2020.

##### Distribution.

Eastern North America.

##### Saproxylic habits.

Adults occur under bark (Thomas 1993).

#### ﻿Genus Lathropus

Taxon classificationAnimaliaColeopteraLaemophloeidae

Erichson, 1846

A3E1AD3B-827B-54AF-9D25-7E7D8D046813

##### Collection information.

USA: Georgia: Clarke Co.: 18 individuals from 13 sites. Caught in flight trap from 9 March–9 September 2020.

##### Saproxylic habits.

Species of *Lathropus* occur under bark and in association with bark beetles (Thomas 1993); however, some evidence suggests the association is incidental and that *Lathropus* feed on fungi (Uliana 2003; Thomas 2010).

#### ﻿ Phloeolaemus chamaeropis

Taxon classificationAnimaliaColeopteraLaemophloeidae

(Schwarz, 1878)

CDB0A940-A55A-5873-8435-B3AAA7B83463

##### Collection information.

USA: Georgia: Clarke Co.: two individuals from two sites. Caught in flight trap from 16 June–14 July 2020.

##### Distribution.

Southeastern United States (Thomas 1993).

##### Saproxylic habits.

Adults occur under the bark of hardwood logs (sometimes freshly dead with fermenting phloem), especially oak, and apparently feed on fungi (Thomas 1993); emerged from water oak and sweetgum logs (Ulyshen and Hanula 2009a) and occur on freshly dead southern red oak (Gil 2008).

#### ﻿ Placonotus zimmermanni

Taxon classificationAnimaliaColeopteraLaemophloeidae

(LeConte, 1854)

9B615515-7698-572C-986C-49A3C59030F0

##### Collection information.

USA: Georgia: Clarke Co.: five individuals from five sites. Caught in flight trap from 9 March–14 July 2020.

##### Distribution.

Eastern North America.

##### Saproxylic habits.

Emerged from oaks, sweetgum, loblolly pine, and non-native mimosa, and especially associated with oak logs (Gil 2008; Ulyshen and Hanula 2009a; Ulyshen et al. 2018).

### ﻿Lampyridae

#### ﻿ Lucidota atra

Taxon classificationAnimaliaColeopteraLampyridae

(Olivier, 1790)

03A54DA1-1F6A-5327-83B4-15AEC32EF03B

##### Collection information.

USA: Georgia: Clarke Co.: two individuals from one site. Caught in flight trap from 19 May–30 June 2020.

##### Distribution.

Eastern North America.

##### Saproxylic habits.

Larvae inhabit damp, rotting logs, but may also wander outside of this habitat (Branham and Archangelsky 2000).

### ﻿Latridiidae

#### ﻿ Enicmus aterrimus

Taxon classificationAnimaliaColeopteraLatridiidae

Motschulsky, 1866

34210F9A-9E8B-5402-9F1C-78EA90A947C6

##### Collection information.

USA: **Georgia (new state record)**: Clarke Co.: 35 individuals from 19 sites. Caught in flight trap from 9 March–9 September 2020.

##### Distribution.

North America.

##### Saproxylic habits.

Adults and larvae of most *Enicmus* Thomson, 1859 feed on slime molds (Myxomycetes) (Andrews 2002), but it is unclear whether this is true for *E. Aterrimus* (Lawrence and Newton 1980).

#### ﻿ Enicmus maculatus

Taxon classificationAnimaliaColeopteraLatridiidae

(LeConte, 1878)

CC5835A8-7FAB-5B52-976C-2927A2503192

##### Collection information.

USA: Georgia: Clarke Co.: 289 individuals from 33 sites. Caught in flight trap from 9 March–9 September 2020.

##### Distribution.

Eastern United States.

##### Saproxylic habits.

Larvae develop in, and adults occur on *Hypoxylon* growing on oaks, including live oak (*Quercusvirginiana* Mill.) and southern red oak, apparently feeding on conidia (Lawrence 1977; Gil 2008).

##### Conservation.

Occurrence probability increases in old forests (predating 1938 and oak dominated) in the Piedmont (Traylor et al. 2024).

#### ﻿ Enicmus tenuicornis

Taxon classificationAnimaliaColeopteraLatridiidae

LeConte, 1878

83102803-1340-5435-9198-E0A7C73CB2DB

##### Collection information.

USA: **Georgia (new state record)**: Clarke Co.: six individuals from four sites. Caught in flight trap from 6 May–30 June 2020.

##### Distribution.

North America.

##### Saproxylic habits.

Feeds on slime molds in the genus *Stemonitis* Gleditsch (Stemonitaceae) (Lawrence and Newton 1980); emerged from spruce (*Picea* A.Dietr), fir (*Abies* Mill.), and pine (all in Pinaceae) in the Pacific northwest (Hatch 1961), and mainly associated with coniferous forests in Atlantic Canada (Majka et al. 2009).

### ﻿Leiodidae

#### ﻿Genus Agathidium

Taxon classificationAnimaliaColeopteraLeiodidae

Panzer, 1797

7E4B159E-DE33-5D16-B1D2-BC6E203ED4F9

##### Collection information.

USA: Georgia: Clarke Co.: 69 individuals from 21 sites, not identified to species-level. Caught in flight trap and sifted from leaf litter from 9 March–9 September 2020.

##### Saproxylic habits.

Species of *Agathidium* are specialist consumers of slime molds (Lawrence and Newton 1980) and commonly occur under bark, in wood, or on fungi (Downie and Arnett 1996).

##### Conservation.

Significantly higher abundance in primary (= old-growth) than secondary (= second-growth) forests in the southern Appalachian Mountains (Ferro et al. 2012a).

#### ﻿Genus Anisotoma

Taxon classificationAnimaliaColeopteraLeiodidae

Panzer, 1797

A5A7DBB0-2C15-53A2-AAC1-EBB708248E46

##### Collection information.

USA: Georgia: Clarke Co.: a single individual from one site, not identified to species-level. Caught in flight trap from 19 May–2 June 2020.

##### Saproxylic habits.

Species of *Anisotoma* are specialist consumers of slime molds (Lawrence and Newton 1980) and occur in decaying wood (Wheeler 1979).

### ﻿Lucanidae

#### ﻿ Platycerus quercus

Taxon classificationAnimaliaColeopteraLucanidae

(Weber, 1801)

2502A71A-DAE8-5208-800E-F472E5EAEC62

##### Collection information.

USA: Georgia: Clarke Co.: a single individual from one site. Caught in flight trap from 25 March–9 April 2020.

##### Distribution.

Eastern North America.

##### Saproxylic habits.

Larvae feed on decaying wood and develop within rotting oak and hickory logs (Hoffmann 1937; Savely 1939).

### ﻿Lycidae

#### ﻿ Eros humeralis

Taxon classificationAnimaliaColeopteraLycidae

(Fabricius, 1801)

3D7D8065-3123-572D-827B-EEBB2C48C338

##### Collection information.

USA: Georgia: Clarke Co.: a single individual from one site. Caught in flight trap from 25 March–9 April 2020.

##### Distribution.

Eastern North America.

##### Saproxylic habits.

Larvae develop within decaying wood (Bocak and Matsuda 2003; Fleming 2011) and adults sometimes aggregate under bark of stumps, such as pine (Frost 1945).

### ﻿Lymexylidae

#### ﻿ Melittomma sericeum

Taxon classificationAnimaliaColeopteraLymexylidae

(Harris, 1841)

12CF333A-3342-5F13-91BF-D3730EF51E94

##### Collection information.

USA: Georgia (new state record*): Clarke Co.: four individuals from three sites. Caught in flight trap from 16 June–27 July 2020.

##### Distribution.

Eastern North America.

##### Saproxylic habits.

Larvae bore through wounded wood, broken branches, dying trees, stumps, and freshly cut logs of oak, primarily white oak (Solomon 1995); adult females transport a symbiotic ambrosial fungus to new hosts with mycangia near their ovipositor, and this fungus grows on the walls of larval tunnels (Young 2002a); historically, *M. Sericeum* preferred American chestnut (Hopkins 1893).

### ﻿Melandryidae

#### ﻿ Anisoxya glaucula

Taxon classificationAnimaliaColeopteraMelandryidae

LeConte, 1866

EE851969-13ED-58FF-A333-C68CD6E533C6

##### Collection information.

USA: **Georgia (new state record)**: Clarke Co.: two individuals from one site. Caught in flight trap from 2–30 June 2020.

##### Distribution.

Eastern North America.

##### Saproxylic habits.

Very little known; adults occur under bark (Staines and Staines 2021); species of *Anisoxya* Mulsant, 1856 from other regions develop within dead branches and stems of hardwood trees (Nikitsky and Pollock 2010).

#### ﻿ Dircaea liturata

Taxon classificationAnimaliaColeopteraMelandryidae

LeConte, 1866

330DFE5B-84E0-5F3A-9F42-3C29847F979C

##### Collection information.

USA: Georgia: Clarke Co.: 78 individuals from 31 sites. Caught in flight trap from 21 April–9 September 2020.

##### Distribution.

Eastern North America, west to Alberta in Canada.

##### Saproxylic habits.

Emerged from moderately decayed hardwood logs (Ferro et al. 2012a) and adults occur on a variety of dead hardwood trees (Majka and Pollock 2006); larvae of *Dircaea* Fabricius, 1798 develop within soft, white-rotten wood (Nikitsky and Pollock 2010).

##### Conservation.

Significantly higher abundance in primary (= old-growth) than secondary (= second-growth) forests in the southern Appalachian Mountains (Ferro et al. 2012a).

#### ﻿ Microscapha clavicornis

Taxon classificationAnimaliaColeopteraMelandryidae

LeConte, 1866

068842F2-5A39-5272-9CAC-4B9E35311661

##### Collection information.

USA: Georgia: Clarke Co.: five individuals from four sites. Caught in flight trap from 21 April–26 August 2020.

##### Distribution.

Eastern North America.

##### Saproxylic habits.

Emerged from hardwood twigs (Ferro and Nguyen 2016); adults feed on fungi growing on rotting wood (Luk 2012).

#### ﻿ Microtonus sericans

Taxon classificationAnimaliaColeopteraMelandryidae

LeConte, 1862

36D11EE2-5915-53D1-BFBF-8A7F317DA1EE

##### Collection information.

USA: Georgia: Clarke Co.: 71 individuals from 25 sites. Caught in flight trap from 25 March–27 July 2020.

##### Distribution.

Eastern North America.

##### Saproxylic habits.

Emerged from dead hardwood pieces of a variety of sizes and decomposition stages (Ferro et al. 2012a), including southern red oak twigs (Ferro et al. 2009; Ferro and Gimmel 2014); adults also occur on dead trees and shrubs, such as hawthorn (*Crataegus* L. (Rosaceae)) (Majka and Pollock 2006).

##### Conservation.

In the Piedmont, significantly associated with forests within sparsely forested landscapes (<50% forest; Traylor et al. 2023a) and occurrence probability decreases with the amount of landscape forest cover (Traylor et al. 2024).

#### ﻿ Orchesia castanea

Taxon classificationAnimaliaColeopteraMelandryidae

Melsheimer, 1846

05BCF13D-5103-5B14-8831-521020C8B1C6

##### Collection information.

USA: Georgia (new state record*): Clarke Co.: a single individual from one site. Caught in flight trap from 19 May–2 June 2020.

##### Distribution.

Eastern North America, transcontinental in north.

##### Saproxylic habits.

Breeds in a variety of polypore fungi, including *Trametes*, *Inonotushispidus* (Bull.) P. Karst, and *Gloeophyllumsepiarium* (Wulfen) P. Karst (Gloeophyllaceae) (Weiss and West 1920); additionally emerged from rotting hickory branches dead for two to four years (Blackman and Stage 1924).

#### ﻿ Orchesia cultriformis

Taxon classificationAnimaliaColeopteraMelandryidae

Laliberté, 1967

933B89B7-2768-551D-9AD2-8071BD996029

##### Collection information.

USA: Georgia (new state record*): Clarke Co.: a single individual from one site. Caught in flight trap from 25 March–9 April 2020.

##### Distribution.

Eastern North America, transcontinental in north.

##### Saproxylic habits.

Develop within decaying polypores of parasitic fungi, including *Inocutisdryophila* (Berk.) Fiasson and Niemelä (Hymenochaetaceae) growing on oaks (Hinson 2016) and *Inonotusobliquus* (Fr.) Pilát growing on birch (Bunyard 2015).

#### ﻿Genus Phloiotrya

Taxon classificationAnimaliaColeopteraMelandryidae

Stephens, 1832

844510C5-0748-562D-A0C7-88D7CC8A4640

##### Collection information.

USA: Georgia: Clarke Co.: 22 individuals from 13 sites, unable to identify to species-level. Caught in flight trap from 21 April–30 June 2020.

##### Saproxylic habits.

Species of *Phloiotrya* develop within firm wood of deciduous trees and adults feed on fungi (Nikitsky and Pollock 2010).

#### ﻿ Spilotus quadripustulatus

Taxon classificationAnimaliaColeopteraMelandryidae

(Melsheimer, 1846)

EDEC5DD3-8AAF-51F9-A299-DF4BB43438BE

##### Collection information.

USA: Georgia (new state record*): Clarke Co.: two individuals from two sites. Caught in flight trap from 9 April–6 May 2020.

##### Distribution.

Eastern North America.

##### Saproxylic habits.

Very little known; emerged from maple wood (DiGirolomo 2015) and associated with hardwood forests in Canada (Webster et al. 2012c).

##### Conservation.

Rare in the Maritime Provinces of Canada, possibly due to the history of intensive forest management in the region altering forest composition and structure (Majka 2007).

#### ﻿ Symphora flavicollis

Taxon classificationAnimaliaColeopteraMelandryidae

(Haldeman, 1848)

C485D184-F6BC-59FE-A689-3073645E957E

##### Collection information.

USA: Georgia: Clarke Co.: six individuals from four sites. Caught in flight trap from 21 April–2 June 2020.

##### Distribution.

Eastern North America.

##### Saproxylic habits.

unknown; other species of *Symphora* LeConte, 1866 emerged from dead hardwoods (Ferro et al. 2012a).

#### ﻿ Symphora rugosa

Taxon classificationAnimaliaColeopteraMelandryidae

(Haldeman, 1848)

0916BDBB-ADB9-5E98-AA3A-48F30F269E9C

##### Collection information.

USA: Georgia: Clarke Co.: 31 individuals from 19 sites. Caught in flight trap from 6 May–11 August 2020.

##### Distribution.

Eastern North America.

##### Saproxylic habits.

Emerged from moderately decayed hardwood pieces (Ferro et al. 2012a).

### ﻿Micromalthidae

#### ﻿ Micromalthus debilis

Taxon classificationAnimaliaColeopteraMicromalthidae

LeConte, 1878

5A15C4B3-8D30-5578-A5DC-EA40CB112A49

##### Collection information.

USA: **Georgia (new state record)**: Clarke Co.: a single individual from one site. Caught in flight trap from 14–27 July 2020.

##### Distribution.

Eastern North America, but spread to other regions (Philips and Young 2000).

##### Saproxylic habits.

Develops within red-rotted hardwoods, especially oak and chestnut (Philips and Young 2000); displays a unique and complicated life cycle with separate phases of paedogenesis and parthenogenesis (see Philips and Young (2000) for overview and Barber (1913a, b) for original description).

### ﻿Monotomidae

#### ﻿Genus Bactridium

Taxon classificationAnimaliaColeopteraMonotomidae

LeConte, 1861

58BD5FA8-F65E-5279-BF85-106A961DF589

##### Collection information.

USA: Georgia: Clarke Co.: 11 individuals from nine sites, unable to identify to species-level. Caught in flight trap from 9 March–11 August 2020.

##### Saproxylic habits.

Species of *Bactridium* occur under bark of hardwoods and feed on ascomycete fungi (McElrath and McHugh 2018).

#### ﻿ Rhizophagus sayi

Taxon classificationAnimaliaColeopteraMonotomidae

Schaeffer, 1913

52DED73E-8F59-515B-B13C-3043F22B5CED

##### Collection information.

USA: Georgia: Clarke Co.: three individuals from two sites. Caught in flight trap from 9–25 March 2020.

##### Distribution.

Eastern North America.

##### Saproxylic habits.

Occur under the bark of deciduous trees where it is a predator and likely fungivore (Bousquet 1990); found in association with bark beetles (McElrath and McHugh 2018).

##### Conservation.

Significantly associated with young forests (regrown since 1938 and pine dominated) in highly forested landscapes (> 50% forest) in the Piedmont (Traylor et al. 2023a).

### ﻿Mordellidae

#### ﻿ Falsomordellistena discolor

Taxon classificationAnimaliaColeopteraMordellidae

(Melsheimer, 1846)

C3544A40-2263-52FC-BE04-B47F81D2E94A

##### Collection information.

USA: Georgia: Clarke Co.: three individuals from three sites. Caught in flight trap from 19 May–30 June 2020.

##### Distribution.

Eastern North America.

##### Saproxylic habits.

unknown; other species of *Falsomordellistena* Ermisch have been reared from dead wood (Ferro et al. 2009; Ferro et al. 2012a).

#### ﻿ Falsomordellistena hebraica

Taxon classificationAnimaliaColeopteraMordellidae

(LeConte, 1862)

2A4A64F5-6EDC-5AB7-8EB1-97D76CDEA1AA

##### Collection information.

USA: Georgia: Clarke Co.: 13,688 individuals from 40 sites. Caught in flight trap from 9 April–9 September 2020.

##### Distribution.

Eastern North America.

##### Saproxylic habits.

Emerged from southern red oak twigs (Ferro et al. 2009).

#### ﻿ Falsomordellistena pubescens

Taxon classificationAnimaliaColeopteraMordellidae

(Fabricius, 1798) (treated as a species complex)

9AEC5379-6096-5A9C-BCB2-BE7F8617B8EC

##### Collection information.

USA: Georgia: Clarke Co.: 9,313 individuals from 40 sites, including many specimens more appropriately determinable as Falsomordellistenabihamata (Melsheimer, 1846), and several types of intermediate forms of the two species. Because of the sheer number of specimens and the clear gradient between the two species observed, they were treated as a collective here. Caught in flight trap from 9 April–26 August 2020.

##### Distribution.

Eastern North America, south to Central America.

##### Saproxylic habits.

*Falsomordellistenabihamata* has emerged from small pieces of moderately decayed hardwoods (Ferro et al. 2012a).

#### ﻿ Glipodes sericans

Taxon classificationAnimaliaColeopteraMordellidae

(Melsheimer, 1846)

71EC3E70-3915-5025-BB59-38B1486E0A72

##### Collection information.

USA: Georgia: Clarke Co.: three individuals from three sites. Caught in flight trap from 30 June–9 September 2020.

##### Distribution.

Southeastern United States, south to Mexico.

##### Saproxylic habits.

Larvae develop in rotten oak logs, where they feed on the decaying wood (Savely 1939).

#### ﻿ Hoshihananomia octopunctata

Taxon classificationAnimaliaColeopteraMordellidae

(Fabricius, 1775)

D92F273D-F312-51F4-B4FE-B6ED11EEEF79

##### Collection information.

USA: Georgia: Clarke Co.: 18 individuals from 13 sites. Caught in flight trap from 19 May–11 August 2020.

##### Distribution.

Eastern North America.

##### Saproxylic habits.

Larvae develop within dead oak, hickory, and American beech (Felt 1906; Leng and Davis 1924; Ford and Jackman 1996; Jackman and Lu 2002).

#### ﻿ Mordella atrata

Taxon classificationAnimaliaColeopteraMordellidae

Melsheimer, 1846

79CF603F-5AF9-5FAC-9002-8D7A6BEEC4FA

##### Collection information.

USA: Georgia: Clarke Co.: 11 individuals from eight sites. Caught in flight trap from 19 May–14 July 2020.

##### Distribution.

North America.

##### Saproxylic habits.

Emerged from a loblolly pine log (MU pers. obs.; Traylor et al. 2023c).

#### ﻿ Mordella lunulata

Taxon classificationAnimaliaColeopteraMordellidae

Helmuth, 1865

8AD75668-9571-5088-AF63-E8F264B10431

##### Collection information.

USA: Georgia: Clarke Co.: 42 individuals from 18 sites. Caught in flight trap from 19 May–27 July 2020.

##### Distribution.

Eastern United States.

##### Saproxylic habits.

unknown, except adults beat from dead hardwood limbs (Downie and Arnett 1996); other species of *Mordella* L., 1758 develop within various types of decaying wood (reviewed in Traylor et al. 2023c).

#### ﻿ Mordella marginata

Taxon classificationAnimaliaColeopteraMordellidae

Melsheimer, 1846

823EE39E-F4E6-5C73-8003-317D588D41D2

##### Collection information.

USA: Georgia: Clarke Co.: 158 individuals from 36 sites. Caught in flight trap from 21 April–27 July 2020.

##### Distribution.

Eastern North America.

##### Saproxylic habits.

Emerged from a variety of sizes and types of dead wood from hardwood trees (Dolphin et al. 1972; Ford and Jackman 1996; Ferro et al. 2012a; Ferro and Nguyen 2016); emerged from the fungus *Gloeophyllumsepiarium* although this is an unlikely host (Weiss 1920).

#### ﻿ Mordella obliqua

Taxon classificationAnimaliaColeopteraMordellidae

LeConte, 1878

431A3647-7E70-5A4A-A04E-F826D0F7DD2F

##### Collection information.

USA: Georgia: Clarke Co.: three individuals from three sites. Caught in flight trap from 2–30 June 2020.

##### Distribution.

Eastern United States.

##### Saproxylic habits.

unknown, except adults beat from dead hardwood limbs (Downie and Arnett 1996); other species of *Mordella* develop within various types of decaying wood (reviewed in Traylor et al. 2023c).

#### ﻿ Mordellaria fascifera

Taxon classificationAnimaliaColeopteraMordellidae

(LeConte, 1878)

3C76D8C3-B4C7-506A-A448-FE6A6A4B14CC

##### Collection information.

USA: Georgia: Clarke Co.: 16 individuals from 13 sites. Caught in flight trap from 2 June–11 August 2020.

##### Distribution.

Southeastern United States (Liljeblad 1945).

##### Saproxylic habits.

unknown; other species of *Mordellaria* Ermisch, 1950 develop within various types of decaying wood (reviewed in Traylor et al. 2023c).

#### ﻿ Mordellaria serval

Taxon classificationAnimaliaColeopteraMordellidae

(Say, 1835)

CE1255DA-4CCF-5BC8-9DC0-ACBB1BF2A96C

##### Collection information.

USA: Georgia: Clarke Co.: 122 individuals from 37 sites. Caught in flight trap from 19 May–11 August 2020.

##### Distribution.

Eastern North America.

##### Saproxylic habits.

Emerged from moderately decayed hardwood logs (Ferro et al. 2012a); adults occur on various deadwood pieces, including dead pine, “ironwood” stumps, and dead American beech (Brimley 1951; Lisberg and Young 2003).

#### ﻿ Mordellaria undulata

Taxon classificationAnimaliaColeopteraMordellidae

(Melsheimer, 1846)

62DFCF3C-D389-599E-A0A5-000549FF5EA7

##### Collection information.

USA: Georgia (new state record*): Clarke Co.: 233 individuals from 36 sites. Caught in flight trap from 19 May–9 September 2020.

##### Distribution.

Eastern North America, not yet recorded from much of the southeast.

##### Saproxylic habits.

Develops within rotting elm (Hoffmann 1942); adults occur on dead hardwood branches (Downie and Arnett 1996).

#### ﻿ Mordellistena liturata

Taxon classificationAnimaliaColeopteraMordellidae

(Melsheimer, 1846)

5C520FB5-5193-52B4-8364-657CB9D19F49

##### Collection information.

USA: Georgia: Clarke Co.: 277 individuals from 37 sites. Caught in flight trap from 19 May–9 September 2020.

##### Distribution.

Eastern North America.

##### Saproxylic habits.

Emerged from fresh swamp white oak logs (*Quercusbicolor* Willd.) (Powell et al. 2016, G.S. Powell in litt.), and from moderately decayed loblolly pine logs (Ulyshen et al. 2020; Traylor et al. 2023c).

##### Conservation.

Significantly associated with forests in highly forested landscapes (> 50% forest) in the Piedmont (Traylor et al. 2023a).

#### ﻿ Mordellistena masoni

Taxon classificationAnimaliaColeopteraMordellidae

Liljeblad, 1918

902DFB08-B90A-5D9F-B4C2-BBE40D840862

##### Collection information.

USA: Georgia (new state record*): Clarke Co.: 98 individuals from 27 sites. Caught in flight trap from 2 June–11 August 2020.

##### Distribution.

Eastern North America.

##### Saproxylic habits.

Emerged from freshly cut loblolly pine logs (MU pers. obs.; Traylor et al. 2023c).

##### Conservation.

In the Piedmont, significantly associated with forests within highly forested landscapes (> 50% forest; Traylor et al. 2023a) and occurrence probability increases with the amount of landscape forest cover (Traylor et al. 2024).

#### ﻿ Mordellistena militaris

Taxon classificationAnimaliaColeopteraMordellidae

LeConte, 1862

9BA45599-DFF0-5356-A76B-ADBA96C14DC7

##### Collection information.

USA: Georgia (new state record*): Clarke Co.: 24 individuals from 12 sites. Caught in flight trap from 19 May–11 August 2020.

##### Distribution.

Eastern North America.

##### Saproxylic habits.

Emerged from decaying loblolly pine logs (Ulyshen et al. 2020; Traylor et al. 2023c).

#### ﻿ Mordellistena picipennis

Taxon classificationAnimaliaColeopteraMordellidae

Smith, 1882

DE5A0D14-4712-5ABC-B0AC-97456E3A9D48

##### Collection information.

USA: Georgia: Clarke Co.: six individuals from two sites. Caught in flight trap from 2–16 June 2020.

##### Distribution.

Eastern United States (Liljeblad 1945).

##### Saproxylic habits.

Breeds in chestnut (Liljeblad 1945).

#### ﻿ Mordellistena tosta

Taxon classificationAnimaliaColeopteraMordellidae

LeConte, 1862

88DD3531-8AFB-5192-807B-0C9EFE33B29B

##### Collection information.

USA: Georgia: Clarke Co.: 16 individuals from nine sites. Caught in flight trap from 19 May–30 June 2020.

##### Distribution.

Eastern North America.

##### Saproxylic habits.

Emerged from decayed loblolly pine logs (Ulyshen et al. 2020; Traylor et al. 2023c), and from alder (*Alnus* Mill. (Betulaceae)) (Liljeblad 1945).

##### Conservation.

Significantly associated with, and occurrence probability increases in, young forests (regrown since 1938 and pine dominated) in the Piedmont (Traylor et al. 2023a, 2024).

#### ﻿ Mordellistena trifasciata

Taxon classificationAnimaliaColeopteraMordellidae

(Say, 1826)

E3A29FA5-09AD-5D09-AB43-E0A2058DE05C

##### Collection information.

USA: Georgia: Clarke Co.: 305 individuals from 39 sites. Caught in flight trap from 21 April–9 September 2020.

##### Distribution.

Eastern North America, south to Central America.

##### Saproxylic habits.

Emerged from a sweetgum log (Ulyshen and Hanula 2009a; Traylor et al. 2023c).

#### ﻿ Mordellochroa scapularis

Taxon classificationAnimaliaColeopteraMordellidae

(Say, 1824)

517D0E26-19AC-53C7-A85E-41AF151C7BAA

##### Collection information.

USA: **Georgia (new state record)**: Clarke Co.: 352 individuals from 38 sites. Caught in flight trap from 9 March–16 June 2020.

##### Distribution.

Eastern North America, transcontinental in the north.

##### Saproxylic habits.

Emerged from fresh hardwood logs (Ferro et al. 2012a).

#### ﻿ Paramordellaria triloba

Taxon classificationAnimaliaColeopteraMordellidae

(Say, 1824)

E69683E8-41E6-52AF-98B4-3B941EA2052F

##### Collection information.

USA: Georgia (new state record*): Clarke Co.: six individuals from four sites. Caught in flight trap from 2 June–27 July 2020.

##### Distribution.

Eastern North America.

##### Saproxylic habits.

Emerged from a small diameter, decaying hardwood (Ferro et al. 2012a); adults occur on dead hardwoods, including “ironwood” and black cherry (Brimley 1951).

#### ﻿ Yakuhananomia bidentata

Taxon classificationAnimaliaColeopteraMordellidae

(Say, 1824)

3DA7E23F-5691-581A-96D1-CB05AC7E8336

##### Collection information.

USA: Georgia: Clarke Co.: two individuals from two sites. Caught in flight trap from 2 June–27 July 2020.

##### Distribution.

Eastern North America.

##### Saproxylic habits.

Emerged from fungus-infested wood (Hoffmann 1942); adults occur on dead and dying hardwood trees including oak, hickory, and American beech (Leng and Davis 1924; Brimley 1951; Downie and Arnett 1996).

### ﻿Murmidiidae

#### ﻿ Mychocerinus depressus

Taxon classificationAnimaliaColeopteraMurmidiidae

(LeConte, 1866)

92768B2B-8D4F-5D96-9D53-3DE8E69CB8DB

##### Collection information.

USA: Georgia: Clarke Co.: a single individual from one site. Caught in flight trap from 21 April–6 May 2020.

##### Distribution.

Eastern North America.

##### Saproxylic habits.

Adults occur under fungus-covered bark of oak, beech, and hickory (Lawrence and Stephan 1975); emerged from water oak, loblolly pine, and sweetgum, and associated with standing dead trees (Ulyshen and Hanula 2009a).

### ﻿Mycetophagidae

#### ﻿ Litargus sexpunctatus

Taxon classificationAnimaliaColeopteraMycetophagidae

(Say, 1826)

83AF4A16-6DE0-5827-BF5F-25930FB26215

##### Collection information.

USA: Georgia: Clarke Co.: 12 individuals from nine sites. Caught in flight trap from 9 March–11 August 2020.

##### Distribution.

Eastern North America.

##### Saproxylic habits.

Larvae develop, and adults occur on *Hypoxylon* growing on oak, where they feed on conidia (Lawrence 1977); adults also occur on other fungi, such as *Pleurotusostreatus* (Jacq.) P. Kumm. (Cline and Leschen 2005); emerged from and associated with dead oaks in upland forests (Ulyshen and Hanula 2009a).

##### Conservation.

In the Piedmont, significantly associated with forests within highly forested landscapes (> 50% forest; Traylor et al. 2023a) and occurrence probability increases with the amount of landscape forest cover (Traylor et al. 2024).

#### ﻿ Mycetophagus pluripunctatus

Taxon classificationAnimaliaColeopteraMycetophagidae

LeConte, 1856

458D3003-9718-5543-8BCB-B40574808E15

##### Collection information.

USA: Georgia (new state record*): Clarke Co.: two individuals from two sites. Caught in flight trap from 9 March–9 April 2020.

##### Distribution.

Eastern North America.

##### Saproxylic habits.

Adults occur in or on fleshy and gilled polypore fungi on standing dead trees and logs (Ciegler 2014), including *Climacodonseptentrionalis* (Fr.) P. Karst (Meruliaceae) (Webster et al. 2012c).

### ﻿Nitidulidae

#### ﻿ Amphicrossus ciliatus

Taxon classificationAnimaliaColeopteraNitidulidae

(Olivier, 1811)

13CAFD1D-5DA3-5118-9DE2-9BEFC4C3E478

##### Collection information.

USA: Georgia: Clarke Co.: 420 individuals from 39 sites. Caught in flight trap from 9 March–9 September 2020.

##### Distribution.

Eastern North America.

##### Saproxylic habits.

Larvae develop within slime fluxes (fermenting sap flows) on tree injuries, and adults occur in this habitat too (Cole and Streams 1970); adults occur at sap flows on oaks and maples, and on fleshy fungus (Price and Young 2006).

##### Conservation.

Occurrence probability increases in old forests (predating 1938 and oak dominated) in the Piedmont (Traylor et al. 2024).

### ﻿Nosodendridae

#### ﻿ Nosodendron unicolor

Taxon classificationAnimaliaColeopteraNosodendridae

Say, 1824

2D14525B-2616-5AF4-8B48-1A923EA6D234

##### Collection information.

USA: Georgia: Clarke Co.: 15 individuals from 12 sites. Caught in flight trap from 9 March–26 August 2020.

##### Distribution.

Eastern United States.

##### Saproxylic habits.

Larvae develop within slime fluxes on hardwood trees, and adults occur in this habitat as well (Hayes and Chu 1946); the beetles feed on fermenting sap enriched with microorganisms and any predation is likely facultative (Ivie 2002a).

##### Conservation.

Occurrence probability increases in old forests (predating 1938 and oak dominated) in the Piedmont (Traylor et al. 2024).

### ﻿Passandridae

#### ﻿ Catogenus rufus

Taxon classificationAnimaliaColeopteraPassandridae

(Fabricius, 1798)

F38FAAF4-0DE5-5ED5-AD09-701B46126DF8

##### Collection information.

USA: Georgia: Clarke Co.: seven individuals from six sites. Caught in flight trap from 25 March–26 August 2020.

##### Distribution.

Eastern North America, south to Central America.

##### Saproxylic habits.

Larvae parasitize the pupae of woodboring beetles, and adults can occur under loose bark of dead and dying hardwood and conifer trees (Dimmock 1882; Fiske 1905).

### ﻿Ptinidae

#### ﻿ Byrrhodes fallax

Taxon classificationAnimaliaColeopteraPtinidae

(Fall, 1905)

E0C6913C-F77F-5798-9F8A-4E028AE20E7A

##### Collection information.

USA: Georgia (new state record*): Clarke Co.: 10 individuals from nine sites. Caught in flight trap from 19 May–26 August 2020.

##### Distribution.

Southeastern United States (White 1982).

##### Saproxylic habits.

Unknown; species of *Byrrhodes* Leconte, 1878 occur in hard (persistent) tree fungi (Lawrence and de Viedma 1991; Philips 2002).

#### ﻿ Byrrhodes incomptus

Taxon classificationAnimaliaColeopteraPtinidae

(LeConte, 1865)

0B0CDC84-3DC0-54D5-808B-3F62778EFEBD

##### Collection information.

USA: Georgia (new state record*): Clarke Co.: 59 individuals from 20 sites. Caught in flight trap from 21 April–27 July 2020.

##### Distribution.

Eastern North America (White 1982).

##### Saproxylic habits.

Very little known; emerged from fungus (Arango and Young 2012).

#### ﻿ Byrrhodes intermedius

Taxon classificationAnimaliaColeopteraPtinidae

(LeConte, 1878)

652459F5-2A70-5397-8175-6DB856D44D1D

##### Collection information.

USA: Georgia (new state record*): Clarke Co.: 37 individuals from 17 sites. Caught in flight trap from 9 April–26 August 2020.

##### Distribution.

Eastern North America (White 1982).

##### Saproxylic habits.

Emerged from *Ganodermaapplanatum* (Pers.) Pat. Growing on a dead elm (Arango and Young 2012), and adults also occur on other shelf fungi, including *Fomesfomentarius* (Böving 1954).

##### Conservation.

Rare in the Maritime Provinces of Canada, possibly due to the history of intensive forest management in the region altering forest composition and structure (Majka 2007).

#### ﻿ Byrrhodes tristriatus

Taxon classificationAnimaliaColeopteraPtinidae

(LeConte, 1878)

39B7AC35-DC5F-5AD0-B91E-CFD7F5755232

##### Collection information.

USA: Georgia: Clarke Co.: 15 individuals from 11 sites. Caught in flight trap from 21 April–9 September 2020.

##### Distribution.

Eastern North America.

##### Saproxylic habits.

Reproduce in old brackets of *Inonotuscuticularis* (Bull.) P.Karst. (Weiss and West 1922).

#### ﻿ Calymmaderus nitidus

Taxon classificationAnimaliaColeopteraPtinidae

(LeConte, 1865)

0E44BB92-2B03-5BF8-B0C1-82E200A058DE

##### Collection information.

USA: Georgia: Clarke Co.: three individuals from three sites. Caught in flight trap from 2 June–14 July 2020.

##### Distribution.

Eastern North America.

##### Saproxylic habits.

Emerged from southern red oak and other hardwood twigs (Ferro et al. 2009; Ferro and Nguyen 2016).

#### ﻿ Dorcatoma falli

Taxon classificationAnimaliaColeopteraPtinidae

White, 1965

69BFEB31-A2C4-5D03-B08A-F17C1C2299FD

##### Collection information.

USA: **Georgia (new state record)**: Clarke Co.: 35 individuals from 13 sites. Caught in flight trap from 9 April–2 June 2020.

##### Distribution.

Eastern North America.

##### Saproxylic habits.

unknown; species of *Dorcatoma* Hebrst, 1972 occur in large, persistent, and often woody polypore fungi (Lawrence and de Viedma 1991; Philips 2002).

#### ﻿ Eucrada humeralis

Taxon classificationAnimaliaColeopteraPtinidae

(Melsheimer, 1846)

6FB1B781-80C2-5786-ADE8-107DDCF5A74D

##### Collection information.

USA: **Georgia (new state record)**: Clarke Co.: a single individual from one site. Caught in flight trap from 9–21 April 2020.

##### Distribution.

Eastern North America.

##### Saproxylic habits.

Mine under bark of dead oak trees (Rozen 1958).

#### ﻿ Euvrilletta mucorea

Taxon classificationAnimaliaColeopteraPtinidae

(LeConte, 1865)

6D6BEF32-3D91-5F11-82F2-5AD3E5DCBBCD

##### Collection information.

USA: **Georgia (new state record)**: Clarke Co.: a single individual from one site. Caught in flight trap from 2–16 June 2020.

##### Distribution.

Southeastern United States.

##### Saproxylic habits.

unknown; species of *Euvrilleta* Van Dyke, 1946 develop within decayed wood of both hardwood and conifer trees (Baker 1972; Arango and Young 2012).

#### ﻿ Euvrilletta peltata

Taxon classificationAnimaliaColeopteraPtinidae

(Harris, 1836)

6A21D817-0CF8-59E4-9D1E-FCD08842D4D3

##### Collection information.

USA: Georgia: Clarke Co.: five individuals from five sites. Caught in flight trap from 2 June–30 June 2020.

##### Distribution.

Eastern North America.

##### Saproxylic habits.

Bores through the rotten sapwood and heartwood of hardwoods and conifers (Baker 1972).

##### Conservation.

Rare in the Maritime Provinces of Canada, possibly due to the history of intensive forest management in the region altering forest composition and structure (Majka 2007).

#### ﻿ Hadrobregmus notatus

Taxon classificationAnimaliaColeopteraPtinidae

(Say, 1825)

8F63540C-F56E-5DDE-8C42-DE5B1D5123AF

##### Collection information.

USA: Georgia: Clarke Co.: a single individual from one site. Caught in flight trap from 25 March–9 April 2020.

##### Distribution.

Eastern North America.

##### Saproxylic habits.

Larvae develop within dead and rotten oak, ash, chestnut, and pine (Böving 1954; White 1982).

#### ﻿ Hemicoelus carinatus

Taxon classificationAnimaliaColeopteraPtinidae

(Say, 1823)

A3EF9C3F-F048-52CA-84A3-FD3EDD2A5AC7

##### Collection information.

USA: Georgia (new state record*): Clarke Co.: a single individual from one site. Caught in flight trap from 6–19 May 2020.

##### Distribution.

Eastern North America, transcontinental in the north.

##### Saproxylic habits.

Develops within a variety of dead hardwood and conifer trees (Simeone 1960), including freshly-dead and decayed wood (Arango and Young 2012).

#### ﻿ Oligomerus alternans

Taxon classificationAnimaliaColeopteraPtinidae

LeConte, 1865

8638E99E-0C53-5858-B4DA-F2502C44AD78

##### Collection information.

USA: Georgia (new state record*): Clarke Co.: a single individual from one site. Caught in flight trap from 19 May–2 June 2020.

##### Distribution.

Eastern North America (Arango and Young 2012).

##### Saproxylic habits.

Unknown; species of *Oligomerus* Redtenbacher, 1849 have been reared from various hardwoods (Philips 2002; Arango and Young 2012).

#### ﻿ Oligomerus sericans

Taxon classificationAnimaliaColeopteraPtinidae

(Melsheimer, 1846)

03414621-9988-570B-988B-B171FB338E7C

##### Collection information.

USA: Georgia: Clarke Co.: a single individual from one site. Caught in flight trap from 2–16 June 2020.

##### Distribution.

Eastern North America.

##### Saproxylic habits.

Not well known, hosts are thought to be walnut (*Juglans* L.), chestnut, and white oak (Fall 1905; Böving 1954; White 1982).

#### ﻿ Petalium bistriatum

Taxon classificationAnimaliaColeopteraPtinidae

(Say, 1825)

0A2919D6-845C-504A-A3EC-9F85921D778E

##### Collection information.

USA: Georgia: Clarke Co.: 488 individuals from 35 sites. Caught in flight trap from 21 April–9 September 2020.

##### Distribution.

Eastern North America.

##### Saproxylic habits.

Emerged from dry sections of black oak and bear oak (*Quercusilicifolia* Wangenh.), with the bear oak record apparently coming from outer bark of a living tree (Ford 1973).

##### Conservation.

Significantly associated with old forests (predating 1938 and oak dominated) in the Piedmont (Traylor et al. 2023a).

#### ﻿ Petalium debile

Taxon classificationAnimaliaColeopteraPtinidae

Fall, 1905

C154719B-254A-5E91-8B60-31903A139452

##### Collection information.

USA: Georgia: Clarke Co.: a single individual from one site. Caught in flight trap from 2–16 June 2020.

##### Distribution.

Eastern United States.

##### Saproxylic habits.

Emerged from southern red oak twigs (Ferro et al. 2009; Ferro and Gimmel 2014).

#### ﻿ Petalium incisum

Taxon classificationAnimaliaColeopteraPtinidae

Ford, 1973

879532E7-9AC1-510C-AE59-72B61818A07A

##### Collection information.

USA: Georgia: Clarke Co.: 176 individuals from 28 sites. Caught in flight trap and sifted from leaf litter from 19 May–9 September 2020.

##### Distribution.

Eastern North America.

##### Saproxylic habits.

Emerged from dead, dry sections of poison ivy, black locust (*Robiniapseudoacacia* L.), and staff vine (*Celastrus* L. (Celastraceae)) (Ford 1973).

#### ﻿ Petalium whitei

Taxon classificationAnimaliaColeopteraPtinidae

Ford, 1973

E9B2E100-AD73-599A-A6F1-721F976830FB

##### Collection information.

USA: Georgia: Clarke Co.: 13 individuals from 11 sites. Caught in flight trap from 9 March–30 June 2020.

##### Distribution.

Eastern North America.

##### Saproxylic habits.

unknown; other species of *Petalium* LeConte reproduce in dry deadwood (Ford 1973).

#### ﻿ Priobium sericeum

Taxon classificationAnimaliaColeopteraPtinidae

(Say, 1825)

F4E42B9B-EC83-5C9A-99C6-043BE3D44E88

##### Collection information.

USA: Georgia: Clarke Co.: two individuals from two sites. Caught in flight trap from 16–30 June 2020.

##### Distribution.

Eastern North America.

##### Saproxylic habits.

Larvae develop within dead branches of oak, cherry, hickory, holly (*Ilex* L. (Aquifoliaceae)), and mountain laurel (*Kalmialatifolia* L. (Ericaceae)) (White 1982), and apparently use very dry wood (McClarin 2005); emerged from large and small hardwood pieces and associated with fresh wood (Ferro et al. 2012a; Ferro and Nguyen 2016).

##### Conservation.

Significantly higher abundance in primary (= old-growth) than secondary (= second-growth) forests in the southern Appalachian Mountains (Ferro et al. 2012a).

#### ﻿Genus Ptilinus

Taxon classificationAnimaliaColeopteraPtinidae

Mueller, 1764

0E59D7D4-FEF0-5731-ADD0-AE1FBBAC6005

##### Collection information.

USA: Georgia: Clarke Co.: five individuals from four sites, not identified to species-level (females). Caught in flight trap from 21 April–19 May 2020.

##### Saproxylic habits.

Larvae of *Ptilinus* feed in dead hardwood trees and shrubs (White 1982).

#### ﻿ Trichodesma gibbosa

Taxon classificationAnimaliaColeopteraPtinidae

(Say, 1825)

46F9E286-2017-597F-92EC-08C3A4F66BAA

##### Collection information.

USA: Georgia: Clarke Co.: four individuals from four sites. Caught in flight intercept trap from 21 April–2 June 2020.

##### Distribution.

Eastern North America.

##### Saproxylic habits.

Larvae develop within a variety of dead hardwood trees (White 1982); emerged from both fresh and decayed hickory, including the decaying walls of a tree hollow (Blackman and Stage 1924; Holland 2009).

#### ﻿ Trichodesma klagesi

Taxon classificationAnimaliaColeopteraPtinidae

Fall, 1905

E711D6B1-C7C2-5832-A80E-BE50BE96A692

##### Collection information.

USA: Georgia (new state record*): Clarke Co.: a single individual from one site. Caught in flight trap from 2–16 June 2020.

##### Distribution.

Eastern North America (Hinson and Blinn 2018).

##### Saproxylic habits.

Emerged from hickory and dead stems of spice bush (*Linderabenzoin* (L.) Blume (Lauraceae)) (White 1982), as well as dead hardwood pieces of various diameter and decomposition stages (Ferro et al. 2012a).

#### ﻿ Tricorynus gracilis

Taxon classificationAnimaliaColeopteraPtinidae

(Fall, 1905)

65EF232E-CB60-5D69-AB39-99BD00195528

##### Collection information.

USA: **Georgia (new state record)**: Clarke Co.: a single individual from one site. Caught in flight trap from 25 March–9 April 2020.

##### Distribution.

Eastern North America (White 1982).

##### Saproxylic habits.

Larvae and adults collected from the fungus *Camilleatinctor* (Berk.) Læssøe, J.D. Rogers and Whalley (Xylariaceae) growing on the bark of a dead maple (Böving 1954; White 1963).

#### ﻿ Tricorynus nigritulus

Taxon classificationAnimaliaColeopteraPtinidae

(LeConte, 1865)

D71A3FB5-D9F7-5C58-B21B-D8E97B895C44

##### Collection information.

USA: Georgia (new state record*): Clarke Co.: 18 individuals from 10 sites. Caught in flight trap from 19 May–26 August 2020.

##### Distribution.

Eastern North America (White 1982).

##### Saproxylic habits.

Emerged from dead wood of elm and dead vines of *Wisteria* Nutt. (Fabaceae) (Böving 1954; White 1963).

#### ﻿ Tricorynus punctatus

Taxon classificationAnimaliaColeopteraPtinidae

(LeConte, 1865)

FA8CD473-EC2C-54F9-BC4E-14B1BB94803D

##### Collection information.

USA: Georgia: Clarke Co.: 262 individuals from 40 sites. Caught in flight trap from 21 April–11 August 2020.

##### Distribution.

Eastern North America (White 1982).

##### Saproxylic habits.

Emerged from old sycamore logs and grape vines (*Vitis* L.) (White 1963).

### ﻿Pyrochroidae

#### ﻿ Dendroides canadensis

Taxon classificationAnimaliaColeopteraPyrochroidae

Latreille, 1810

40DFB86B-4EB6-559B-95E9-AA3F36C2B05A

##### Collection information.

USA: Georgia: Clarke Co.: a single individual from one site. Caught in flight trap from 19 May–2 June 2020.

##### Distribution.

Eastern North America.

##### Saproxylic habits.

Larvae develop under loose bark on the upper side of decaying logs (Young 2002b).

##### Conservation.

Significantly higher abundance in primary (= old-growth) than secondary (= second-growth) forests in the southern Appalachian Mountains (Ferro et al. 2012a).

#### ﻿ Neopyrochroa femoralis

Taxon classificationAnimaliaColeopteraPyrochroidae

(LeConte, 1855)

D54BF04A-789C-53C9-813E-0B9DCADFFCD5

##### Collection information.

USA: Georgia: Clarke Co.: two individuals from two sites. Caught in flight trap from 25 March–9 April 2020.

##### Distribution.

Eastern North America.

##### Saproxylic habits.

Larvae develop under bark and in decayed wood of standing dead trees (Young 2002b).

##### Conservation.

Rare in the Maritime Provinces of Canada, possibly due to the history of intensive forest management in the region altering forest composition and structure (Majka 2007).

#### ﻿ Neopyrochroa flabellata

Taxon classificationAnimaliaColeopteraPyrochroidae

(Fabricius, 1787)

D6AF15C5-5136-5D6A-9BA1-F2E677AB4A3D

##### Collection information.

USA: Georgia: Clarke Co.: a single individual from one site. Caught in flight trap from 2–16 June 2020.

##### Distribution.

Eastern North America.

##### Saproxylic habits.

Larvae develop under bark and in decaying wood of logs, usually on the underside that is resting on the soil (Young 2002b).

### ﻿Salpingidae

#### ﻿ Inopeplus reclusa

Taxon classificationAnimaliaColeopteraSalpingidae

(LeConte, 1880)

107DFFD5-0C19-527D-A829-3998766415EB

##### Collection information.

USA: Georgia (new state record*): Clarke Co.: two individuals from two sites. Caught in flight trap from 19 May–2 June 2020.

##### Distribution.

Southeastern United States.

##### Saproxylic habits.

Emerged from loblolly pine logs (Ulyshen 2014); adults beaten from dead branches of a button bush (probably *Cephalanthus* L. (Rubiaceae)) (Blatchley 1918).

### ﻿Scarabaeidae

#### ﻿ Gnorimella maculosa

Taxon classificationAnimaliaColeopteraScarabaeidae

(Knoch, 1801)

6DFF8A28-966F-5CB5-B3B4-9B42A9DF50DA

##### Collection information.

USA: Georgia: Clarke Co.: eight individuals from eight sites. Caught in flight trap from 25 March–9 April 2020.

##### Distribution.

Eastern North America.

##### Saproxylic habits.

Emerged from moderately to well-decayed hardwood substrates, such as the hollowed interior of a redbud (*Cercis* L. (Fabaceae)) trunk or rotten logs (Ritcher 1966; Ferro et al. 2012a); adults occur on dead trees, such as red maple (*Acerrubrum* L.) (Staines 1984).

##### Conservation.

Occurrence probability increases in old forests (predating 1938 and oak dominated) in the Piedmont (Traylor et al. 2024).

#### ﻿ Valgus canaliculatus

Taxon classificationAnimaliaColeopteraScarabaeidae

(Olivier, 1789)

68B3B83A-ED6B-5311-A0D8-CB93BD64784A

##### Collection information.

USA: Georgia: Clarke Co.: 480 individuals from 37 sites. Caught in flight trap from 25 March–27 July 2020.

##### Distribution.

Eastern United States.

##### Saproxylic habits.

Associated with subterranean termites (*Reticulitermesflavipes* (Kollar, 1837) (Blattodea: Rhinotermitidae)), and larvae develop by feeding on the walls of the termite galleries (Ritcher 1958); adults occur under bark of standing dead trees, such as mockernut hickory (*Caryatomentosa* Nutt.) and pine (Howden and Vogt 1951; Steury and Paulsen 2022).

#### ﻿ Valgus seticollis

Taxon classificationAnimaliaColeopteraScarabaeidae

(Palisot de Beauvois, 1805)

5DABCFEB-75B6-5AEC-992F-CC72C19EFB2D

##### Collection information.

USA: Georgia: Clarke Co.: two individuals from two sites. Caught in flight trap from 9 March–21 April 2020.

##### Distribution.

Eastern United States.

##### Saproxylic habits.

Associated with subterranean termites (*R.flavipes*), and larvae develop by feeding on the walls of termite galleries (Ritcher 1958).

### ﻿Scraptiidae

#### ﻿ Canifa pallipes

Taxon classificationAnimaliaColeopteraScraptiidae

(Melsheimer, 1846)

A4B60365-617A-5C37-BD1C-9B21D852C012

##### Collection information.

USA: Georgia (new state record*): Clarke Co.: 63 individuals from 28 sites. Caught in flight trap from 25 March–14 July 2020.

##### Distribution.

Eastern North America, transcontinental in the north.

##### Saproxylic habits.

Emerged from dead hardwood trees, such as elm (Hoffmann 1942) and oak dead for 2–3 years (McClarin 2008a); also emerged in numbers from black knot fungus (*Apiosporinamorbosa* (Schwein.) Arx (Venturiaceae)) growing on cherry trees (Melvin et al. 1967).

##### Conservation.

Occurrence probability increases in old forests (predating 1938 and oak dominated) in the Piedmont (Traylor et al. 2024).

#### ﻿ Canifa plagiata

Taxon classificationAnimaliaColeopteraScraptiidae

(Melsheimer, 1846)

41E2AEE3-A78F-5446-A4B0-1D29D1DABFE3

##### Collection information.

USA: Georgia: Clarke Co.: 10 individuals from five sites. Caught in flight trap from 9 April–2 June 2020.

##### Distribution.

Eastern North America.

##### Saproxylic habits.

unknown; larvae of *Canifa* LeConte, 1866 develop under the bark of dead logs (Pollock 2002).

##### Conservation.

Occurrence probability increases with the amount of landscape forest cover in the Piedmont (Traylor et al. 2024).

#### ﻿ Canifa pusilla

Taxon classificationAnimaliaColeopteraScraptiidae

(Haldeman, 1848)

222B5493-073F-5D84-8ABF-1FDCB718EBEE

##### Collection information.

USA: Georgia (new state record*): Clarke Co.: four individuals from three sites. Caught in flight trap from 16 June–14 July 2020.

##### Distribution.

Eastern North America.

##### Saproxylic habits.

unknown; larvae of *Canifa* develop under the bark of dead logs (Pollock 2002).

#### ﻿ Pentaria trifasciata

Taxon classificationAnimaliaColeopteraScraptiidae

(Melsheimer, 1846)

E4CC6A1C-6863-52BE-BEFC-5962DB6F2271

##### Collection information.

USA: Georgia (new state record*): Clarke Co.: a single individual from one site. Caught in flight trap from 19 May–2 June 2020.

##### Distribution.

North America.

##### Saproxylic habits.

Adults occur under rotting logs (Schneider 2017) and among other habitats (e.g., flowers, Downie and Arnett 1996).

### ﻿Silvanidae

#### ﻿ Cathartosilvanus imbellis

Taxon classificationAnimaliaColeopteraSilvanidae

(LeConte, 1854)

2FF06C7C-DCB8-51A2-9E9B-545A0CB4DA06

##### Collection information.

USA: Georgia: Clarke Co.: five individuals from three sites. Caught in flight trap from 19 May–11 August 2020.

##### Distribution.

Eastern North America.

##### Saproxylic habits.

Occur almost exclusively under bark, especially of oaks (Thomas 1993); emerged from loblolly pine, water oak, southern red oak, ash, and sweetgum (Gil 2008; Ulyshen and Hanula 2009a, 2010; Ulyshen et al. 2012); may rapidly colonize freshly burned logs (Ulyshen et al. 2010).

#### ﻿ Nausibius major

Taxon classificationAnimaliaColeopteraSilvanidae

Zimmermann, 1869

5E2F5299-270C-5302-A6C5-92B6911ED74A

##### Collection information.

USA: Georgia: Clarke Co.: 34 individuals from eight sites. Caught in flight trap from 25 March–26 August 2020.

##### Distribution.

Eastern United States, west to Arizona, and south to Mexico.

##### Saproxylic habits.

Adults occur under the bark of old oaks and rotten trees, and at slime fluxes on oaks (Thomas 1993; Valentine 2007).

##### Conservation.

In the Piedmont, significantly associated with old forests (predating 1938 and oak dominated) in highly forested landscapes (> 50% forest; Traylor et al. 2023a) and occurrence probability increases in old forests (predating 1938 and oak dominated) and with landscape forest cover (Traylor et al. 2024).

#### ﻿ Nausibius repandus

Taxon classificationAnimaliaColeopteraSilvanidae

LeConte, 1866

56E0DEAD-5C3A-5E31-8B14-9A608EA79C58

##### Collection information.

USA: **Georgia (new state record)**: Clarke Co.: a single individual from one site. Caught in flight trap from 6–19 May 2020.

##### Distribution.

Southeastern United States.

##### Saproxylic habits.

Emerged from and occur in pines infested with bark beetles (Moser et al. 1971; Thomas 1993), including a recently dead longleaf pine (*Pinuspalustris* Mill.) (Davis and Leng 1912).

#### ﻿ Silvanus muticus

Taxon classificationAnimaliaColeopteraSilvanidae

Sharp, 1899

9C651747-EFD7-5E92-9435-99846077713B

##### Collection information.

USA: Georgia: Clarke Co.: six individuals from six sites. Caught in flight trap from 25 March–26 August 2020.

##### Distribution.

Eastern North America.

##### Saproxylic habits.

Adults occur under bark of various trees (pine, maple, oak, chestnut, and juniper (*Juniperus*)), likely feeding on fungal spores (Halstead 1973); emerged and collected from loblolly pine and southern red oak logs throughout decomposition (Gil 2008; Ulyshen and Hanula 2010), as well as hardwood twigs (Ferro and Nguyen 2016); associated with deadwood in upland forests (Ulyshen and Hanula 2009a).

### ﻿Sphindidae

#### ﻿ Sphindus americanus

Taxon classificationAnimaliaColeopteraSphindidae

LeConte, 1866

321DAB70-41BC-51F0-9F9E-35B4E098E757

##### Collection information.

USA: **Georgia (new state record)**: Clarke Co.: 15 individuals from 11 sites. Caught in flight trap and sifted from leaf litter from 25 March–9 September 2020.

##### Distribution.

North America.

##### Saproxylic habits.

All life stages are dependent on slime molds growing on dead wood (Lawrence and Newton 1980; Stephenson et al. 1994; Majka 2010).

### ﻿Synchroidae

#### ﻿ Synchroa punctata

Taxon classificationAnimaliaColeopteraSynchroidae

Newman, 1838

0F125F65-7466-5B8F-98FF-884A95FC7427

##### Collection information.

USA: **Georgia (new state record)**: Clarke Co.: eight individuals from seven sites. Caught in flight trap from 2 June–9 September 2020.

##### Distribution.

Eastern North America.

##### Saproxylic habits.

Larvae consume fungal material and rotting wood (Payne 1931; Savely 1939); adults occur in deadwood of numerous hardwoods and pine (Blackman and Stage 1924).

### ﻿Tenebrionidae

#### ﻿Genus Adelina

Taxon classificationAnimaliaColeopteraTenebrionidae

Dejean, 1835

5275FBBE-915A-5BB1-8AB3-CE8D9B8A486D

##### Collection information.

USA: Georgia: Clarke Co.: two individuals from two sites, not identified to species (females). Caught in flight trap from 9 March–9 April 2020.

##### Saproxylic habits.

Larvae and adults of *Adelina* occur under bark (Doyen 1988; Ulyshen and Hanula 2009a; Ciegler 2014).

#### ﻿ Androchirus femoralis

Taxon classificationAnimaliaColeopteraTenebrionidae

(Olivier, 1795)

B62B0588-EF4F-5C1C-9993-8D30129E869B

##### Collection information.

USA: Georgia: Clarke Co.: 11 individuals from eight sites. Caught in flight trap from 19 May–14 July 2020.

##### Distribution.

Eastern United States.

##### Saproxylic habits.

Little known, adults have been found on stumps (Blatchley 1910); larvae of *Androchirus* LeConte, 1862 develop within dead bracket fungi (Luk 2007).

#### ﻿ Diaperis maculata

Taxon classificationAnimaliaColeopteraTenebrionidae

Olivier, 1791

CC8DF063-AB1D-5F53-A364-557EDAA2DE62

##### Collection information.

USA: Georgia: Clarke Co.: a single individual from one site. Caught in flight trap from 11–26 August 2020.

##### Distribution.

Eastern North America, south to Central America, and Caribbean islands.

##### Saproxylic habits.

Larvae develop within polypore fungi and adults occur in fungi or under bark (Park 1931; Wolcott and Montgomery 1933; Daggy 1946; Graves 1960).

##### Conservation.

Occurrence apparently stable from 1900–present on Plummers Island, Maryland, despite losses of other species possibly due to changing forest conditions (Steiner 2008).

#### ﻿Genus Hymenorus

Taxon classificationAnimaliaColeopteraTenebrionidae

Mulsant, 1852

FA6E35EB-8F88-524B-A446-4E6D6008C482

##### Collection information.

See individual species below.

##### Saproxylic habits.

Little is known about the specific habits of each species, however, larvae of *Hymenorus* develop in decaying wood of both hardwoods and conifers, including tree knots and dead portions of living trees (Wolcott and Montgomery 1933; White 1983; Majka et al. 2008; Ferro et al. 2012a), and adults often occur under bark (Fall 1931; Dunford and Young 2004; Ciegler 2014).

#### ﻿ Hymenorus discretus

Taxon classificationAnimaliaColeopteraTenebrionidae

Casey, 1891

3032097C-CCEB-5176-906B-D2EC127A845B

##### Collection information.

USA: Georgia: Clarke Co.: two individuals from two sites. Caught in flight trap from 16 June–14 July 2020.

##### Distribution.

Eastern United States.

##### Saproxylic habits.

unknown; an association with leaf-cutting ants (*Attatexana* (Buckley, 1860) (Hymenoptera: Formicidae)) (Walter et al. 1938) is in error (Waller and Moser 1990).

#### ﻿ Hymenorus humeralis

Taxon classificationAnimaliaColeopteraTenebrionidae

LeConte, 1866

FF422067-90E5-5051-B43E-E78C12E2A9C0

##### Collection information.

USA: **Georgia (new state record)**: Clarke Co.: two individuals from two sites. Caught in flight trap from 16 June–14 July 2020.

##### Distribution.

Eastern United States.

##### Saproxylic habits.

Unknown.

##### Conservation.

Apparently recently colonized Plummers Island, Maryland, since the early 1900s, despite the losses of other species possibly due to changing forest conditions (Steiner 2008).

#### ﻿ Hymenorus illusus

Taxon classificationAnimaliaColeopteraTenebrionidae

Fall, 1931

ECEF4BCD-B722-5C5B-B3BA-F78093A6B42F

##### Collection information.

USA: Georgia: Clarke Co.: two individuals from two sites. Caught in flight trap from 16 June–14 July 2020.

##### Distribution.

Eastern United States.

##### Saproxylic habits.

Adult occur in fungi (Fall 1931).

##### Conservation.

Apparently lost after 1925 from the fauna of Plummers Island, Maryland, possibly due to changing forest conditions (Steiner 2008).

#### ﻿ Hymenorus niger

Taxon classificationAnimaliaColeopteraTenebrionidae

(Melsheimer, 1846)

19EA34AD-56DB-5F84-9182-7E0CE28708B2

##### Collection information.

USA: Georgia: Clarke Co.: 40 individuals from 19 sites. Caught in flight trap from 6 May–26 August 2020.

##### Distribution.

Eastern North America.

##### Saproxylic habits.

unknown.

##### Conservation.

Apparently recently colonized Plummers Island, Maryland, since the early 1900s, despite the losses of other species possibly due to changing forest conditions (Steiner 2008).

#### ﻿ Hymenorus obesus

Taxon classificationAnimaliaColeopteraTenebrionidae

Casey, 1891

7DEAED61-BEB1-5EF2-8901-1ADD418B7926

##### Collection information.

USA: Georgia: Clarke Co.: three individuals from two sites. Caught in flight trap from 30 June–14 July 2020.

##### Distribution.

Eastern North America.

##### Saproxylic habits.

Adults occur under loose bark (Fall 1931).

##### Conservation.

Apparently recently colonized Plummers Island, Maryland, since the early 1900s, despite the losses of other species possibly due to changing forest conditions (Steiner 2008).

#### ﻿ Hymenorus perforatus

Taxon classificationAnimaliaColeopteraTenebrionidae

Casey, 1891

53563EFF-CDA4-5E74-9FA7-7625EA2225A5

##### Collection information.

USA: Georgia: Clarke Co.: 41 individuals from 17 sites. Caught in flight trap from 19 May–26 August 2020.

##### Distribution.

Eastern United States.

##### Saproxylic habits.

unknown.

##### Conservation.

Apparently recently colonized Plummers Island, Maryland, since the early 1900s, despite the losses of other species possibly due to changing forest conditions (Steiner 2008); occurrence probability increases in old forests (predating 1938 and oak dominated) in the Piedmont (Traylor et al. 2024).

#### ﻿ Hymenorus picipennis

Taxon classificationAnimaliaColeopteraTenebrionidae

Casey, 1891

B09C4B6A-732C-5A15-A01D-0810F79D2A86

##### Collection information.

USA: **Georgia (new state record)**: Clarke Co.: three individuals from two sites. Caught in flight trap from 2 June–14 July 2020.

##### Distribution.

Eastern North America.

##### Saproxylic habits.

Emerged from red oak (*Quercusrubra* L.) (Majka et al. 2008) and eastern white pine (Fall 1931); adults occur in rotting hardwood logs (Dunford and Young 2004; Garrick et al. 2019).

#### ﻿ Hymenorus pilosus

Taxon classificationAnimaliaColeopteraTenebrionidae

(Melsheimer, 1846)

835FBB67-E105-5EB7-BEE1-1856972D7882

##### Collection information.

USA: Georgia: Clarke Co.: 16 individuals from 10 sites. Caught in flight trap from 2 June–11 August 2020.

##### Distribution.

Eastern North America.

##### Saproxylic habits.

unknown.

##### Conservation.

Apparently recently colonized Plummers Island, Maryland, since the early 1900s, despite the losses of other species possibly due to changing forest conditions (Steiner 2008).

#### ﻿ Lobopoda erythrocnemis

Taxon classificationAnimaliaColeopteraTenebrionidae

(Germar, 1824)

7EB90E9A-4AC5-54F2-83B0-22BBDEAA54F2

##### Collection information.

USA: Georgia: Clarke Co.: 72 individuals from 22 sites. Caught in flight trap from 9 March–9 September 2020.

##### Distribution.

Southeastern United States.

##### Saproxylic habits.

Emerged from loblolly pine logs 1–5 years after tree death (Ulyshen and Hanula 2009a, 2010); adults occur under bark and on dead standing pines (Campbell 1966).

#### ﻿ Lobopoda punctulata

Taxon classificationAnimaliaColeopteraTenebrionidae

(Melsheimer, 1846)

437203B8-27BA-56A8-9222-5AA9E6A2EC92

##### Collection information.

USA: Georgia: Clarke Co.: 22 individuals from eight sites. Caught in flight trap from 19 May–11 August 2020.

##### Distribution.

Eastern United States.

##### Saproxylic habits.

Larvae develop within the rotting interior of living trees (Craighead 1950); adults feed on fungal spores and occur on dead oak limbs and under bark (Campbell 1966).

##### Conservation.

Apparently lost after 1925 from the fauna of Plummers Island, Maryland, possibly due to changing forest conditions (Steiner 2008).

#### ﻿ Mycetochara fraterna

Taxon classificationAnimaliaColeopteraTenebrionidae

(Say, 1824)

0C11BF53-7E4F-5CE3-BF2B-0210D4C6CA09

##### Collection information.

USA: Georgia (new state record*): Clarke Co.: four individuals from four sites. Caught in flight trap from 21 April–2 June 2020.

##### Distribution.

Eastern United States, transcontinental in Canada.

##### Saproxylic habits.

Adults occur under bark of aspen (Campbell 1978); species of *Mycetochara* Berthold, 1827 have been reared from the decaying interior of a living maple tree (McClarin 2008b).

##### Conservation.

Apparently lost after 1925 from the fauna of Plummers Island, Maryland, possibly due to changing forest conditions (Steiner 2008).

#### ﻿Genus Neomida

Taxon classificationAnimaliaColeopteraTenebrionidae

Latreille, 1829

1D002D4A-EE7E-57B1-9476-D61502FE21D9

##### Collection information.

USA: Georgia: Clarke Co.: a single individual from one site, unable to be identified to species-level due to damage. Caught in flight trap from 9–25 March 2020.

##### Saproxylic habits.

All life stages occur in various fungi where the larvae develop, and adults also occur under bark of pine and oak (Ciegler 2014).

#### ﻿ Platydema ellipticum

Taxon classificationAnimaliaColeopteraTenebrionidae

(Fabricius, 1798)

0F6AB93E-3FDB-5C4E-A5E1-A9B0EA1433A3

##### Collection information.

USA: Georgia: Clarke Co.: a single individual from one site. Caught in flight trap from 25 March–9 April 2020.

##### Distribution.

Eastern United States.

##### Saproxylic habits.

Larvae develop within the shelf fungus *F. Gilva* (Weiss 1919).

##### Conservation.

Occurrence apparently stable from 1900–present on Plummers Island, Maryland, despite losses of other species possibly due to changing forest conditions (Steiner 2008).

#### ﻿ Platydema micans

Taxon classificationAnimaliaColeopteraTenebrionidae

Zimmermann, 1870

4B13C1A6-938B-5A18-B591-1582349FDFF4

##### Collection information.

USA: Georgia: Clarke Co.: two individuals from two sites. Sifted from leaf litter from 29 June–31August 2020.

##### Distribution.

Eastern United States, Central America, and Caribbean Islands.

##### Saproxylic habits.

Adults occur in decaying fleshy fungi (Blatchley 1910) and under rotten wood (Triplehorn 1965).

#### ﻿ Platydema picilabrum

Taxon classificationAnimaliaColeopteraTenebrionidae

Melsheimer, 1846

33133018-1A4E-5513-9A49-CA78B6FBA227

##### Collection information.

USA: Georgia: Clarke Co.: two individuals from two sites. Caught in flight trap from 9 March–11 August 2020.

##### Distribution.

Eastern United States.

##### Saproxylic habits.

Emerged from a standing dead water oak (Ulyshen and Hanula 2009a); adults also occur within dead tree trunks (Ciegler 2014).

##### Conservation.

Apparently lost after 1925 from the fauna of Plummers Island, Maryland, possibly due to changing forest conditions (Steiner 2008).

#### ﻿ Platydema subcostatum

Taxon classificationAnimaliaColeopteraTenebrionidae

Laporte and Brullé, 1831

271B57D4-7ECD-5AC2-BE07-641A8660FFC8

##### Collection information.

USA: Georgia: Clarke Co.: a single individual from one site. Caught in flight trap from 25 March–9 April 2020.

##### Distribution.

Eastern North America.

##### Saproxylic habits.

Emerged from water oak and sweetgum (Ulyshen and Hanula 2009a); adults aggregate under bark, feeding on fungi (Savely 1939), as well as in dead logs and tree trunks (Ciegler 2014).

##### Conservation.

Apparently lost after 1925 from the fauna of Plummers Island, Maryland, possibly due to changing forest conditions (Steiner 2008).

#### ﻿ Platydema teleops

Taxon classificationAnimaliaColeopteraTenebrionidae

Triplehorn, 1965

62E09D55-5D72-5B9A-BB21-8B4EEB379CF2

##### Collection information.

USA: Georgia (new state record*): Clarke Co.: 16 individuals from nine sites. Caught in flight trap from 9 March–30 June 2020.

##### Distribution.

Eastern North America.

##### Saproxylic habits.

Adults occur under bark, on deadwood debris, and on fungi (Dunford and Young 2004; Webster et al. 2012d; Ciegler 2014).

##### Conservation.

Apparently lost after 1925 from the fauna of Plummers Island, Maryland, possibly due to changing forest conditions (Steiner 2008).

#### ﻿ Polypleurus perforatus

Taxon classificationAnimaliaColeopteraTenebrionidae

(Germar, 1824)

5F060D51-4230-5B35-A67C-ED98796D78AE

##### Collection information.

USA: Georgia: Clarke Co.: a single individual from one site. Sifted from leaf litter from 27–28 July 2020.

##### Distribution.

Southeastern United States.

##### Saproxylic habits.

Larvae develop within smaller, dry branches, and adults are flightless (Steiner 1999); adults occur under dead bark and logs, including oak and pine (Ciegler 2014).

##### Conservation.

Apparently lost after 1925 from the fauna of Plummers Island, Maryland, possibly due to changing forest conditions (Steiner 2008); considered an indicator of undisturbed, open forest conditions (Steiner 1999).

#### ﻿ Rhipidandrus paradoxus

Taxon classificationAnimaliaColeopteraTenebrionidae

(Beauvois, 1820)

79CD0A5B-AEC9-521C-92AE-12DC18F3820D

##### Collection information.

USA: Georgia: Clarke Co.: a single individual from one site. Caught in flight trap from 25 March–9 April 2020.

##### Distribution.

Eastern North America.

##### Saproxylic habits.

Adults occur on oyster mushrooms (Cline and Leschen 2005) and toothed fungi (*Hericium* Pers. (Hericiaceae) and *Hydnellum* P.Karst (Thelephoraceae)) (Ciegler 2014).

##### Conservation.

Apparently lost after 1925 from the fauna of Plummers Island, Maryland, possibly due to changing forest conditions (Steiner 2008).

#### ﻿ Strongylium tenuicolle

Taxon classificationAnimaliaColeopteraTenebrionidae

(Say, 1827)

065D115E-8903-50F3-9475-1EE57408FBE0

##### Collection information.

USA: Georgia: Clarke Co.: two individuals from two sites. Caught in flight trap from 19 May–30 June 2020.

##### Distribution.

Eastern North America, west to Arizona (Johnston and Cortés Hernández 2021).

##### Saproxylic habits.

Larvae develop within decaying wood of various hardwood trees, including rotting portions of living trees (Triplehorn and Spilman 1973).

##### Conservation.

Occurrence apparently stable from 1900–present on Plummers Island, Maryland, despite the loss of other species possibly due to changing forest conditions (Steiner 2008).

#### ﻿ Uloma mentalis

Taxon classificationAnimaliaColeopteraTenebrionidae

Horn, 1870

B327C946-E43B-5865-B088-83E6CBF01993

##### Collection information.

USA: Georgia: Clarke Co.: a single individual from one site. Caught in flight trap from 2–16 June 2020.

##### Distribution.

Eastern North America.

##### Saproxylic habits.

Emerged from hardwood twigs (Ferro and Nguyen 2016); adults occur under bark (Ciegler 2014).

##### Conservation.

Apparently lost after 1975 from the fauna of Plummers Island, Maryland, possibly due to changing forest conditions (Steiner 2008).

### ﻿Tetratomidae

#### ﻿ Eustrophopsis bicolor

Taxon classificationAnimaliaColeopteraTetratomidae

(Fabricius, 1798)

9378D666-8AA1-57AD-A4EB-0DCF2B1D71A3

##### Collection information.

USA: Georgia: Clarke Co.: a single individual from one site. Caught in flight trap from 30 June–14 July 2020.

##### Distribution.

North America.

##### Saproxylic habits.

Adults occur under bark, in decaying wood or in hollows of a variety of trees, as well as in or on polypore fungi (Pollock 2012; Ciegler 2014).

#### ﻿ Eustrophus tomentosus

Taxon classificationAnimaliaColeopteraTetratomidae

Say, 1827

32A4BE97-E17C-5776-9595-A077E22C81D3

##### Collection information.

USA: Georgia: Clarke Co.: 105 individuals from 23 sites. Caught in flight trap from 25 March–9 September 2020.

##### Distribution.

North America.

##### Saproxylic habits.

Adults occur under bark, in decaying wood or in hollows of a variety of trees, in addition to in or on polypore fungi (Pollock 2012; Ciegler 2014).

##### Conservation.

Rare in the Maritime Provinces of Canada, possibly due to the history of intensive forest management in the region altering forest composition and structure (Majka 2007).

#### ﻿ Tetratoma tessellata

Taxon classificationAnimaliaColeopteraTetratomidae

Melsheimer, 1844

0AAC0301-E328-52F6-9407-FCA590387BB3

##### Collection information.

USA: Georgia (new state record*): Clarke Co.: three individuals from two sites. Caught in flight trap from 9 April–6 May 2020.

##### Distribution.

Eastern North America.

##### Saproxylic habits.

Occur on fungi growing on dead trees (birch, maple, oak) (Chantal 1985; Majka and Pollock 2006), including a dried oyster mushroom growing on a sugar maple (Webster et al. 2012c).

### ﻿Throscidae

#### ﻿ Aulonothroscus convergens

Taxon classificationAnimaliaColeopteraThroscidae

(Horn, 1885)

64C14C3F-1EFA-5AA9-8F3A-CDC3BB9AA4CB

##### Collection information.

USA: Georgia: Clarke Co.: 45 individuals from 19 sites. Caught in flight trap from 9 March–9 September 2020.

##### Distribution.

Eastern United States (Blanchard 1917).

##### Saproxylic habits.

Emerged from loblolly pine throughout decomposition (Ulyshen and Hanula 2010) and hardwood twigs (Ferro and Nguyen 2016).

#### ﻿ Aulonothroscus distans

Taxon classificationAnimaliaColeopteraThroscidae

Blanchard, 1917

801C96A5-790E-5B65-9886-A011CBEA6BC9

##### Collection information.

USA: **Georgia (new state record)**: Clarke Co.: two individuals from two sites. Caught in flight trap from 21 April–6 May 2020.

##### Distribution.

Eastern North America.

##### Saproxylic habits.

Emerged in number from dead hardwoods and associated with freshly dead wood (Ferro et al. 2012a).

##### Conservation.

Significantly higher abundance in secondary (= second-growth) than primary (= old-growth) forests in the southern Appalachian Mountains (Ferro et al. 2012a).

#### ﻿ Aulonothroscus parallelus

Taxon classificationAnimaliaColeopteraThroscidae

Blanchard, 1917

59B46461-A770-5A4B-B63E-798C4F4F4559

##### Collection information.

USA: Georgia (new state record*): Clarke Co.: 25 individuals from 12 sites. Caught in flight trap from 25 March–27 July 2020.

##### Distribution.

Southeastern United States (previously known from Virginia and unspecified “southern” localities (Blanchard 1917)).

##### Saproxylic habits.

Unknown; species of *Aulonothroscus* Horn, 1890 emerged from deadwood and adults occur under bark of dead hardwoods and conifers, including standing dead trees (Howden and Vogt 1951; Ulyshen and Hanula 2010; Ferro et al. 2012a).

### ﻿Trogossitidae

#### ﻿ Airora cylindrica

Taxon classificationAnimaliaColeopteraTrogossitidae

(Audinet-Serville, 1828)

702A603A-3842-503F-93BE-E2BC762F9DB6

##### Collection information.

USA: Georgia: Clarke Co.: a single individual from one site. Caught in flight trap from 16–30 June 2020.

##### Distribution.

Eastern North America.

##### Saproxylic habits.

Adults occur under bark of pine, oak, and hickory (Barron 1971); emerged from loblolly pine, water oak, and sweetgum, and associated with pine and snags (Ulyshen and Hanula 2009a).

#### ﻿ Corticotomus cylindricus

Taxon classificationAnimaliaColeopteraTrogossitidae

(LeConte, 1863)

48CE5682-7D16-572B-A42D-B98E55A945E7

##### Collection information.

USA: Georgia: Clarke Co.: three individuals from two sites. Caught in flight trap from 25 March–19 May 2020.

##### Distribution.

Eastern United States.

##### Saproxylic habits.

Predator and associate of bark and ambrosia beetles (Blackman 1922; Thatcher 1960; Barron 1971); reared from, and occur on a variety of dead hardwoods and pine (Barron 1971).

#### ﻿ Corticotomus parallelus

Taxon classificationAnimaliaColeopteraTrogossitidae

(Melsheimer, 1844)

5B585516-B23B-58C6-B1C3-DFCD62A8CE71

##### Collection information.

USA: Georgia: Clarke Co.: two individuals from two sites. Caught in flight trap from 25 March–19 May 2020.

##### Distribution.

Eastern United States.

##### Saproxylic habits.

Mostly unknown, occur under bark of pine and emerged from sumac (*Rhus* L. (Anacardiaceae)) (Barron 1971).

#### ﻿ Temnoscheila acuta

Taxon classificationAnimaliaColeopteraTrogossitidae

(LeConte, 1858)

1DBDE3E7-B717-544E-AE08-2DAFAB34C277

##### Collection information.

USA: **Georgia (new state record)**: Clarke Co.: two individuals from two sites. Caught in flight trap from 16 June–14 July 2020.

##### Distribution.

Eastern United States and Mexico (Barron 1971).

##### Saproxylic habits.

Adults occur under dead bark and on logs, including pine (Barron 1971; Dajoz 1989); adults and larvae of *Temnoscheila* Westwood, 1830 are predatory on woodboring beetle larvae under the bark of dead trees and shrubs (Kolibáč 2013).

#### ﻿ Tenebroides bimaculatus

Taxon classificationAnimaliaColeopteraTrogossitidae

(Melsheimer, 1844)

0550B412-5400-5C8E-A383-A4B6647222BC

##### Collection information.

USA: Georgia: Clarke Co.: six individuals from four sites. Caught in flight trap from 21 April–14 July 2020.

##### Distribution.

Eastern United States.

##### Saproxylic habits.

Larvae are predators of woodboring larvae (Champlain and Knull 1923); emerged from water oak (Ulyshen and Hanula 2009a); adults and larvae also occur under bark, including oak and elm (Hoffmann 1942; Barron 1971).

#### ﻿ Tenebroides corticalis

Taxon classificationAnimaliaColeopteraTrogossitidae

(Melsheimer, 1844)

935A4D53-7073-5467-9A5D-F590763F81D4

##### Collection information.

USA: Georgia: Clarke Co.: nine individuals from eight sites. Caught in flight trap from 9 March–9 September 2020.

##### Distribution.

North and Central America.

##### Saproxylic habits.

Adults and larvae occur within a wide variety of tree species, decomposition stages, and sizes of deadwood, where they are predators of woodboring insects and bark beetles (Blackman and Stage 1924; Hoffmann 1942; Barron 1971); associated with logs and lower boles of dead trees (Ulyshen and Hanula 2009a).

#### ﻿ Tenebroides laticollis

Taxon classificationAnimaliaColeopteraTrogossitidae

(Horn, 1862)

A6CDF491-95FC-5AC1-A5F7-7ADFE181FA0B

##### Collection information.

USA: Georgia: Clarke Co.: two individuals from two sites. Caught in flight trap from 14 July–26 August 2020.

##### Distribution.

Eastern North America.

##### Saproxylic habits.

Emerged from and associated with water oak (Ulyshen and Hanula 2009a); adults occur under bark of dead oak and apple (*Malus* Mill. (Rosaceae)), and on the fungus *Laetiporussulphureus* (Bull.) Murrill (Laetiporaceae) (Barron 1971).

#### ﻿ Tenebroides rugosipennis

Taxon classificationAnimaliaColeopteraTrogossitidae

(Horn, 1862)

40F115CE-D129-53E8-831F-2AAB519291F1

##### Collection information.

USA: Georgia: Clarke Co.: 50 individuals from 19 sites. Caught in flight trap from 9 March–19 May 2020.

##### Distribution.

Eastern United States, west to Arizona (Barron 1971).

##### Saproxylic habits.

Occur under bark of dead oaks (Barron 1971).

##### Conservation.

Occurrence probability increases in old forests (predating 1938 and oak dominated) in the Piedmont (Traylor et al. 2024).

#### ﻿ Tenebroides semicylindricus

Taxon classificationAnimaliaColeopteraTrogossitidae

(Horn, 1862)

197BA241-21E3-587A-B648-CD7DF0E79313

##### Collection information.

USA: Georgia: Clarke Co.: a single individual from one site. Caught in flight trap from 14–27 July 2020.

##### Distribution.

Eastern United States.

##### Saproxylic habits.

Emerged from water oak, loblolly pine, and sweetgum, and was associated with the crowns of standing dead trees (Ulyshen and Hanula 2009a).

#### ﻿ Thymalus marginicollis

Taxon classificationAnimaliaColeopteraTrogossitidae

Chevrolat, 1842

E8A0A1D1-05E5-5885-9231-537742282EAE

##### Collection information.

USA: Georgia: Clarke Co.: three individuals from three sites. Caught in flight trap from 21 April–19 May 2020.

##### Distribution.

Eastern North America, transcontinental in Canada.

##### Saproxylic habits.

Larvae develop within polypore fungi (e.g., the birch polypore (*Fomitopsisbetulina* (Bull.) B.K. Cui, M.L. Han and Y.C. Dai (Fomitopsidaceae)), and adults occur in association with it as well (Brues 1927; Minch 1952); adults also occur on other fungi, including *Daedaleaconfragosa* Berk. (Fomitopsidaceae), *Cerrenaunicolor* (Bull.) Murrill (Cerrenaceae), and *Trametesversicolor* (L.) Lloyd (Barron 1971).

### ﻿Zopheridae

#### ﻿ Aulonium parallelopipedum

Taxon classificationAnimaliaColeopteraZopheridae

(Say, 1826)

D9427AA6-314D-5713-941F-CA7AA16EBFF5

##### Collection information.

USA: Georgia: Clarke Co.: two individuals from two sites. Caught in flight trap from 19 May–16 June 2020.

##### Distribution.

Eastern United States.

##### Saproxylic habits.

Emerged from sweetgum and water oak snags (Ulyshen and Hanula 2009a); adults occur on dead logs or under bark, including oak, beech, and elm (Hopkins 1893; Robinson 1918; Hoffmann 1942; Gil 2008); species of *Aulonium* Erichson, 1845 are likely fungivores that facultatively prey upon bark beetles (Ivie 2002b).

#### ﻿ Bitoma quadricollis

Taxon classificationAnimaliaColeopteraZopheridae

(Horn, 1885)

B24959DA-BE06-5A50-BFA3-1915354CF09E

##### Collection information.

USA: Georgia: Clarke Co.: 25 individuals from 15 sites. Caught in flight trap from 9 March–11 August 2020.

##### Distribution.

Eastern North America.

##### Saproxylic habits.

Larvae develop in *Hypoxylon* fungus growing on oaks, where they were consuming fungal tissue (Lawrence 1977); emerged from pine and especially oaks (Ulyshen and Hanula 2009a); adults most commonly reside under bark of freshly killed oaks, but also maple and beech (Stephan 1989; Gil 2008).

##### Conservation.

Significantly associated with, and occurrence probability increases in, old forests (predating 1938 and oak dominated) in the Piedmont (Traylor et al. 2023a, 2024).

#### ﻿ Bitoma quadriguttata

Taxon classificationAnimaliaColeopteraZopheridae

(Say, 1826)

04CBECBA-1C35-56A1-A5E0-C5C3858F8D8B

##### Collection information.

USA: Georgia: Clarke Co.: 24 individuals from 15 sites. Caught in flight trap from 9 March–11 August 2020.

##### Distribution.

Eastern North America.

##### Saproxylic habits.

Emerged from sweetgum, loblolly pine, and especially oak in upland forests (Ulyshen and Hanula 2009a, 2010); adults occur under bark of various hardwoods and occasionally pines (Stephan 1989).

#### ﻿ Colydium lineola

Taxon classificationAnimaliaColeopteraZopheridae

Say, 1826

9026916D-DB99-5369-8847-3614DFB41F8C

##### Collection information.

USA: Georgia: Clarke Co.: 11 individuals from nine sites. Caught in flight trap from 25 March–11 August 2020.

##### Distribution.

Eastern and coastal western North America.

##### Saproxylic habits.

Adults and larvae inhabit bark beetle tunnels (Hopkins 1893; Hoffmann 1942), although reported predation may be circumstantial or facultative (Ivie 2002b); prefers deadwood at the base of trees (Stephan 1989; Ulyshen and Hanula 2009a); emerged from oaks, loblolly pine, hickory, and various other hardwoods (Blackman and Stage 1924; Stephan 1989; Gil 2008; Ulyshen and Hanula 2009a).

#### ﻿ Endeitoma dentata

Taxon classificationAnimaliaColeopteraZopheridae

(Horn, 1885)

B74F3975-A879-5E8C-99BB-F610EEF68A54

##### Collection information.

USA: Georgia: Clarke Co.: a single individual from one site. Sifted from leaf litter from 29–30 June 2020.

##### Distribution.

Southeastern United States.

##### Saproxylic habits.

Adults occur in rotting pine logs and under bark of oaks (Stephan 1989); rapidly colonizes burned loblolly pine logs (Ulyshen et al. 2010).

#### ﻿ Endeitoma granulata

Taxon classificationAnimaliaColeopteraZopheridae

(Say, 1826)

23B504D7-A993-54E1-A52C-EBAF59FDCF32

##### Collection information.

USA: Georgia: Clarke Co.: 10 individuals from eight sites. Caught in flight trap from 25 March–2 June 2020.

##### Distribution.

Eastern North America.

##### Saproxylic habits.

Emerged from loblolly pine logs (Ulyshen and Hanula 2009a) and hardwood twigs (Ferro and Nguyen 2016); adults occur where mold is growing under loose bark of hardwoods (especially oaks) and pines (Stephan 1989; Gil 2008).

#### ﻿ Namunaria guttulata

Taxon classificationAnimaliaColeopteraZopheridae

(LeConte, 1863)

F4742AFD-92E9-5124-AFF9-175678D2E771

##### Collection information.

USA: Georgia: Clarke Co.: seven individuals from seven sites. Caught in flight trap from 27 July–9 September 2020.

##### Distribution.

Eastern North America.

##### Saproxylic habits.

Occur on fungal growth under the bark of dead hardwoods (e.g., beech) and pines, including snags (Hopkins 1893; Howden and Vogt 1951; Stephan 1989); emerged from loblolly pine, sweetgum, and water oak logs and snags (Ulyshen and Hanula 2009a), and from ash limbs in the canopy (Ulyshen et al. 2012).

##### Conservation.

Significantly associated with old forests (predating 1938 and oak dominated) in highly forested landscapes (> 50% forest) in the Piedmont (Traylor et al. 2023a).

#### ﻿ Paha laticollis

Taxon classificationAnimaliaColeopteraZopheridae

(LeConte, 1863)

8189F980-B7BC-5E1B-9C1D-D636C82B514B

##### Collection information.

USA: Georgia: Clarke Co.: six individuals from six sites. Caught in flight trap and sifted from leaf litter from 9 March–30 June 2020.

##### Distribution.

Eastern United States.

##### Saproxylic habits.

Emerged from smaller hardwood branches and twigs (Ferro et al. 2012a; Ferro and Nguyen 2016) and collected from loblolly pine logs (Gil 2008); adults occur in white-rotten logs, in tree holes, and under bark, especially at the base of oaks (Stephan 1989).

#### ﻿ Pycnomerus haematodes

Taxon classificationAnimaliaColeopteraZopheridae

(Fabricius, 1801)

6C4102F7-6617-52D8-AFE8-A9E55E5EB6BB

##### Collection information.

USA: Georgia: Clarke Co.: four individuals from four sites. Caught in flight trap from 21 April–30 June 2020.

##### Distribution.

Eastern United States.

##### Saproxylic habits.

Adults and larvae occur under bark and in the wood of moist, rotting pines, and occasionally in bark beetle galleries (Savely 1939; Howden and Vogt 1951; Stephan 1989); emerged and collected from pine, oak, and sweetgum, but associated with pines and occurred throughout decomposition (Gil 2008; Ulyshen and Hanula 2009a, 2010); emerged from burned and unburned loblolly pine logs (Ulyshen et al. 2010).

#### ﻿ Pycnomerus reflexus

Taxon classificationAnimaliaColeopteraZopheridae

(Say, 1826)

768355B8-2355-5318-9420-57C5556FB6AF

##### Collection information.

USA: Georgia: Clarke Co.: nine individuals from eight sites. Caught in flight trap from 25 March–9 September 2020.

##### Distribution.

Eastern North America.

##### Saproxylic habits.

Adults and larvae occur under bark of rotting oaks and pine (Savely 1939; Stephan 1989; Gil 2008); emerged from loblolly pine, sweetgum, and especially oaks, and associated with logs at base of dead trees (Ulyshen and Hanula 2009a).

#### ﻿ Pycnomerus sulcicollis

Taxon classificationAnimaliaColeopteraZopheridae

LeConte, 1863

6FA89C78-1745-5C4F-AFB3-55EB966CA6F1

##### Collection information.

USA: Georgia: Clarke Co.: three individuals from three sites. Caught in flight trap from 2 June–26 August 2020.

##### Distribution.

Eastern United States.

##### Saproxylic habits.

Under bark and in moist, rotting wood of hardwoods (especially oaks and hickory), and occasionally pines (Stephan 1989; Gil 2008); emerged from oaks, loblolly pine, sweetgum logs and hardwood twigs (Ulyshen and Hanula 2009a; Ferro and Nguyen 2016); emerged throughout loblolly pine decomposition and associated with the base and fallen logs of dead pine trees (Ulyshen and Hanula 2009a, 2010).

##### Conservation.

Significantly associated with young forests (regrown since 1938 and pine dominated) in highly forested landscapes (> 50% forest) in the Piedmont (Traylor et al. 2023a).

#### ﻿ Synchita fuliginosa

Taxon classificationAnimaliaColeopteraZopheridae

Melsheimer, 1846

751AA3CA-60FF-52DC-ABAE-16DA2E9479BE

##### Collection information.

USA: Georgia: Clarke Co.: 47 individuals from 26 sites. Caught in flight trap from 21 April–9 September 2020.

##### Distribution.

Eastern North America.

##### Saproxylic habits.

Larvae and adults occur under bark and feed on fungal growth, including chestnut blight (*Cryphonectriaparasitica* (Murrill.) Barr (Cryphonectriaceae) (Craighead 1920); emerged from, and occurs under bark of dead elm, maple, oak, pine, hickory, and birch (Savely 1939; Hoffmann 1942; Stephan 1989; Gil 2008), and associated with the wood at the base of dead sweetgums (Ulyshen and Hanula 2009a).

#### ﻿ Synchita parvula

Taxon classificationAnimaliaColeopteraZopheridae

Guérin-Méneville, 1844

E9D6C242-C868-5868-9266-29D6FDE780CA

##### Collection information.

USA: Georgia: Clarke Co.: 567 individuals from 40 sites. Caught in flight trap from 9 March–9 September 2020.

##### Distribution.

Eastern United States.

##### Saproxylic habits.

Emerged and collected from oaks (southern red oak and water oak) (Gil 2008; Ulyshen and Hanula 2009a) and associated with dead oaks (Stephan 1989).

##### Conservation.

significantly associated with old forests (predating 1938 and oak dominated) in the Piedmont (Traylor et al. 2023a).

## ﻿Discussion

The Coleoptera lists provided here join others in assembling the natural history information of saproxylic invertebrates in the southeastern USA. Hanula (1996) provided a partial list of wood-feeding insects (Blattodea: Termitoidea, Coleoptera, Lepidoptera, and Hymenoptera) for the region, and others assembled similar lists for earthworms (Oligochaeta; Hendrix 1996); mites (Orbatida, Prostigmata, and Mesostigmata; Johnston and Crossley 1996); and millipedes (Diplopoda), centipedes (Chilopoda), and land-snails (various Gastropoda; Caldwell 1996). Snider (1996) compiled an overview of southeastern Collembola occurring in deadwood. Ferro et al. (2012a) provided habitat details and photographs of 71 Coleoptera species associated with various deadwood types and/or forest disturbance categories in Great Smoky Mountains National Park. Ferro et al. (2012a) additionally reviewed the saproxylic literature for the region and wrote a brief history of worldwide saproxylic research. Moreover, published works providing habitat associations and distributional information are available for better known and economically important groups such as Buprestidae (Nelson et al. 2008), Cerambycidae (Lingafelter 2007), and Curculionidae: Scolytinae (Wood 1982). Aside from those referenced above, few resources exist for classifying invertebrate species as saproxylic, which may be a major impediment for deadwood-biodiversity research in the region. Thus, the lists that we present add to the foundation of knowledge about the region’s saproxylic fauna, which will hopefully facilitate further biodiversity research.

An efficient way of gaining knowledge about host range and microhabitat usage for a wide variety of species is emergence experiments, where deadwood pieces are either created or chosen in the field, typically removed from the field after a period of time, and placed into chambers where insects will eventually emerge (methods reviewed in Ferro and Carlton 2011). These types of experiments are useful for inventorying the species using specific types of resources and comparing biodiversity patterns along environmental, spatial, or temporal gradients. For example, studies have examined the saproxylic fauna inhabiting deadwood in response to tree species, decomposition stage, diameter class, vertical stratification, seasonality, sun exposure, flooding, fire exposure, and native/non-native status (Ulyshen and Hanula 2009a, 2010; Ferro et al. 2009, 2012a; Ulyshen et al. 2010, 2012, 2018, 2020; Ferro and Gimmel 2014; Ulyshen 2014; Seibold et al. 2016; Seibold et al. 2018; Vogel et al. 2020; Lettenmaier et al. 2022). Much of the natural history information and species records included in our lists was partially or entirely learned from emergence studies. Such studies are invaluable for providing information about a species’ use of particular habitats. For example, no habitat information was known of the canopy specialist, *Tenebroidessemicylindricus* (Trogossitidae), until it emerged in numbers from dead wood removed from tree crowns (Ulyshen and Hanula 2009a). These studies, however, should be used to complement more thorough investigations of a species’ biology, and documenting the detailed natural history of species should be prioritized when opportunities arise. For example, the fascinating and possibly unique life cycle of *Micromalthusdebilis* (Micromalthidae) would have gone unnoticed were it not for the curiosity and careful study of Barber (1913a, b).

Producing a regional annotated checklist for saproxylic invertebrates would be a great goal for future researchers and would greatly facilitate research in this field. However, we are far from understanding the southeastern fauna well enough to accomplish this, and this is likely the case for other regions as well. From the 293 taxa for which we provide saproxylic habits, 29 species (9.9%) are considered saproxylic solely on taxonomic placement (i.e., no information exists but all congeners with known biology are saproxylic), and 68 species (23.2%) are recorded for the first time in the state of Georgia. Moreover, the extent of natural history known for many species included in our list is limited to a single rearing or emergence event. Thus, very basic biological and distributional information about saproxylic Coleoptera is lacking, and the same applies for other invertebrate groups as well. Filling this information gap is essential for understanding the responses of biodiversity to changing forest conditions and assessing conservation needs.

## ﻿Additional information

### Author ORCIDs

Clayton R. Traylor https://orcid.org/0000-0001-7798-7198

J. Winston Cornish https://orcid.org/0009-0002-3819-2936

Joseph V. McHugh https://orcid.org/0000-0002-7954-6254

### Data availability

All of the data that support the findings of this study are available in the main text or Supplementary Information.

## Supplementary materials

Supplementary material 1 List of beetle taxa with deadwood associations from experimental studies in the southeastern USA Data type xlsx Explanation note Taxa were included if they were collected or emerged from deadwood, or if they were considered saproxylic (see Methods in main text). For taxa collected from deadwood, we provide tree type, collection types (emergent: E, hand-collected: H), and references for each record. For taxa considered saproxylic, we provide references for each study it occurred in. More information on the references can be found in Table 1 of the main text. https://binary.pensoft.net/file/1280407This dataset is made available under the Open Database License (http://opendatacommons.org/licenses/odbl/1.0/). The Open Database License (ODbL) is a license agreement intended to allow users to freely share, modify, and use this Dataset while maintaining this same freedom for others, provided that the original source and author(s) are credited.Clayton R. Traylor, Michael D. Ulyshen, J. Winston Cornish, Gabriel Tigreros, Joseph V. McHugh
